# Vascular endothelial growth factor levels in tuberculosis: A systematic review and meta-analysis

**DOI:** 10.1371/journal.pone.0268543

**Published:** 2022-05-25

**Authors:** Amene Saghazadeh, Nima Rezaei

**Affiliations:** 1 Research Center for Immunodeficiencies, Children’s Medical Center, Tehran University of Medical Sciences, Tehran, Iran; 2 Systematic Review and Meta-Analysis Expert Group (SRMEG), Universal Scientific Education and Research Network (USERN), Tehran, Iran; 3 Department of Immunology, School of Medicine, Tehran University of Medical Sciences, Tehran, Iran; 4 Network of Immunity in Infection, Malignancy and Autoimmunity (NIIMA), Universal Scientific Education and Research Network (USERN), Tehran, Iran; Karolinska Institutet, SWEDEN

## Abstract

**Background:**

Changes in endothelial function are implicated in the spread of tuberculosis (TB). Studies suggest a role for the vascular endothelial growth factor (VEGF) in TB-related endothelial function changes. However, the findings of studies investigating the VGEF profile in TB are not consistent, and no formal systematic review and meta-analysis exists summarizing these studies.

**Methods:**

We did a meta-analysis of studies assessing VEGF levels in patients with TB. A systematic search on June 25, 2021, was conducted for eligible studies that made VEGF measurements in an unstimulated sample, e.g., a blood fraction (plasma or serum), cerebrospinal fluid (CSF), pleural effusion (PE), or bronchoalveolar lavage fluid, and ascites or pericardial fluid for patients with TB and controls without TB. Also, studies that made simultaneous measurements of VEGF in blood and PE or CSF in the same patients with TB were included. Longitudinal studies that provided these data at baseline or compared pre-post anti-tuberculosis treatment (ATT) levels of VEGF were included. The primary outcome was the standardized mean difference (SMD) of VEGF levels between the comparison groups.

**Results:**

52 studies were included in the meta-analysis. There were 1787 patients with TB and 3352 control subjects of eight categories: 107 patients with transudative pleural effusion, 228 patients with congestive heart failure (CHF)/chronic renal failure (CRF), 261 patients with empyema and parapneumonic effusion (PPE), 241 patients with cirrhosis, 694 healthy controls (with latent TB infection or uninfected individuals), 20 patients with inactive tuberculous meningitis (TBM), 123 patients with non-TBM, and 1678 patients with malignancy. The main findings are as follows: (1) serum levels of VEGF are higher in patients with active TB compared with healthy controls without other respiratory diseases, including those with latent TB infection or uninfected individuals; (2) both serum and pleural levels of VEGF are increased in patients with TPE compared with patients with transudative, CHF/CRF, or cirrhotic pleural effusion; (3) ascitic/pericardial fluid, serum, and pleural levels of VEGF are decreased in patients with TB compared with patients with malignancy; (4) pleural levels of VEGF are lower in patients with TPE compared with those with empyema and PPE, whereas serum levels of VEGF are not different between these patients; (5) both CSF and serum levels of VEGF are increased in patients with active TBM compared with controls, including patients with inactive TBM or non-TBM subjects; (6) post-ATT levels of VEGF are increased compared with pre-ATT levels of VEGF; and (7) the mean age and male percentage of the TB group explained large and total amount of heterogeneity for the meta-analysis of blood and pleural VEGF levels compared with healthy controls and patients with PPE, respectively, whereas these moderators did not show any significant interaction with the effect size for other analyses.

**Discussion:**

The important limitation of the study is that we could not address the high heterogeneity among studies. There might be unmeasured factors behind this heterogeneity that need to be explored in future research. Meta-analysis findings align with the hypothesis that TB may be associated with abnormal vascular function, and both local and systemic levels of VEGF can be used to trace this abnormality.

## Introduction

Despite a century of intense medical research, the burden of tuberculosis (TB) remains alarming due to its causing pathogen, mycobacterium tuberculosis (*M*. *tb*), that interferes with the biology of molecules and cells to seriously affect the function of vital organs and systems. This pathogen has been shown to invade the circulatory system, respiratory system, central nervous system, lymphatic system, gastrointestinal system, and genitourinary system. There are different, yet unproven, mechanisms proposed to handle such an invasion. However, the hypothesis of vascular involvement appears to be functional. In particular, the development of vascular complications and elevation of adhesion molecules [1] in patients with TB support this hypothesis.

Moreover, since 2000, research has shown that the protein levels of vascular endothelial growth factor (VEGF), which correlates with angiogenesis and is traditionally considered a marker of malignant situations [2], are altered in TB. However, the findings are not consistent, and no pooled analysis exists investigating the VEGF profile in patients with TB. This inconsistency might lie in different samples used for VEGF measurement, including the cerebrospinal fluid (CSF), the peripheral blood, the pleural effusion (PE), the ascites or pericardial fluid, and different conditions served as a control condition, such as apparently healthy conditions, malignancy, empyema and parapneumonic effusion (PPE), transudate effusion, etc.

Elucidating the profile of VEGF will be helpful to diagnosis of TB and its differentiation from the aforementioned clinical conditions. In addition, the decrease or increase of this growth factor might shed light on the pathogenesis of TB and therapeutic implications. This is a systematic review and meta-analysis of studies investigating the VEGF profile in patients with TB.

## Methods

We have prepared the present study according to the Preferred Reporting Items for Systematic Reviews and Meta-Analyses (PRISMA) statement [3] (S1 Checklist).

### Search strategy

We searched PubMed, Scopus, and Web of Science using the search terms: (tuberculosis OR tuberculous) AND (vascular endothelial growth factor OR VEGF). The search was conducted on June 25, 2021, and the analyses were finalized on July 20, 2021. There was no restriction applied to language or date of publication. We also searched Google Scholar for any additional studies missing from the database search.

### Selection criteria

#### Publication type

Original studies that met the eligibility criteria were included.

#### Study design

Observational studies that made VEGF measurements for patients with TB and controls without TB were included. Also, observational studies that made simultaneous measurements of VEGF in blood and PE or CSF in the same patients with TB were included. Longitudinal studies that provided these data at baseline or compared pre-post anti-tuberculosis treatment (ATT) levels of VEGF were included.

#### Participants

Studies met the inclusion criteria if they (1) enrolled patients with TB of any age group or gender; (2) assessed VEGF levels in an unstimulated sample, e.g., a blood fraction (plasma or serum), CSF, PE or bronchoalveolar lavage fluid (BALF), and ascites or pericardial fluid; (3) compared VEGF measurements in the TB group with those of a control group, e.g., healthy controls including individuals with latent TB infection or TB-uninfected individuals without other respiratory diseases or infections, patients with malignant pleural effusion (MPE), patients with malignant ascites, patients with empyema or PPE, patients with transudate, congestive heart failure (CHF)/chronic renal failure (CRF), or cirrhotic effusion, or compared blood VEGF measurements with pleural or central VEGF measurements that were obtained simultaneously from the same patients with TB, or compared pre-ATT with post-ATT levels of VGEF in the same patients with TB; and (4) provided sufficient data to calculate the mean difference of VEGF levels between the two comparison groups.

### Data extraction

We used the excel spreadsheets to extract the following data from each of the included studies: the link to the study; title; first author; year of publication; the condition, mean age, male percentage, and HIV-positive rate for each of the comparison groups; ATT status for the TB group; the sample and the assay used for VEGF measurement; the scale of VEGF measurement; and the number of participants, mean, and standard deviation (SD) of VEGF measures for each of the comparison groups. When the VEGF data were not available in the article and related supplementary material, we sent the first or corresponding author an e-mail to provide us with the required data. In addition, we used any graphs that contained data and alternative measures, e.g., median, range, interquartile range, or 95% confidence interval (CI), to estimate the mean and SD.

### Quality assessment

The Newcastle-Ottawa Scale (NOS) was used for assessing the quality of included studies [4]. We appraised the studies using a platform that comprised four one-star items related to the selection of the case and control groups, two one-star items (age and sex) related to the comparability of the case and control groups, one two-star item related to the outcome assessment, and one one-star item related to the statistical tests used to analyze the data. The study quality was, thus, ranged from 0 to 9 and assigned to a high, moderate, and low when the quality scores were 7–9, 4–6, and 0–3, respectively.

### Outcomes

The primary outcome was the difference in VEGF levels. The samples of interest included the ascites or pericardial fluid, BALF or PE, CSF, and plasma or serum. The between-group comparison groups of interest were: (1) patients with active TB vs. healthy controls, including individuals with latent TB infection or TB-uninfected subjects, without other respiratory diseases or TB-like diseases; (2) patients with TB ascites vs. patients with malignant ascites; (3) patients with TPE vs. patients with MPE, or patients with empyema and PPE, or patients with transudate, CHF/CRF, or cirrhotic effusion; and (4) patients with active TBM vs. patients with inactive TBM or non-TBM. The within-subject analyses of interest were blood vs. pleural or central VEGF levels and pre-ATT vs. post-ATT measures of VEGF in the same patients with TB.

### Data analysis

A meta-analysis was run for a comparison of interest when three or more observations were available. The standardized mean difference (SMD) with Hedges’ g was chosen as the measure of the effect. The effect size was calculated using a random-effects model with a restricted maximum-likelihood (REML) and considered a large, moderate, and small effect with respect to the SMD values of 0.8, 0.5, and 0.2, respectively. The heterogeneity among the studies included in a meta-analysis was assessed using Cochrane’s Q, tau-squared, and I-squared (*I*^2^). Cochrane’s Q test quantifies total variance and generates a *p*-value that determines the heterogeneity is present. Tau-squared indicates the true variance that is the between-study variance, while *I*^2^ represents the percentage of the total variance that is due to the true variance. The degree of heterogeneity is said to be low, moderate, and high, with *I*^2^ values of 25%, 50%, and 75%.

Eggers’ test was used to examine the funnel plot asymmetry. Whenever this test was significant with a *p*-value of less than 0.1, we used the trim and fill method to correct the funnel plot and adjust the effect size for potential publication bias.

Sensitivity analysis using the leave-one-out method was performed to investigate the robustness of the SMD. If the influence analysis revealed that the SMD was sensitive to the effect size of a single observation, we repeated the original analysis after removing the influential observation(s).

To investigate the possible sources of heterogeneity, subgroup analyses were done using the geographic location, the assay used (ELISA vs. not ELISA), and the sample (plasma vs. serum) as predictors. Moreover, meta-regression was considered to evaluate the interaction between potential moderators of the mean age or male percentage in the TB group and the effect size of the difference in VEGF levels.

All meta-analyses, subgroup analyses, and meta-regressions were conducted using the R (version 4.0.5).

## Results

### Study characteristics

**Fig 1:**
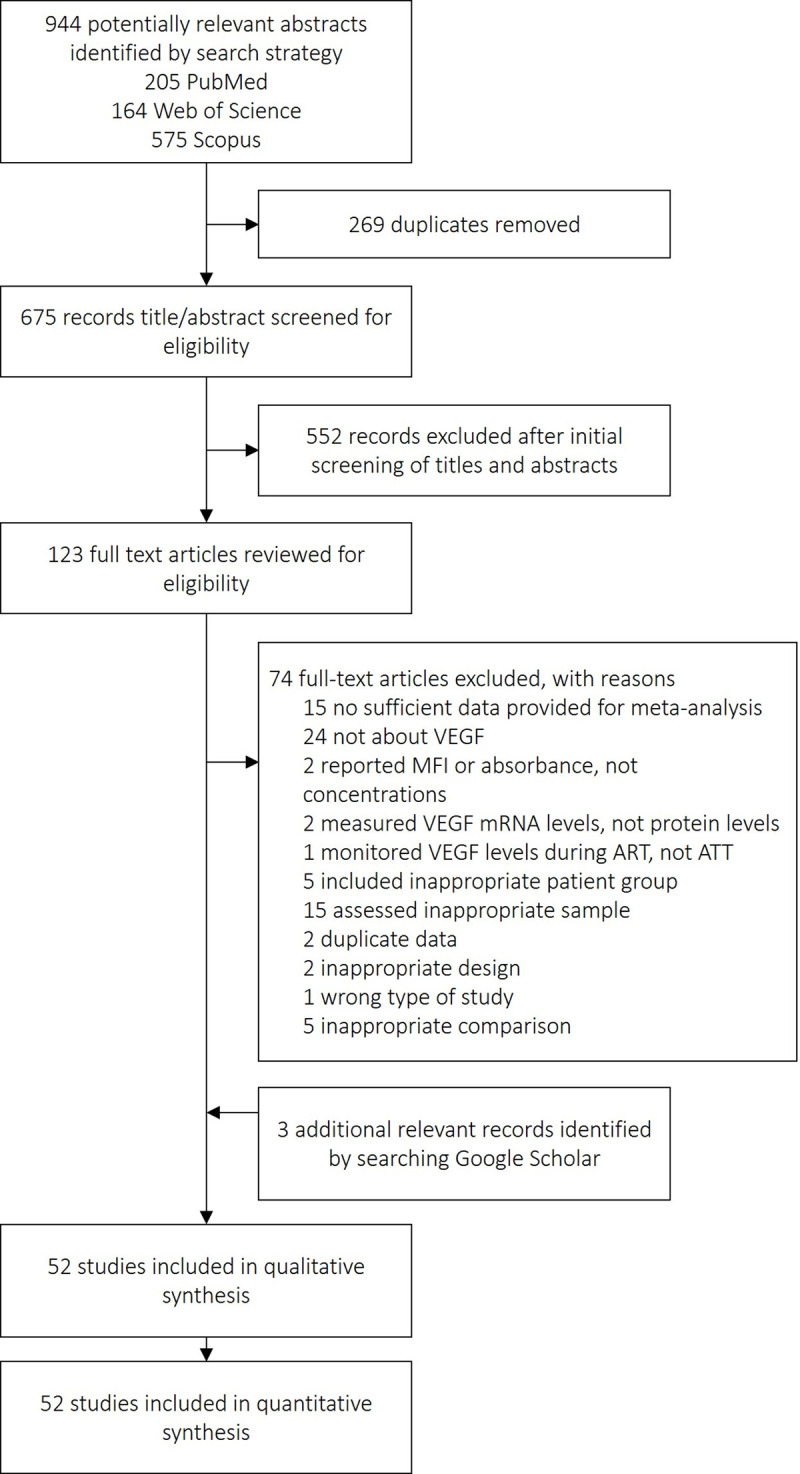
Study selection.

The systematic search identified 123 articles as potentially eligible to be included in our review (Fig 1), of which 74 studies were excluded with the reasons described in S1 Text. Three additional records were found by searching other sources. Finally, 52 studies were included in the meta-analysis [5–56]. S1 Table summarizes the characteristics of studies. There were 1787 patients with TB and 3352 control subjects of eight categories: 107 patients with transudative pleural effusion, 228 patients with CHF/CRF, 261 patients with empyema and PPE, 241 patients with cirrhosis, 694 healthy controls (with latent TB infection or uninfected individuals), 20 patients with inactive TBM, 123 patients with non-TBM, and 1678 patients with malignancy. Thirty-two studies assessed a blood fraction (plasma or serum), thirty-two studies assessed the pleural fluid, five studies assessed CSF, and three studies assessed the ascitic or pericardial fluid. Fourteen studies examined pleural and blood simultaneously, and four studies examined CSF and blood simultaneously. Forty-three studies (84.6%) used ELISA for VEGF measurement, six studies (11.5%) used Luminex and Bio-Plex platforms, and one (1.9%) study used Simoa-based technology for VEGF measurement. Two studies did not report the assay used for VEGF measurement. The quality of studies was rated as high, moderate, and for low 40 (76.9%), 10 (19.2%), and 2 (3.9%) studies, respectively (S2 Table). Overall, the quality of the included studies was good, with an average NOS of 7.17.

### Meta-analysis

**Fig 2:**
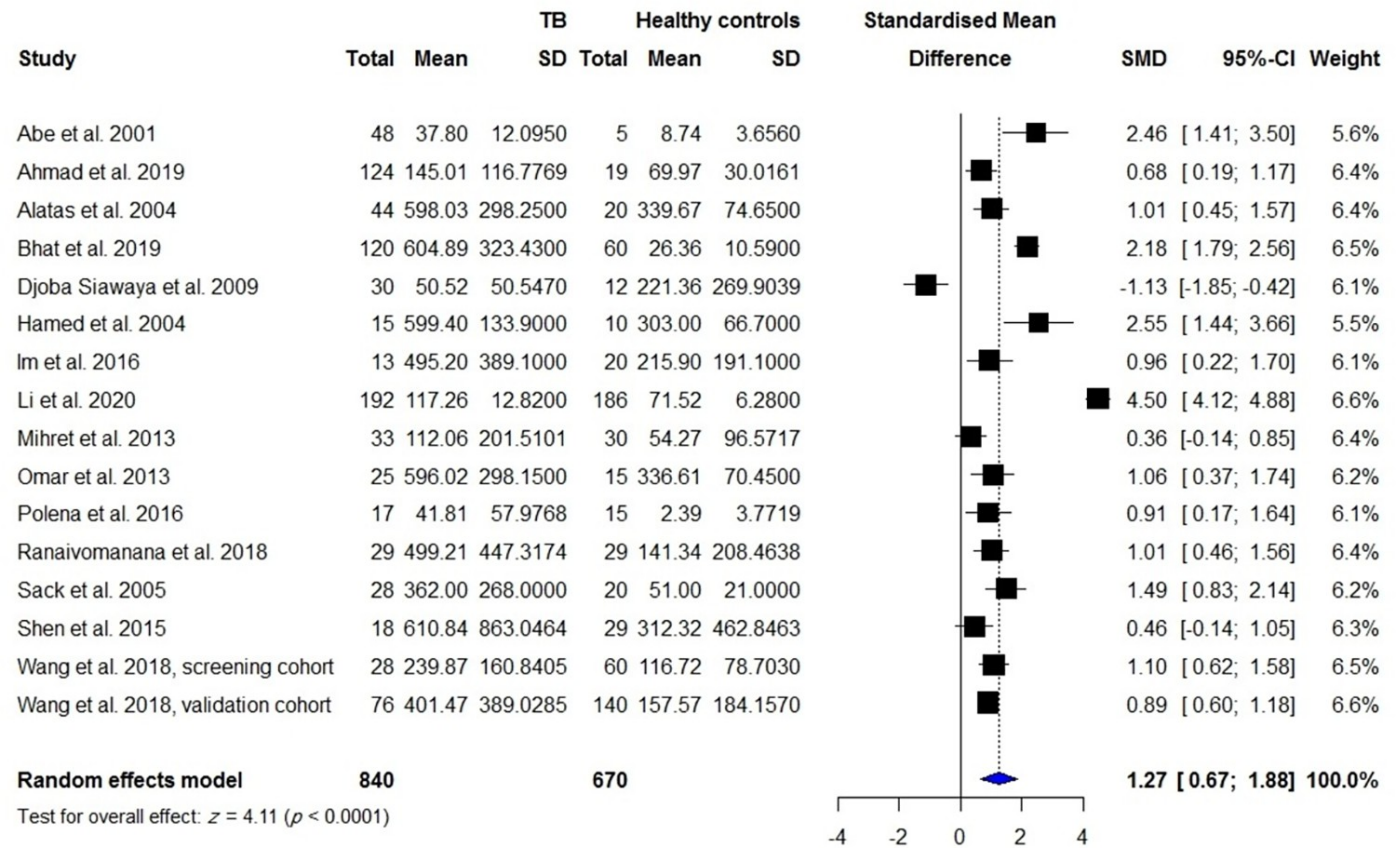
Meta-analysis of blood VEGF levels in patients with active TB vs. healthy controls, including latent TB infection and TB-uninfected individuals.

**Fig 3:**
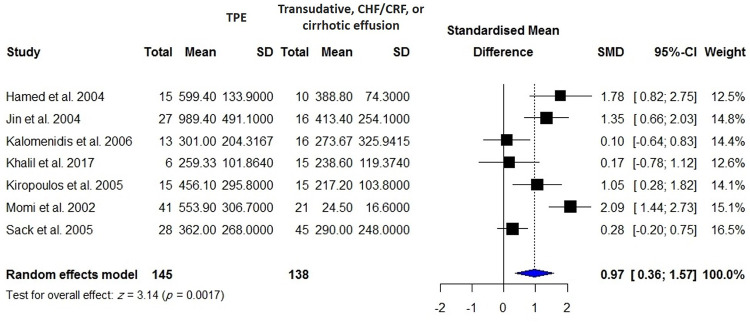
Meta-analysis of blood VEGF levels in patients with TPE vs. patients with transudative, CHF/CRF, or cirrhotic effusion.

**Fig 4:**
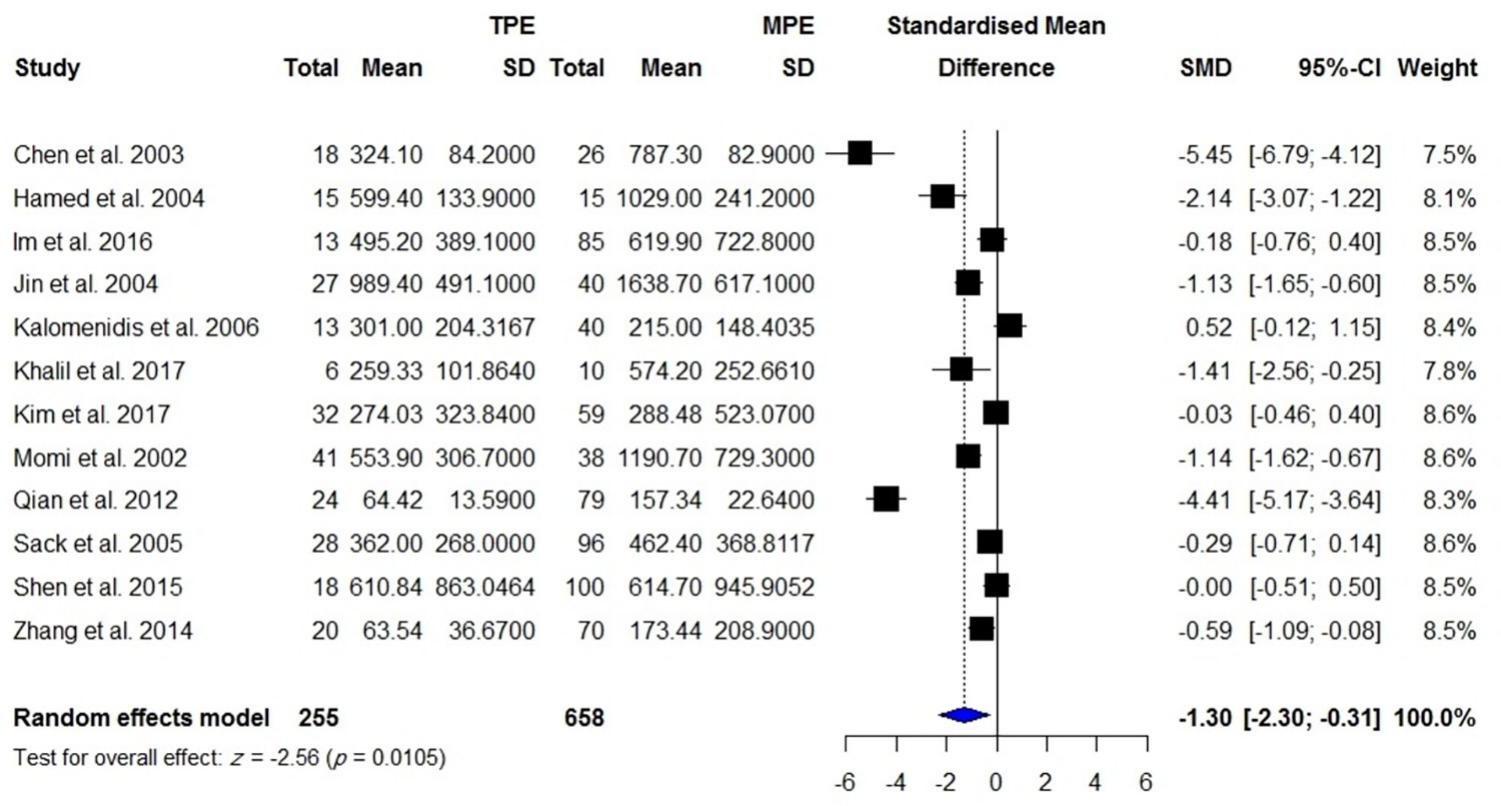
Meta-analysis of blood VEGF levels in patients with TPE vs. patients with MPE.

**Fig 5:**
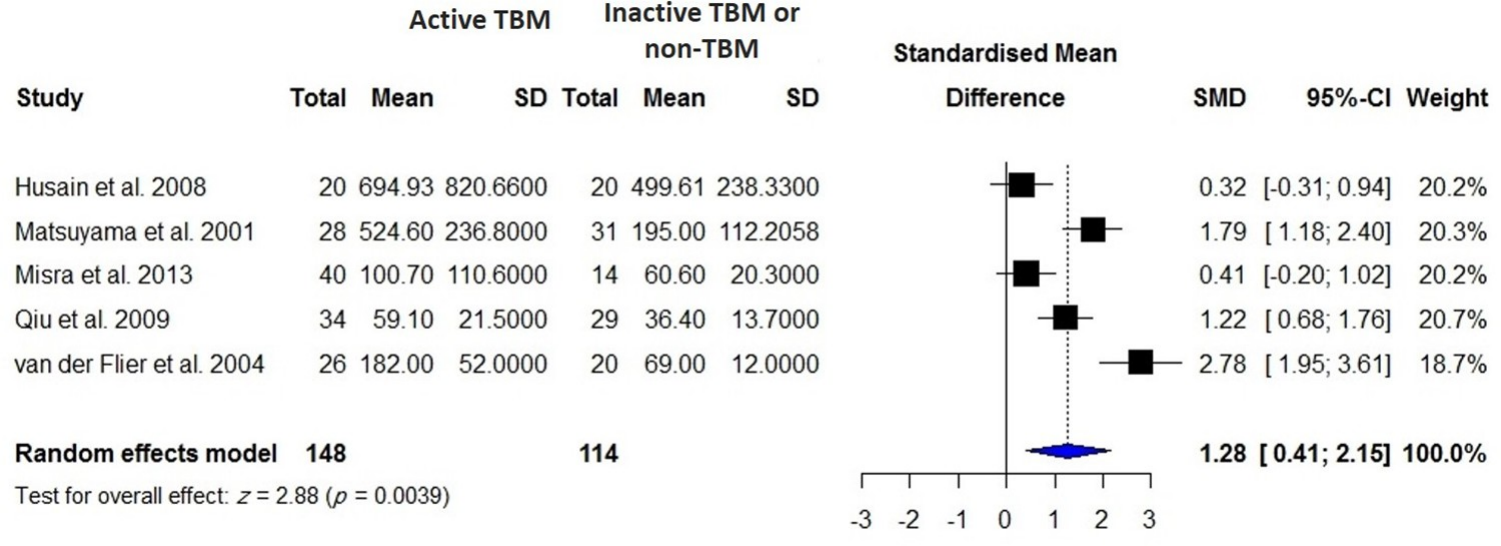
Meta-analysis of blood VEGF levels in patients with active TBM vs. controls including inactive TBM or non-TBM individuals.

**Fig 6:**
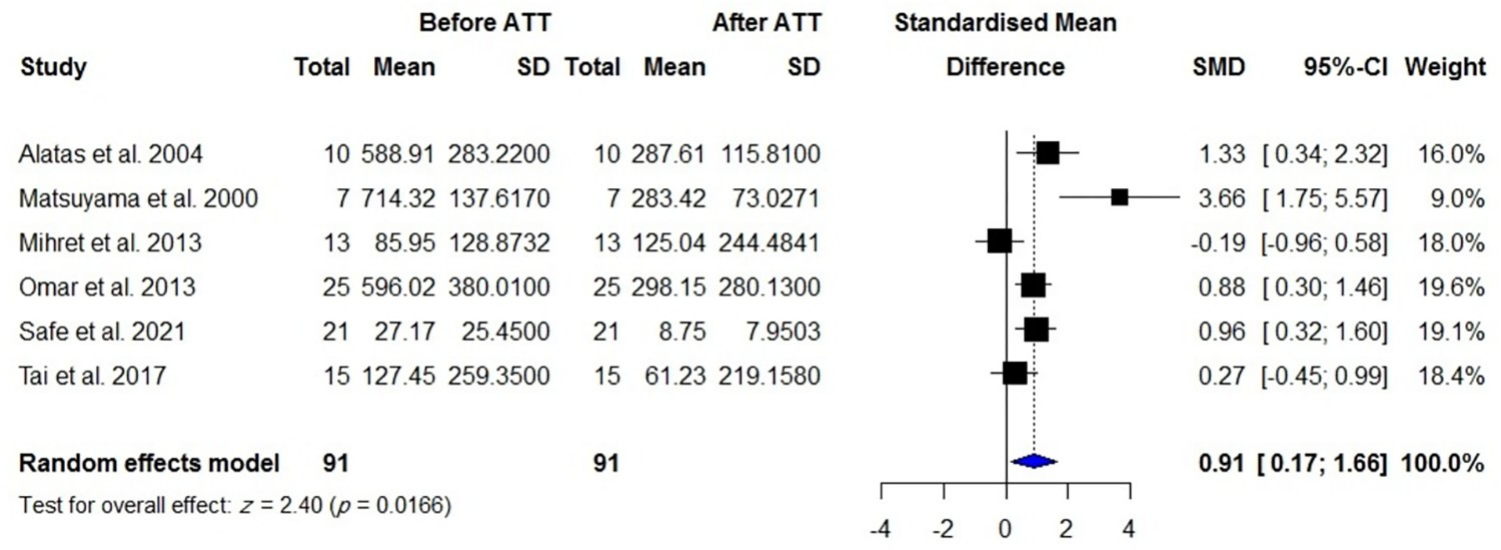
Meta-analysis of pre-post treatment levels of blood VEGF in patients with TB.

VEGF concentrations in the blood were reported in 840 patients with active TB compared with 670 healthy controls including individuals with latent TB infection and TB-uninfected subjects, 255 patients with TPE compared with 658 patients with MPE, 106 patients with TPE compared with 150 patients with PPE, 145 patients with TPE compared with 138 patients with transudate effusion and effusion due to CHF and cirrhosis, and 148 patients with active TBM compared with 114 control subjects including individuals with inactive TBM or non-TBM. There were significantly higher VEGF levels in patients with active TB than controls (SMD = 1.27; 95% CI, 0.67 to 1.88; *I*^2^ = 96%; *p* < 0.0001) (Table 1, Fig 2). In addition, blood VEGF levels were increased in patients with TPE compared with those with transudate effusion or effusion due to CHF and cirrhosis (SMD = 0.97; 95% CI, 0.36 to 1.57; *p* = 0.002) (Fig 3), whereas blood VEGF levels were decreased in patients with TPE compared with those with MPE (SMD = -1.30; 95% CI, 2.30 to 0.31; *I*^2^ = 94%; *p* = 0.011) (Fig 4). No difference in blood VEGF levels was found between patients with TB and patients with PPE (*p* = 0.850; S1 Fig). Blood VEGF levels were higher in patients with TBM than controls with inactive TBM/non-TBM (SMD = 1.28; 95% CI, 0.41 to 2.15; *I*^2^ = 87%; *p* = 0.004) (Fig 5). In addition, pre- and post-ATT VEGF measurement was available for 91 patients with TB. Pre-ATT VEGF levels in blood were significantly higher than post-ATT VEGF levels (SMD = 0.91; 95% CI, 0.17 to 1.66; *I*^2^ = 73%; *p* = 0.0166) (Fig 6).

**Table 1:** Meta-analyses of VEGF in TB.

Sample	Number of observations	Case		Control		SMD [95% CI]	p-value	Heterogeneity			Egger’s test
		Condition	n	Condition	n			Q statistic (DF; p value)	τ^2^	I^2^	t statistic (p value)
Blood	16	Active TB	840	Healthy control with latent TB or uninfected control	670	1.272 [0.666; 1.877]	**<0.0001**	383.01 (15; <0.0001)	1.421	96.1	1.455 (0.219)
	7	TPE	145	Transudate effusion, effusion due to CHF, and effusion due to cirrhosis	138	0.966 [0.364; 1.569]	**0.002**	31.31 (6; <0.0001)	0.516	80.8	NA
	12	TPE	255	Malignancy	658	-1.304 [-2.302; -0.306]	0.011	193.12 (11; <0.0001)	2.974	94.3	-2.69 (0.023)
	5	TPE	106	Empyema and PPE	150	-0.063 [-0.722; 0.595]	0.850	24.73 (4; <0.0001)	0.445	83.8	NA
	5	Active TBM	148	Inactive TBM or non-TBM	114	1.280 [0.410; 2.150]	**0.004**	31.35 (4; <0.0001)	0.875	87.2	NA
CSF	4	Active TBM	138	Inactive TBM or non-TBM	123	1.514 [0.887; 2.142]	**<0.0001**	12.96 (3; 0.0047)	0.316	76.9	NA
	3	Active TBM	118	VM	64	1.320 [0.735; 1.904]	**<0.0001**	5.62 (2; 0.0601)	0.166	64.4	NA
Ascitic or pericardial fluid	3	TB	208	Malignancy	532	-1.542 [-1.914; -1.184]	**<0.0001**	4.34 (2; 0.1142)	0.050	53.9	NA
PE	13	TPE	298	Lung cancer	586	-1.344 [-2.047; -0.642]	**0.0002**	157.57 (12; <0.0001)	1.547	92.4	-0.647 (0.531)
	25	TPE	494	Malignancy	997	-1.473 [-2.055; -0.891]	**<0.0001**	259.98 (24; <0.0001)	2.047	90.8	-1.781 (0.088)
	12	TPE	193	Empyema and PPE	224	-0.401 [-0.850; 0.048]	0.0797	44.50 (11; <0.0001)	0.437	75.3	-1.846 (0.095)
	18	TPE	315	Transudate effusion, effusion due to CHF/CRF, and effusion due to cirrhosis	254	1.502 [1.111; 1.895]	**<0.0001**	59.81 (17; <0.0001)	0.474	71.6	1.937 (0.071)

Summary of meta-analyses of VEGF levels by sample: Blood, cerebrospinal fluid (CSF), ascitic or pericardial fluid, and pleural effusion (PE). Bonferroni-corrected *p*-value was 0.004. Significant *p*-values are in bold.

N, number, SMD, standardized mean difference; CI, confidence interval; DF, degree of freedom; TB, tuberculosis; TPE, tuberculous pleural effusion; TBM, tuberculous meningitis; CHF, congestive heart failure; CRF, chronic renal failure

**Fig 7:**
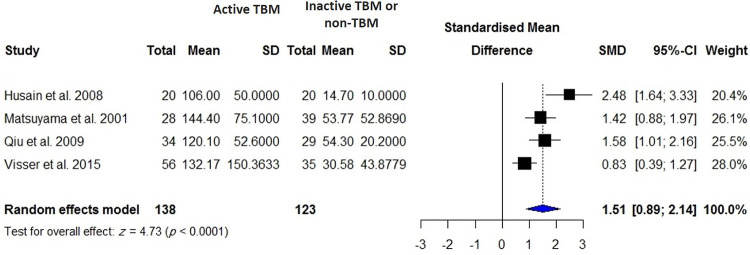
Meta-analysis of CSF VEGF levels in patients with active TBM vs. controls including inactive TBM or non-TBM individuals.

CSF VEGF was measured in 138 patients with TBM and 123 control subjects including patients with inactive TBM and non-TBM. There were significantly higher VEGF levels in patients with TBM than control subjects (SMD = 1.51; 95% CI, 0.89 to 2.14; *I*^2^ = 77%, *p* < 0.0001) (Fig 7). The SMD remained significant (*p* < 0.0001; S2 Fig) for difference in CSF VEGF levels when patients with TBM (*N* = 118) were compared to those with viral meningitis (*N* = 64). The VEGF levels in patients with TBM were not significantly different in CSF and serum (*p* = 4328; S3 Fig).

VEGF levels in the ascitic or pericardial fluid were evaluated in 208 patients with TB and 532 patients with malignancy. VEGF levels were significantly lower in patients with TB (SMD = -1.55; 95% CI, -1.91 to -1.18; *I*^2^ = 54%; *p* < 0.0001) (S4 Fig).

**Fig 8:**
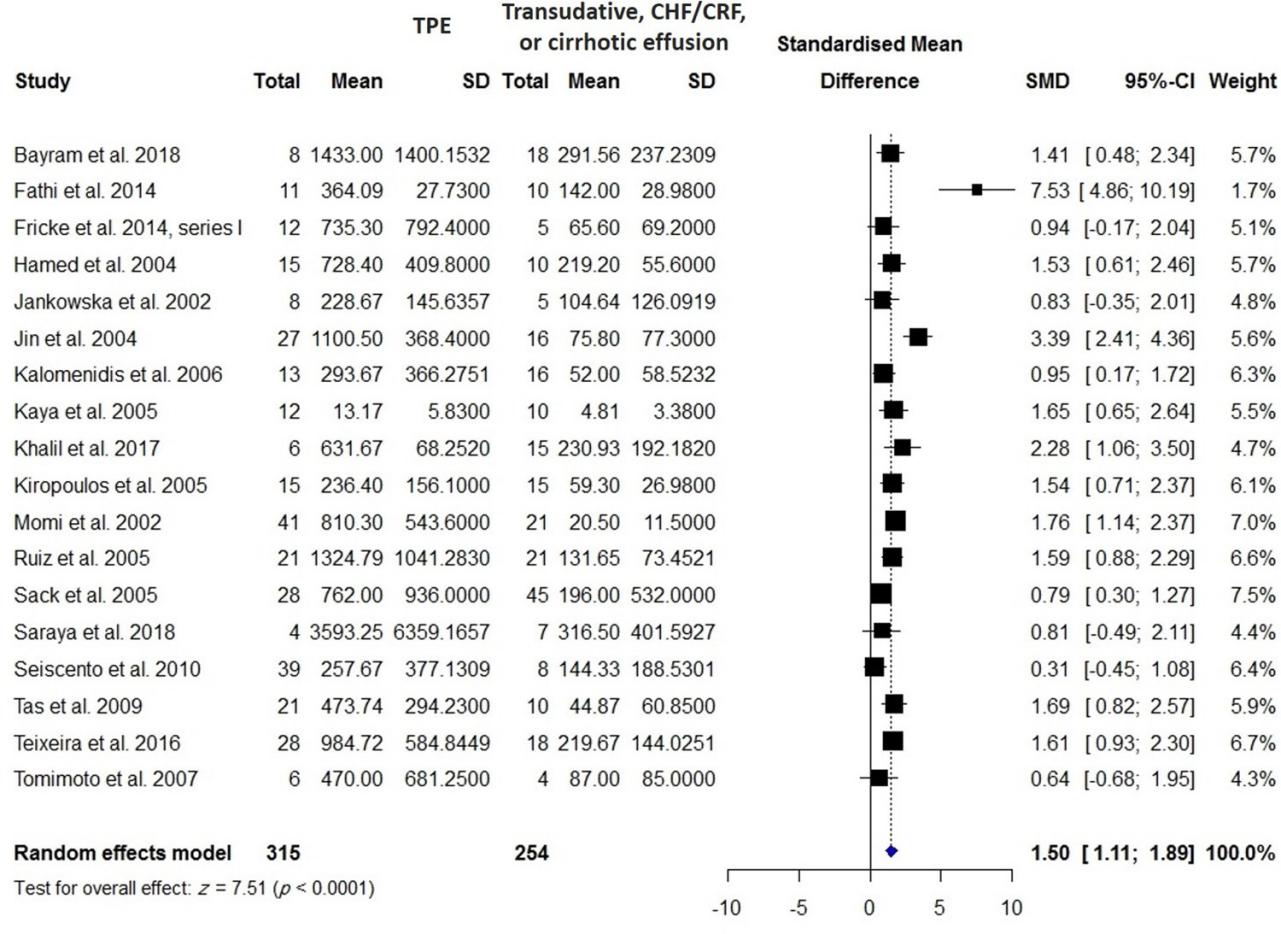
Meta-analysis of blood VEGF levels in patients with TPE vs. patients with transudative, CHF/CRF, or cirrhotic effusion.

**Fig 9:**
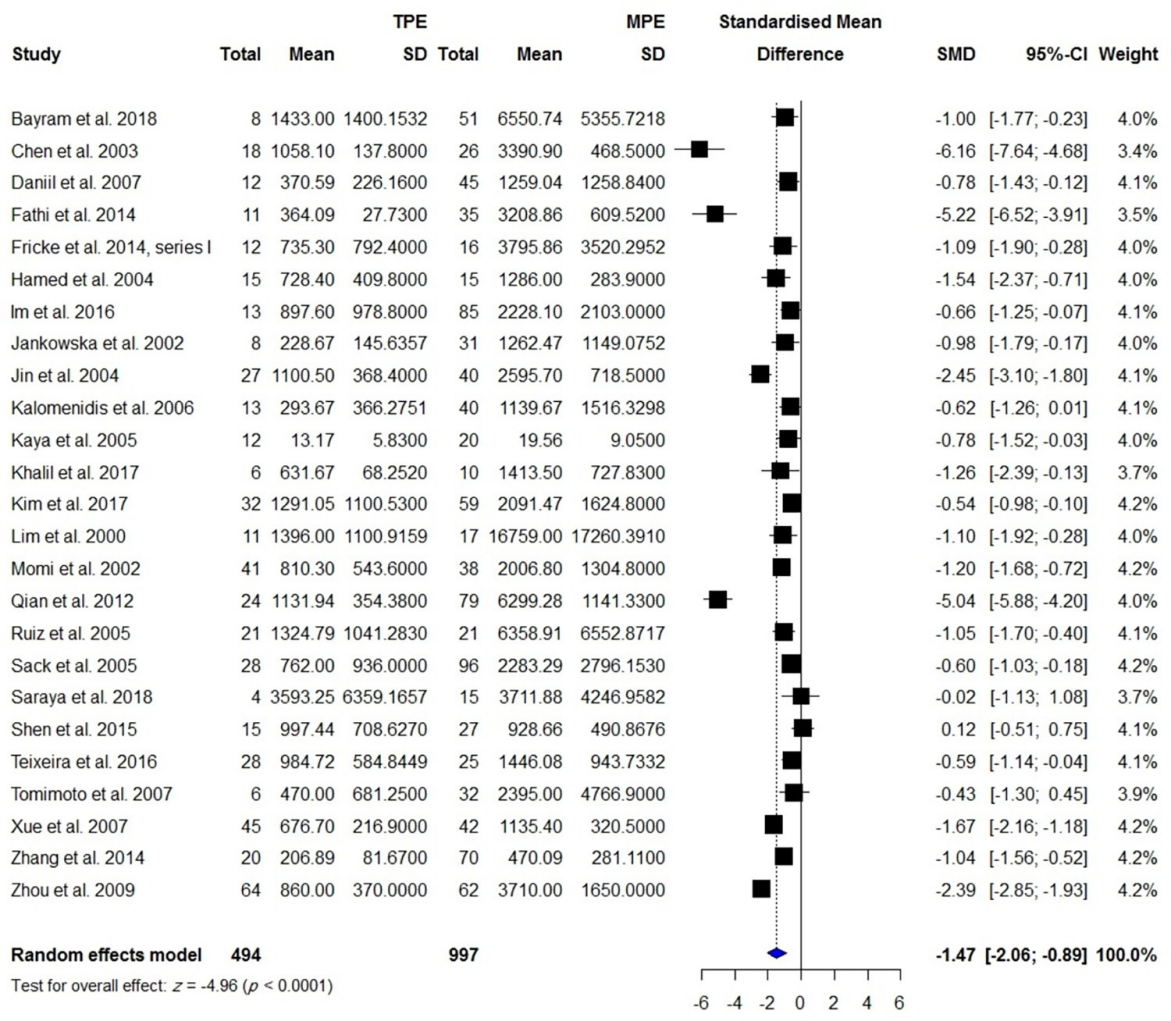
Meta-analysis of blood VEGF levels in patients with TPE vs. patients with MPE.

**Fig 10:**
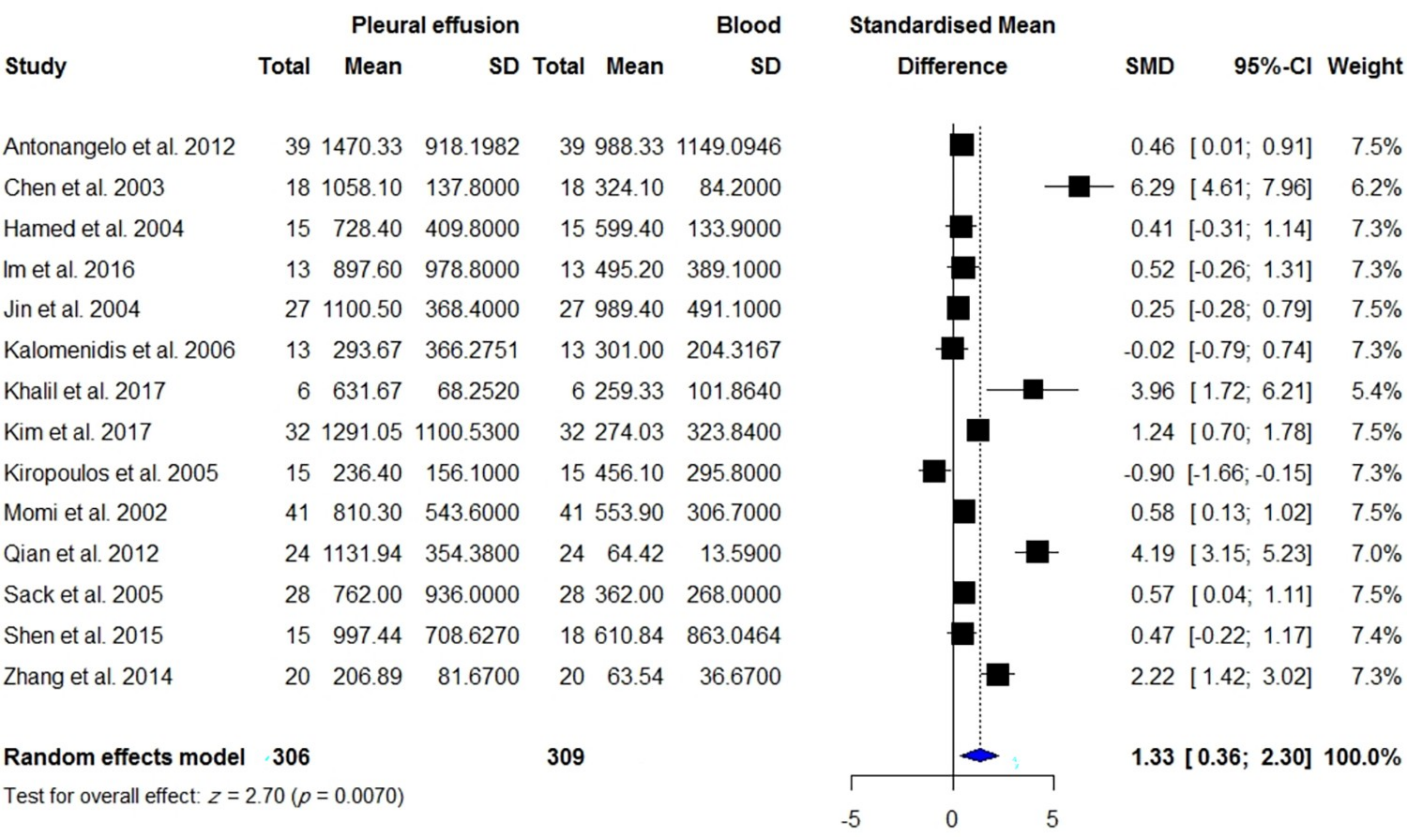
Meta-analysis of pleural VEGF levels vs. blood VGEF levels in the same patients with TPE.

VEGF levels in the BALF or PE were measured in 315 patients with TPE compared with 254 patients with effusion due to CHF/CRF and transudate effusion, 494 patients with TPE compared with 997 patients with malignancy, 298 patients with TPE compared with 586 patients with lung cancer, and 193 patients with TPE compared with 224 patients with PPE. Pleural VEGF levels in patients with TPE were significantly higher than those in patients with effusion due to CHF/CRF and transudate effusion (SMD = 1.5; 95% CI, 1.11 to 1.89; *I*^2^ = 72%; *p* < 0.0001) (Fig 8), whereas they were significantly lower than those in patients with MPE (SMD = -1.47; 95% CI, -2.06 to -0.89; *I*^2^ = 91%; *p* < 0.0001) (Fig 9) and lung cancer (SMD = -1.34; 95% CI, -2.05 to -0.64; *I*^2^ = 92%; *p* = 0.0002) (S5 Fig). There was no significant difference in pleural VEGF levels between patients with TPE and PPE (*p* = 0.0797) (S1 Fig). In patients with TPE, pleural VEGF levels were significantly higher than serum VEGF levels (SMD = 1.33; 95% CI, 0.36 to 2.30; *I*^2^ = 91%; *p* = 0.007) (Fig 10).

### Publication bias

There was evidence of potential publication bias for meta-analyses of difference in blood VEGF levels between patients with TPE and MPE (*p* = 0.023) and of the difference in pleural VEGF levels between patients with TPE and MPE (*p* = 0.088), TPE and PPE (*p* = 0.080), and TPE and transudative effusions (*p* = 0.071). The trim and fill method changed the significance of the difference in blood VEGF levels between patients with TPE and MPE (S6 Fig). With three added studies, it was found to be not significantly different (SMD = -0.44; 95% CI, -1.71 to 0.82; *p* = 0.4927). Moreover, for the difference in pleural VEGF levels between patients with TPE and PPE, the trim and fill method added four studies (S7 Fig), but the effect remained insignificant (SMD = -0.03; 95% CI, -0.53 to 0.48; *p* = 0.9130). The trim and fill method did not change the significance of the effect for the difference in pleural VEGF levels between patients with TPE and MPE and between patients with TPE and patients with transudative effusions or effusions due to CHF, CRF, and cirrhosis and the effect sizes remained significantly different since no additional studies needed to be imputed into the analyses.

### Sensitivity analysis

**Fig 11:**
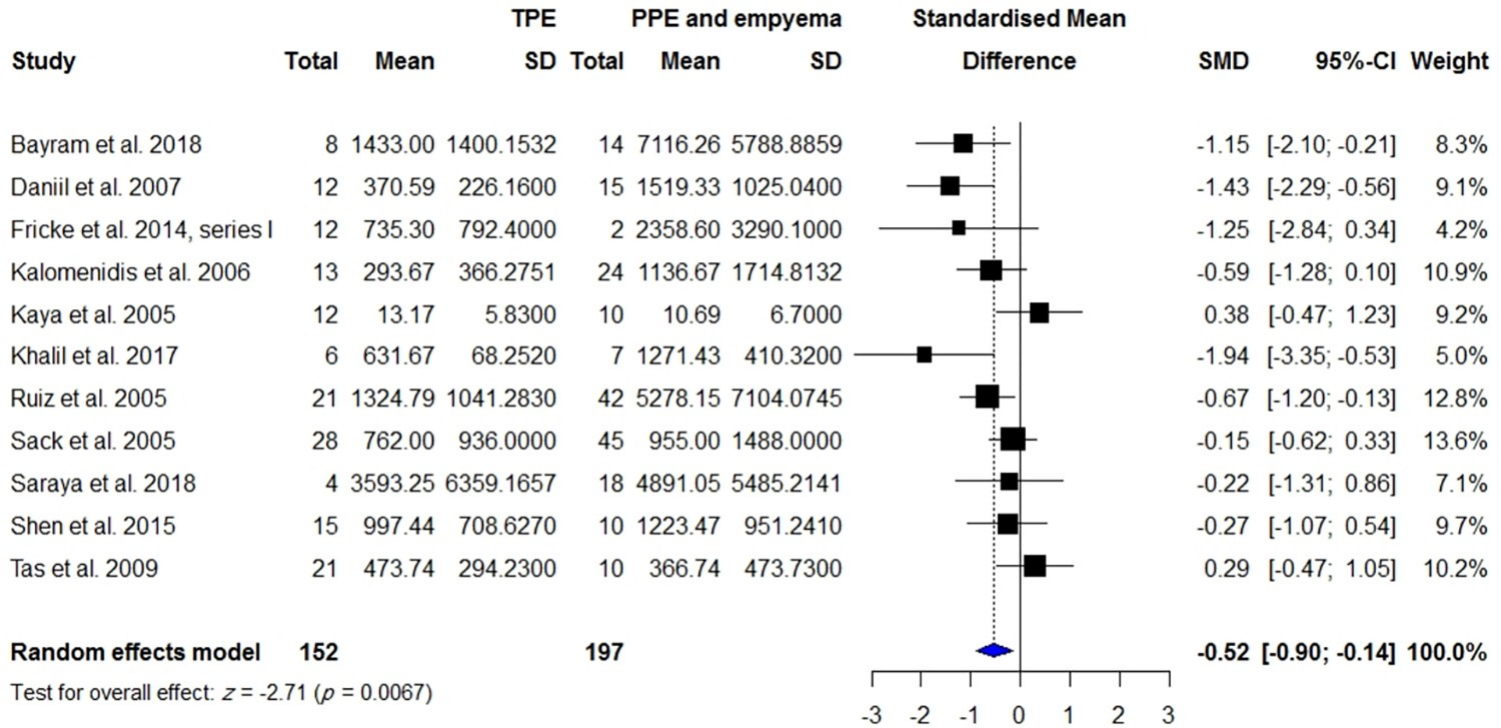
Meta-analysis of pleural VEGF levels in patients with TPE vs. patients with empyema and PPE.

The leave-one-out sensitivity analyses showed that the results of analyses of VEGF levels in the blood for patients with TB compared with healthy controls and for patients with TPE compared with MPE remained significantly robust with SMDs ranging from 1.03 to 1.43 (S8 Fig) and from -0.95 to -1.47 (S9 Fig), respectively. For pleural VEGF levels, the difference remained robust between TPE and lung cancer, between TPE and malignancy, and between TPE and transudative effusion with SMDs ranging from -1.05 to -1.47 (S10 Fig), from -1.30 to -1.53 (S11 Fig), and from 1.35 to 1.57 (S12 Fig) respectively. In addition, higher VEGF levels in PF compared with serum remained significantly different for patients with TPE, with SMDs ranging from 0.95 to 1.44 (S13 Fig). However, the leave-one-out method revealed the SMD of difference in pleural VEGF levels between TPE and PPE to be significant after removing an influential study by Momi and colleagues (SMD = -0.52; 95% CI, -0.90 to -0.15; *I*^2^ = 55%; *p* = 0.0067) (S14 Fig and Fig 11).

### Subgroup analysis

Subgroup analyses revealed that higher VEGF levels in pleural effusion than serum in patients with TB were found in studies of East Asia & Pacific (*p* = 0.0048), not in studies of Europe & Central Asia (*p* = 0.9158), Latin America & Caribbean (*p* = 0.8027), and Middle Ease & North Africa (*p* = 0.1699) (S15 Fig). In addition, blood VEGF levels were increased in patients with active TB compared with healthy controls in subgroup of studies which took place in East Asia & Pacific (*p* = 0.0008), measured VEGF in serum (*p* < 0.0001), and used ELISA for VEGF measurement (*p* < 0.0001) (S16–S18 Figs). When compared with MPE, higher blood VEGF levels in patients with TPE were found in studies of East Asia & Pacific (*p* = 0.0149), not in Middle Ease & North Africa (*p* = 0.1728) or Europe & Central Asia (*p* = 0.9290) (S19 Fig). Also, pleural VEGF levels were higher in TPE than MPE in studies of East Asia & Pacific (*p* < 0.0001) and Middle East & North Africa (*p* = 0.0030), but not of Latin America & Caribbean (*p* = 6847) and Europe & Central Asia (*p* = 0.0959) (S20 Fig). Although, overall, pleural VEGF levels in TPE were not different from those in PPE (*p* = 0.0797), the difference was significant towards lower VEGF levels in TPE in subgroup of studies of Europe & Central Asia (*p* = 0.0359) and Middle East & North Africa (*p* = 0.0296) (S21 Fig). S3 Table describe the results of subgroup analyses. Higher pleural VEGF levels in TPE compared with transudate, CHF/CRF, or cirrhotic effusion were observed for both Europe & Central Asia (*p* < 0.0001) and Middle East & North Africa (*p* < 0.0001) (S22 Fig).

### Meta-regression

When compared with MPE, the mean age of the TB group significantly explained a large amount of heterogeneity (*R*^2^ = 70.5%) for blood VEGF levels (*k* = 6, estimated index 0.0770; *p* = 0.0047). In addition, the male percentage of the TB group was accounted for the total amount of heterogeneity (*R*^2^ = 100%) for the analysis of pleural VEGF levels in TPE compared with PPE with no residual heterogeneity (*p* = 0.7421). There was no association between the male percentage or mean age and the effect size for other analyses (S4 Table).

## Discussion

This is the first meta-analysis comparing VEGF levels between patients with TB and different control conditions, including healthy controls, patients with malignancy, patients with empyema and PPE, and patients with transudate effusion. We run meta-analyses, subgroup meta-analyses, and meta-regressions while considering different samples that could be used for VEGF measurement and other factors that might affect VEGF profile, e.g., geographic location, age, and gender. The main findings are as follows: (1) serum levels of VEGF are higher in patients with active TB compared with healthy controls without other respiratory diseases, including those with latent TB infection or uninfected individuals, whereas plasma levels of VEGF in patients with TB are not different from those of healthy controls; (2) both serum and pleural levels of VEGF are increased in patients with TPE compared with patients with transudate, CHF/CRF, or cirrhotic pleural effusion; (3) ascitic/pericardial fluid, serum, and pleural levels of VEGF are decreased in patients with TB compared with patients with malignancy; (4) pleural levels of VEGF are lower in patients with TPE compared with those with empyema and PPE, whereas serum levels of VEGF are not different between these patients; (5) both CSF and serum levels of VEGF are increased in patients with active TBM compared with controls, including patients with inactive TBM or non-TBM subjects; (6) post-ATT levels of VEGF are increased compared with pre-ATT levels of VEGF; and (7) the mean age and male percentage of the TB group explained large and total amount of heterogeneity for the meta-analysis of blood and pleural VEGF levels compared with healthy controls and patients with PPE, respectively, whereas these moderators did not show any significant interaction with the effect size for other analyses.

The potential role of VEGF in the pathophysiology of TB is derived from different pieces of evidence. Macrophages and their behavior crucially contribute to the determination of host responses against *M*. *tb* infection [57]. *In vitro* studies show that VEGF is among the angiogenic factors that *M*. *tb*-infected macrophages secrete to facilitate the spread of *M*. *tb* infection from the primary site of infection. e.g., the lung, to a different site [35]. In animal studies, anti-VEGF treatment decreases the spread of *M*. *tb* infection [35]. VEGF expression is also found in the brains of animals with TBM, while it was absent in the brains of vaccinated animals [58]. In addition, the observation of lung tissues obtained from patients with fibro-cavernous TB identified the VEGF-A-expressing macrophages distributed over the pyogenic and granulation layers, the areas of fibrosis, draining bronchus, distelectase, and emphysema, and the intact tissues [59]. These lines of evidence lead us to conclude that endothelial function might be altered in TB, and VEGF is crucially involved in TB-related changes in endothelial function.

Clinical studies support the potential use of VEGF as a biomarker of disease severity and activity. Compared with controls with inactive, old, or cured disease, patients with active TB reveal higher VEGF levels [6, 7, 60]. Also, smear-positive patients and culture-positive patients have higher VEGF levels than their smear-negative and culture-negative counterparts [61]. Among patients with pulmonary TB, the increase of systemic VEGF is more prominent in patients with cavitation [61, 62] and in those who have bilateral involvement [62]. For patients with TPE, pleural VEGF levels in patients with loculated pleural effusion are higher than those in patients with non-loculated pleural effusion, and that patients with higher pleural levels of VEGF at baseline appear to be more likely to develop residual pleural thickening (RPE) [63]. In addition, patients with drug-resistant TB with higher systemic levels of VEGF at baseline might be more prone to delays in sputum culture conversion [61].

The development of agents that target VEGF-mediated angiogenesis might help balance endothelial function and add to the value of standard ATT. Bevacizumab is an anti-VEGF drug used for different types of cancers and eye diseases. It has been shown to normalize the vascular dynamics of granuloma, an organized cellular structure that contains immune cells and is recognized as a hallmark of TB, making it more versatile to therapeutic medications [64]. Also, there have been reported cases with ocular TB who responded to anti-VEGF treatments [65, 66].

Although the main finding of higher blood VEGF levels in active TB compared with healthy controls is robust as the leave-one-out results revealed, the large amount of heterogeneity remains to be investigated. This meta-analysis is based on 16 observations (*N* = 1510), from which 13 (81.3%) reported higher VEGF levels in patients, one reported lower VEGF levels in patients, and two reported no difference between patients and control. First, we attempted to do separate latent TB infection and TB-uninfected subjects from a mixed healthy control group. However, it was not possible since most studies (62.5%) did not consider the diagnosis of latent TB infection in healthy controls [5, 7, 10, 17, 19, 27, 34, 35, 39, 41], while some studies addressed the issue and provided the related VEGF data [6, 13, 31, 52]. Second, we performed subgroup analyses by the geographic location, the blood fraction assessed, and the assay used. The effect sizes were significant for specific subgroups of studies (East Asia & Pacific, serum, and ELISA); however, these predictors could not explain the substantial heterogeneity. Finally, both the mean age and male percentage of the TB group were not significant moderators of the SMD and its variance.

Another main finding of our meta-analysis is that both serum and pleural VEGF levels in patients with TPE are increased compared with those with transudate, CHF/CRF, or cirrhotic effusion. CHF is one of the common etiologies of transudate effusion characterized by endothelial dysfunction. Studies suggest that VEGF levels in patients with CHF increase to maintain endothelial repair [67, 68]. Again, we could not separate CHF and other types of transudate effusion from the mixed group of transudate effusions. Overall, for blood VEGF levels (*k* = 7), four observations reported higher VEGF levels in patients with TPE [17, 21, 26, 33], and three found no difference between patients with TPE and patients with transudative, CHF/CFR, or cirrhosis effusion [22, 24, 41]. For pleural VEGF levels, 13 observations indicated higher VEGF levels in TPE [9, 15, 17, 21–24, 26, 33, 40, 41, 47, 48], while five found no difference [16, 20, 43, 44, 49]. There was moderate to high heterogeneity for pleural (*I*^2^ = 71.6%) and blood (*I*^2^ = 80.8%) VEGF levels. The leave-one-out results showed that both effect sizes are robust; however, the study by Fathi and colleagues and the study by Momi and colleagues were found to be accounted for greater than 10% of the true variance. Due to the small number of observations included in the meta-analysis of blood VEGF levels, we could not run subgroup analysis and meta-regression. For pleural VEGF levels, we found that higher VEGF levels in TPE are significant regardless of the geographic location; however, the heterogeneity was lower in Europe & Central Asia studies than those of the Middle East & North Africa (61.9% vs. 88.5%). Meta-regressions did not show any significant association between the effect size and tested moderators.

Our meta-analyses of VEGF levels in TB compared with malignancy were based on 25, 12, and 3 observations for pleural, blood, and ascitic/pericardial fluid samples. The SMD for all analyses was statistically large, calculated as -1.47, -1.30, and -1.54, respectively, and robust as the influence analysis results demonstrated. Several meta-analyses have been published considering the VEGF profile and its prognostic significance in different types of cancer [69–72]. These studies suggest the hyperactivated VEGF pathway as a target in cancer therapy [73]. Our meta-analyses confirm the previous meta-analyses that VEGF levels are increased in malignancy and add that the increase of VEGF in TB is significantly less than that in malignancy. There was high heterogeneity for both blood and pleural VEGF (*I*^2^ > 90%). We did subgroup analysis by geographic location and observed that lower blood and pleural VEGF levels in TB exist in specific subgroups of studies of East Asia & Pacific (for both blood and pleural levels) and the Middle East and North Africa (for pleural levels). However, again, there was high heterogeneity in these subgroups (*I*^2^ > 90%). Meta-regressions showed that the mean age of patients was accounted for more than 70% of the true variance among studies of blood VGEF levels. However, no such interaction with the effect size was found for the male percentage of patients.

Accordingly, the important limitation of the study is that we could not address the high heterogeneity among studies of blood VEGF in TB vs. healthy controls and of pleural VEGF in TB vs. malignancy. There might be unmeasured factors behind this heterogeneity that need to be explored in future research. Another limitation of the study was that we could not do within-group meta-analyses to compare VEGF levels between the TB subgroups, e.g., pulmonary vs. extrapulmonary TB, TB with cavitation vs. TB without cavitation, TB with RPT vs. TB without RPT, smear-positive TB vs. smear-negative TB, and HIV-infected TB vs. HIV-uninfected TV, since the number of observations was small. Again, for TBM, the most lethal form of TB, we could not investigate the association between the VEGF profile and the disease outcome, given the small number of observations with available data.

## Conclusion

This meta-analysis results suggest that blood, CSF, and pleural levels of VEGF are increased in patients with active TB, TPE, and TBM compared with healthy controls, patients with transudative effusions, and non-TBM controls. However, the TB-related increase in ascitic, blood, and pleural VEGF is lower than malignancy-related increase. This calls the need for the development of anti-VEGF treatments that specifically target the TB-related change in VEGF.

## Supporting information

S1 Checklist PRISMA checklist for meta-analysis of VEGF levels in tuberculosis. (DOCX)

S1 Fig Meta-analysis of VEGF levels in blood (top) and PE (bottom) for patients with TPE vs. patients with empyema and PPE.
(TIF)

S2 Fig Meta-analysis of CSF VEGF levels in patients with TBM vs. patients with VM. (TIF)

S3 Fig Meta-analysis of CSF vs. serum VEGF levels in patients with TBM. (TIF)

S4 Fig Meta-analysis of VEGF levels in ascites or pericardial fluid in patients with TB ascites vs. patients with malignant ascites. (TIF)

S5 Fig Meta-analysis of pleural VEGF levels in patients with TPE vs. patients with lung cancer. (TIF)

S6 Fig Funnel plot for the meta-analysis of blood VEGF levels in patients with TPE vs. patients with MPE using the trim and fill method. (TIF)

S7 Fig Funnel plot for the meta-analysis of pleural VEGF levels in patients with TPE vs. patients with empyema and PPE using the trim and fill method. (TIF)

S8 Fig Sensitivity analysis plot for the meta-analysis of blood VEGF levels in patients with active TB vs. healthy controls including individuals with latent TB infection and TB-uninfected individuals. (TIF)

S9 Fig Sensitivity analysis plot for the meta-analysis of blood VEGF levels in patients with TPE vs. patients with MPE. (TIF)

S10 Fig Sensitivity analysis plot for the meta-analysis of pleural VEGF levels in patients with TPE vs. patients with lung cancer. (TIF)

S11 Fig Sensitivity analysis plot for the meta-analysis of pleural VEGF levels in patients with TPE vs. patients with MPE. (TIF)

S12 Fig Sensitivity analysis plot for the meta-analysis of pleural VEGF levels in patients with TPE vs. patients with transudate, CHF/CRF, or cirrhotic effusion. (TIF)

S13 Fig Sensitivity analysis plot for the meta-analysis of pleural vs. blood VEGF levels in patients with TPE. (TIF)

S14 Fig Sensitivity analysis plot for the meta-analysis of pleural VEGF levels in patients with TPE vs. patients with empyema and PPE. (TIF)

S15 Fig Subgroup meta-analysis of pleural vs. serum VEGF levels in patients with TPE by geographic location. (TIF)

S16 Fig Subgroup meta-analysis of blood VEGF levels in patients with active TB vs. healthy controls including individuals with latent TB infection and TB-uninfected individuals by geographic location. (TIF)

S17 Fig Subgroup meta-analysis of blood VEGF levels in patients with active TB vs. healthy controls including individuals with latent TB infection and TB-uninfected individuals by blood fraction. (TIF)

S18 Fig Subgroup meta-analysis of blood VEGF levels in patients with active TB vs. healthy controls including individuals with latent TB infection and TB-uninfected individuals by the assay. (TIF)

S19 Fig Subgroup meta-analysis of blood VEGF levels in patients with TPE vs. patients with MPE by geographic location. (TIF)

S20 Fig Subgroup meta-analysis of pleural VEGF levels in patients with TPE vs. patients with MPE by geographic location. (TIF)

S21 Fig Subgroup meta-analysis of pleural VEGF levels in patients with TPE vs. patients with empyema and PPE by geographic location. (TIF)

S22 Fig Subgroup meta-analysis of pleural VEGF levels in patients with TPE vs. patients with transudate, CHF/CRF, or cirrhotic effusion by geographic location. (TIF)

S1 Table Characteristics of studies included in the meta-analysis of VEGF. (DOCX)

S2 Table The quality of studies included in the meta-analysis of VEGF ranked using the Newcastle-Ottawa Scale. (DOCX)

S3 Table Subgroup meta-analysis results summary. (DOCX)

S4 Table Meta-regression results summary. (DOCX)

S1 Text The reason of exclusion of studies during the detailed review. (DOCX)

## References

[1] Mukae H, Ashitani Ji, Tokojima M, Ihi T, Kohno S, Matsukura S (2003) Elevated levels of circulating adhesion molecules in patients with active pulmonary tuberculosis. Respirology (Carlton, Vic) 8 (3):326–331 doi: 10.1046/j.1440-1843.2003.00471.x 12911826

[2] Ferrara N, Gerber H-P, LeCouter J (2003) The biology of VEGF and its receptors. Nature medicine 9 (6):669–676 doi: 10.1038/nm0603-669 12778165

[3] Moher D, Liberati A, Tetzlaff J, Altman DG, Prisma G (2009) Preferred reporting items for systematic reviews and meta-analyses: the PRISMA statement. PLoS medicine 6 (7):e1000097 doi: 10.1371/journal.pmed.1000097 19621072 PMC2707599

[4] Wells GA, Shea B, O’Connell D, Peterson J, Welch V, Losos M et al. (2000) The Newcastle-Ottawa Scale (NOS) for assessing the quality of nonrandomised studies in meta-analyses. Oxford,

[5] Abe Y, Nakamura M, Oshika Y, Hatanaka H, Tokunaga T, Ohkubo Y et al. (2001) Serum levels of vascular endothelial growth factor and cavity formation in active pulmonary tuberculosis. Respiration; international review of thoracic diseases 68 (5):496–500. doi: 10.1159/000050557 11694812

[6] Ahmad R, Xie L, Pyle M, Suarez MF, Broger T, Steinberg D et al. (2019) A rapid triage test for active pulmonary tuberculosis in adult patients with persistent cough. Science translational medicine 11 (515). doi: 10.1126/scitranslmed.aaw8287 31645455

[7] Alatas F, Alatas O, Metintas M, Ozarslan A, Erginel S, Yildirim H (2004) Vascular endothelial growth factor levels in active pulmonary tuberculosis. Chest 125 (6):2156–2159. doi: 10.1378/chest.125.6.2156 15189936

[8] Antonangelo L, Vargas FS, Puka J, Seiscento M, Acencio MM, Teixeira LR et al. (2012) Pleural tuberculosis: is radiological evidence of pulmonary-associated disease related to the exacerbation of the inflammatory response? Clinics (Sao Paulo, Brazil) 67 (11):1259–1263. doi: 10.6061/clinics/2012(11)06 23184200 PMC3488982

[9] Bayram N, Karakan Y, Uyar M, Ozyurt B, Filiz A (2018) Vascular endothelial growth factor in pleural effusions and correlation with radiologic and biochemical parameters. Nigerian journal of clinical practice 21 (1):59–62. doi: 10.4103/njcp.njcp_370_16 29411725

[10] Bhat H, Ambekar JG, Harwalkar AK, Dongre N, Das KK (2019) Serum VEGF and TNF-α correlate bacterial burden in pulmonary tuberculosis. Indian Journal of Public Health Research and Development 10 (1):189–194. doi: 10.5958/0976-5506.2019.00039.1

[11] Chen Y-f, Tang Y-x, Jiang S-f (2003) The Significance of Detecting Vascular Endothelial Growth Factor in Differentiating Tuberculosis Pleural Effusion from Malignant Pleural Effusions. The Practical Journal of Cancer:03

[12] Daniil ZD, Zintzaras E, Kiropoulos T, Papaioannou AI, Koutsokera A, Kastanis A et al. (2007) Discrimination of exudative pleural effusions based on multiple biological parameters. The European respiratory journal 30 (5):957–964. doi: 10.1183/09031936.00126306 17690119

[13] Djoba Siawaya JF, Chegou NN, van den Heuvel MM, Diacon AH, Beyers N, van Helden P et al. (2009) Differential cytokine/chemokines and KL-6 profiles in patients with different forms of tuberculosis. Cytokine 47 (2):132–136. doi: 10.1016/j.cyto.2009.05.016 19570688

[14] Dong WG, Sun XM, Yu BP, Luo HS, Yu JP (2003) Role of VEGF and CD44v6 in differentiating benign from malignant ascites. World journal of gastroenterology 9 (11):2596–2600. doi: 10.3748/wjg.v9.i11.2596 14606105 PMC4656549

[15] Fathy M, Al Ansary M, Zakaria M, Abdel-Hafiz H, Said M (2014) Role of vascular endothelial growth factor (VEGF) in diagnosis of pleural effusion of different origins. Egyptian Journal of Chest Diseases and Tuberculosis 63 (3):611–615

[16] Fricke S, Hoheisel G, Gessner C, Bauer K, Seyfarth HJ, Hammerschmidt S et al. (2014) Mediators in pleural effusions of different origin: a two-step diagnostic study. Laboratoriumsmedizin-Journal of Laboratory Medicine 38 (3):121–127. doi: 10.1515/labmed-2014-0006

[17] Hamed EA, El-Noweihi AM, Mohamed AZ, Mahmoud A (2004) Vasoactive mediators (VEGF and TNF-alpha) in patients with malignant and tuberculous pleural effusions. Respirology (Carlton, Vic) 9 (1):81–86. doi: 10.1111/j.1440-1843.2003.00529.x 14982607

[18] Husain N, Awasthi S, Haris M, Gupta RK, Husain M (2008) Vascular endothelial growth factor as a marker of disease activity in neurotuberculosis. The Journal of infection 56 (2):114–119. doi: 10.1016/j.jinf.2007.11.004 18158186

[19] Im BK, Oh YJ, Sheen SS, Lee KS, Park KJ, Hwang SC et al. (2016) Clinical Significance of Vascular Endothelial Growth Factor in Patients with Lung Cancer and Tuberculous Pleurisy. Tuberculosis and Respiratory Diseases 50 (2):171–181

[20] Jankowska R, Porebska I, Dyła T (2002) [Evaluation of vascular endothelial growth factor (VEGF) in neoplastic and tuberculosis effusions—preliminary results]. Pneumonologia i alergologia polska 70 (5–6):258–264 12518624

[21] Jin HY, Lee KS, Jin SM, Lee YC (2004) Vascular endothelial growth factor correlates with matrix metalloproteinase-9 in the pleural effusion. Respiratory medicine 98 (2):115–122. doi: 10.1016/j.rmed.2003.09.002 14971873

[22] Kalomenidis I, Kollintza A, Sigala I, Papapetropoulos A, Papiris S, Light RW et al. (2006) Angiopoietin-2 levels are elevated in exudatative pleural effusions. Chest 129 (5):1259–1266. doi: 10.1378/chest.129.5.1259 16685017

[23] Kaya A, Poyraz B, Celik G, Ciledag A, Gulbay BE, Savas H et al. (2005) [Vascular endothelial growth factor in benign and malignant pleural effusions]. Archivos de bronconeumologia 41 (7):376–379. doi: 10.1016/s1579-2129(06)60244-9 16029730

[24] Khalil NH, Abdelaal DE (2017) Vascular endothelial growth factor in diagnosis of pleural effusion. Egyptian Journal of Chest Diseases and Tuberculosis 66 (1):115–119. doi: 10.1016/j.ejcdt.2016.12.006

[25] Kim HR, Kim BR, Park RK, Yoon KH, Jeong ET, Hwang KE (2017) Diagnostic Significance of Measuring Vascular Endothelial Growth Factor for the Differentiation between Malignant and Tuberculous Pleural Effusion. The Tohoku journal of experimental medicine 242 (2):137–142. doi: 10.1620/tjem.242.137 28626164

[26] Kiropoulos TS, Kostikas K, Gourgoulianis KI, Alatas F, Alatas O, Metintas M et al. (2005) Vascular endothelial growth factor levels in pleural fluid and serum of patients with tuberculous pleural effusions [6] (multiple letters). Chest 128 (1):468–469. doi: 10.1378/chest.128.1.468 16002978

[27] Li ZY, Li CL, Bao RR, Liu ZD (2020) Expressions of miR-29a, TNF-A and Vascular Endothelial Growth Factor in Peripheral Blood of Pulmonary Tuberculosis Patients and Their Clinical Significance. Iranian journal of public health 49 (9):1683–1691 doi: 10.18502/ijph.v49i9.4085 33643943 PMC7898107

[28] Lim SC, Jung SI, Kim YC, Park KO (2000) Vascular endothelial growth factor in malignant and tuberculous pleural effusions. Journal of Korean medical science 15 (3):279–283. doi: 10.3346/jkms.2000.15.3.279 10895968 PMC3054640

[29] Liu J, Zeng Y, Ma W, Chen S, Zheng Y, Ye S et al. (2010) Preliminary investigation of the clinical value of vascular endothelial growth factor and hypoxia-inducible factor-1alpha in pericardial fluid in diagnosing malignant and tuberculous pericardial effusion. Cardiology 116 (1):37–41. doi: 10.1159/000313465 20424452

[30] Matsuyama W, Hashiguchi T, Umehara F, Matsuura E, Kawabata M, Arimura K et al. (2001) Expression of vascular endothelial growth factor in tuberculous meningitis. Journal of the neurological sciences 186 (1–2):75–79. doi: 10.1016/s0022-510x(01)00515-9 11412875

[31] Mihret A, Bekele Y, Bobosha K, Kidd M, Aseffa A, Howe R et al. (2013) Plasma cytokines and chemokines differentiate between active disease and non-active tuberculosis infection. The Journal of infection 66 (4):357–365. doi: 10.1016/j.jinf.2012.11.005 23178506

[32] Misra UK, Kalita J, Singh AP, Prasad S (2013) Vascular endothelial growth factor in tuberculous meningitis. The International journal of neuroscience 123 (2):128–132. doi: 10.3109/00207454.2012.743127 23098361

[33] Momi H, Matsuyama W, Inoue K, Kawabata M, Arimura K, Fukunaga H et al. (2002) Vascular endothelial growth factor and proinflammatory cytokines in pleural effusions. Respiratory medicine 96 (10):817–822. doi: 10.1053/rmed.2002.1364 12412982

[34] Omar M, ElAdl T, Abdullah S, Hamza H, ElAdl T, Neamatallah M (2013) Clinical Implications for Vascular Endothelial Growth Factor Levels among Egyptians with Pulmonary Tuberculosis. Life Science Journal-Acta Zhengzhou University Overseas Edition 10 (1):2978–2983

[35] Polena H, Boudou F, Tilleul S, Dubois-Colas N, Lecointe C, Rakotosamimanana N et al. (2016) Mycobacterium tuberculosis exploits the formation of new blood vessels for its dissemination. Scientific reports 6:33162. doi: 10.1038/srep33162 27616470 PMC5018821

[36] Qama D, Choi WI, Kwon KY (2012) Immune responses in the lungs of patients with tuberculous pleural effusion without pulmonary tuberculosis. BMC immunology 13:45. doi: 10.1186/1471-2172-13-45 22889060 PMC3460733

[37] Qian Q, Zhan P, Sun WK, Zhang Y, Song Y, Yu LK (2012) Vascular endothelial growth factor and soluble intercellular adhesion molecule-1 in lung adenocarcinoma with malignant pleural effusion: correlations with patient survival and pleural effusion control. Neoplasma 59 (4):433–439. doi: 10.4149/neo_2012_056 22489699

[38] Qiu B, Li G, Fan X, Xing X (2009) Diagnostic value of vascular endothelial growth factor and adenosine deaminase detection in differentiating tuberculous meningitis from viral meningitis. Medical Journal of Wuhan University 30 (6):806–809

[39] Ranaivomanana P, Raberahona M, Rabarioelina S, Borella Y, Machado A, Randria MJD et al. (2018) Cytokine Biomarkers Associated with Human Extra-Pulmonary Tuberculosis Clinical Strains and Symptoms. Frontiers in microbiology 9:275. doi: 10.3389/fmicb.2018.00275 29515555 PMC5826350

[40] Ruiz E, Alemán C, Alegre J, Monasterio J, Segura RM, Armadans L et al. (2005) Angiogenic factors and angiogenesis inhibitors in exudative pleural effusions. Lung 183 (3):185–195. doi: 10.1007/s00408-004-2533-0 16078040

[41] Sack U, Hoffmann M, Zhao XJ, Chan KS, Hui DS, Gosse H et al. (2005) Vascular endothelial growth factor in pleural effusions of different origin. The European respiratory journal 25 (4):600–604. doi: 10.1183/09031936.05.00037004 15802331

[42] Safe IP, Amaral EP, Araújo-Pereira M, Lacerda MVG, Printes VS, Souza AB et al. (2021) Adjunct N-Acetylcysteine Treatment in Hospitalized Patients With HIV-Associated Tuberculosis Dampens the Oxidative Stress in Peripheral Blood: Results From the RIPENACTB Study Trial. Frontiers in immunology 11. doi: 10.3389/fimmu.2020.602589 33613521 PMC7889506

[43] Saraya T, Ohkuma K, Watanabe T, Mikura S, Kobayashi F, Aso J et al. (2018) Diagnostic Value of Vascular Endothelial Growth Factor, Transforming Growth Factor-beta, Interleukin-8, and the Ratio of Lactate Dehydrogenase to Adenosine Deaminase in Pleural Effusion. Lung 196 (2):249–254. doi: 10.1007/s00408-018-0090-1 29353318

[44] Seiscento M, Vargas FS, Acencio MM, Teixeira LR, Capelozzi VL, Sales RK et al. (2010) Pleural fluid cytokines correlate with tissue inflammatory expression in tuberculosis. The international journal of tuberculosis and lung disease: the official journal of the International Union against Tuberculosis and Lung Disease 14 (9):1153–1158 20819261

[45] Shen H, Feng GZ, Cui J, Du Q, Qin Y, Cai JK et al. (2015) Clinical implications of serum hypoxia inducible factor-1 alpha and vascular endothelial growth factor in lung cancer. Tumori 101 (4):404–411. doi: 10.5301/tj.5000320 25983091

[46] Tai M, Tan HY, Yong YK, Shankar EM, Viswanathan S, Nor HM et al. (2017) Role of cytokines in the assessment of clinical outcome and neuroimaging findings in patients with tuberculous meningitis. Neurology Asia 22 (3):209–220

[47] Tas D, Okutan O, Caliskan T, Ipcioglu OM, Ciftci F, Kartaloglu Z (2009) Vascular Endothelial Growth Factor for Differential Diagonsis of Nonmalignant Pleural Effusions. Nobel Medicus 5 (3):40–44

[48] Teixeira LR, Dias MB, Sales RK, Antonangelo L, Alvarenga VA, Puka J et al. (2016) Profile of Metalloproteinases and Their Association with Inflammatory Markers in Pleural Effusions. Lung 194 (6):1021–1027. doi: 10.1007/s00408-016-9945-5 27677622

[49] Tomimoto H, Yano S, Muguruma H, Kakiuchi S, Sone S (2007) Levels of soluble vascular endothelial growth factor receptor 1 are elevated in the exudative pleural effusions. The journal of medical investigation: JMI 54 (1–2):146–153. doi: 10.2152/jmi.54.146 17380026

[50] van der Flier M, Hoppenreijs S, van Rensburg AJ, Ruyken M, Kolk AH, Springer P et al. (2004) Vascular endothelial growth factor and blood-brain barrier disruption in tuberculous meningitis. The Pediatric infectious disease journal 23 (7):608–613. doi: 10.1097/01.inf.0000131634.57368.45 15247597

[51] Visser DH, Solomons RS, Ronacher K, van Well GT, Heymans MW, Walzl G et al. (2015) Host immune response to tuberculous meningitis. Clinical infectious diseases: an official publication of the Infectious Diseases Society of America 60 (2):177–187. doi: 10.1093/cid/ciu781 25301213

[52] Wang S, Li Y, Shen Y, Wu J, Gao Y, Zhang S et al. (2018) Screening and identification of a six-cytokine biosignature for detecting TB infection and discriminating active from latent TB. Journal of translational medicine 16 (1):206. doi: 10.1186/s12967-018-1572-x 30029650 PMC6054748

[53] Xue K, Xiong S, Xiong W (2007) Clinical value of vascular endothelial growth factor combined with interferon-gamma in diagnosing malignant pleural effusion and tuberculous pleural effusion. Journal of Huazhong University of Science and Technology Medical sciences = Hua zhong ke ji da xue xue bao Yi xue Ying De wen ban = Huazhong keji daxue xuebao Yixue Yingdewen ban 27 (5):495–497. doi: 10.1007/s11596-007-0504-4 18060618

[54] Zhan N, Dong W-G, Wang J (2016) The clinical significance of vascular endothelial growth factor in malignant ascites. Tumor Biology 37 (3):3719–3725 doi: 10.1007/s13277-015-4198-0 26462841

[55] Zhang Y, Yu LK, Lu GJ, Xia N, Xie HY, Hu W et al. (2014) Prognostic values of VEGF and endostatin with malignant pleural effusions in patients with lung cancer. Asian Pacific journal of cancer prevention: APJCP 15 (19):8435–8440. doi: 10.7314/apjcp.2014.15.19.8435 25339042

[56] Zhou WB, Bai M, Jin Y (2009) Diagnostic value of vascular endothelial growth factor and endostatin in malignant pleural effusions. The international journal of tuberculosis and lung disease: the official journal of the International Union against Tuberculosis and Lung Disease 13 (3):381–386 19275801

[57] Guirado E, Schlesinger LS, Kaplan G Macrophages in tuberculosis: friend or foe. In, 2013. Springer, pp 563–583 doi: 10.1007/s00281-013-0388-2 PMC3763202 23864058

[58] Zucchi FCR, Tsanaclis AMC, Moura-Dias Q, Silva CL, Pelegrini-da-Silva A, Neder L et al. (2013) Modulation of angiogenic factor VEGF by DNA-hsp65 vaccination in a murine CNS tuberculosis model. Tuberculosis 93 (3):373–380. doi: 10.1016/j.tube.2013.02.002 23491717

[59] Golubinskaya EP, Filonenko TG, Kramar TV, Yermola YA, Kubyshkin AV, Gerashenko AV et al. (2019) Dysregulation of VEGF-dependent angiogenesis in cavernous lung tuberculosis. Pathophysiology: the official journal of the International Society for Pathophysiology 26 (3–4):381–387 doi: 10.1016/j.pathophys.2019.11.004 31791834

[60] Matsuyama W, Hashiguchi T, Matsumuro K, Iwami F, Hirotsu Y, Kawabata M et al. (2000) Increased serum level of vascular endothelial growth factor in pulmonary tuberculosis. American journal of respiratory and critical care medicine 162 (3 Pt 1):1120–1122. doi: 10.1164/ajrccm.162.3.9911010 10988140

[61] Ferrian S, Manca C, Lubbe S, Conradie F, Ismail N, Kaplan G et al. (2017) A combination of baseline plasma immune markers can predict therapeutic response in multidrug resistant tuberculosis. PloS one 12 (5):e0176660. doi: 10.1371/journal.pone.0176660 28464011 PMC5413057

[62] Kumar NP, Banurekha VV, Nair D, Babu S (2016) Circulating Angiogenic Factors as Biomarkers of Disease Severity and Bacterial Burden in Pulmonary Tuberculosis. PloS one 11 (1):e0146318. doi: 10.1371/journal.pone.0146318 26727122 PMC4699686

[63] Bien MY, Wu MP, Chen WL, Chung CL (2015) VEGF correlates with inflammation and fibrosis in tuberculous pleural effusion. TheScientificWorldJournal 2015:417124. doi: 10.1155/2015/417124 25884029 PMC4391609

[64] Datta M, Via LE, Kamoun WS, Liu C, Chen W, Seano G et al. (2015) Anti-vascular endothelial growth factor treatment normalizes tuberculosis granuloma vasculature and improves small molecule delivery. Proceedings of the National Academy of Sciences 112 (6):1827–1832 doi: 10.1073/pnas.1424563112 25624495 PMC4330784

[65] Invernizzi A, Franzetti F, Viola F, Meroni L, Staurenghi G (2015) Optic nerve head tubercular granuloma successfully treated with anti-VEGF intravitreal injections in addition to systemic therapy. European journal of ophthalmology 25 (3):270–272 doi: 10.5301/ejo.5000528 25363855

[66] Taguchi M, Sakurai Y, Kanda T, Takeuchi M (2017) Anti-VEGF therapy for central retinal vein occlusion caused by tuberculosis-associated uveitis: a case report. International medical case reports journal 10:139 doi: 10.2147/IMCRJ.S128885 28458584 PMC5403006

[67] Vila V, Martínez-Sales V, Almenar L, Lázaro IS, Villa P, Reganon E (2008) Inflammation, endothelial dysfunction and angiogenesis markers in chronic heart failure patients. International journal of cardiology 130 (2):276–277 doi: 10.1016/j.ijcard.2007.07.010 17727986

[68] Chong AY, Caine GJ, Freestone B, Blann AD, Lip GYH (2004) Plasma angiopoietin-1, angiopoietin-2, and angiopoietin receptor tie-2 levels in congestive heart failure. Journal of the American College of Cardiology 43 (3):423–428 doi: 10.1016/j.jacc.2003.08.042 15013125

[69] Des Guetz G, Uzzan B, Nicolas P, Cucherat M, Morere JF, Benamouzig R et al. (2006) Microvessel density and VEGF expression are prognostic factors in colorectal cancer. Meta-analysis of the literature. British journal of cancer 94 (12):1823–1832 doi: 10.1038/sj.bjc.6603176 16773076 PMC2361355

[70] Delmotte P, Martin B, Paesmans M, Berghmans T, Mascaux C, Meert AP et al. (2002) VEGF and survival of patients with lung cancer: a systematic literature review and meta-analysis. Revue des maladies respiratoires 19 (5 Pt 1):577–584 12473944

[71] Zhao S-F, Yang X-D, Lu M-X, Sun G-W, Wang Y-X, Zhang Y-K et al. (2013) Prognostic significance of VEGF immunohistochemical expression in oral cancer: A meta-analysis of the literature. Tumor Biology 34 (5):3165–3171 doi: 10.1007/s13277-013-0886-9 23737289

[72] Kut C, Mac Gabhann F, Popel AS (2007) Where is VEGF in the body? A meta-analysis of VEGF distribution in cancer. British journal of cancer 97 (7):978–985 doi: 10.1038/sj.bjc.6603923 17912242 PMC2360423

[73] Alevizakos M, Kaltsas S, Syrigos KN (2013) The VEGF pathway in lung cancer. Cancer chemotherapy and pharmacology 72 (6):1169–1181 doi: 10.1007/s00280-013-2298-3 24085262

