# Supplementary figures and images for: Vascular endothelial growth factor levels in tuberculosis: A systematic review and meta-analysis
Source: PLoS One. 2022 May 25;17(5):e0268543. doi: 10.1371/journal.pone.0268543 (PMC9132289; doi:10.1371/journal.pone.0268543)

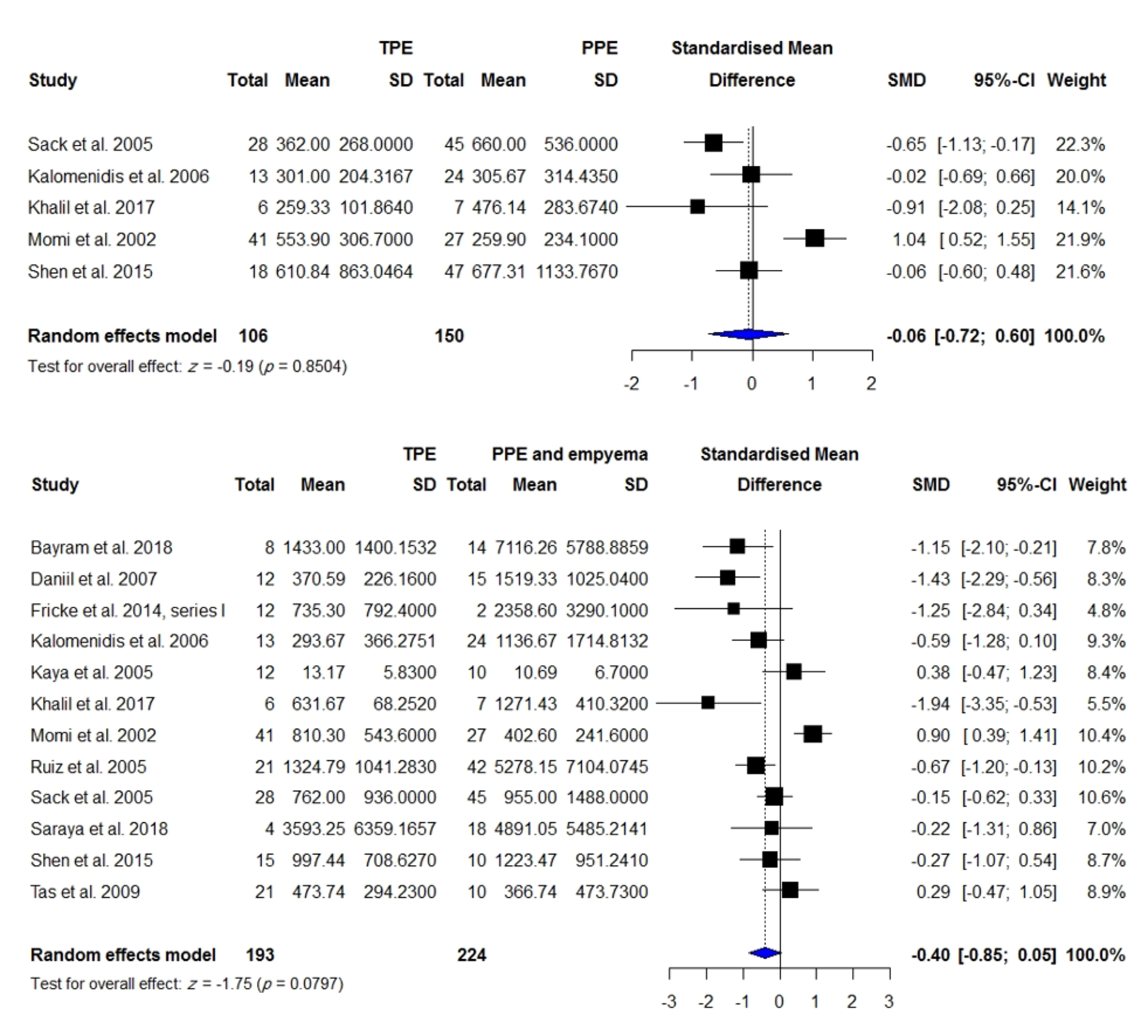

Supplement: S1 Fig — Meta-analysis of VEGF levels in blood (top) and PE (bottom) for patients with TPE vs. patients with empyema and PPE. (TIF) [file pone.0268543.s002.tif]

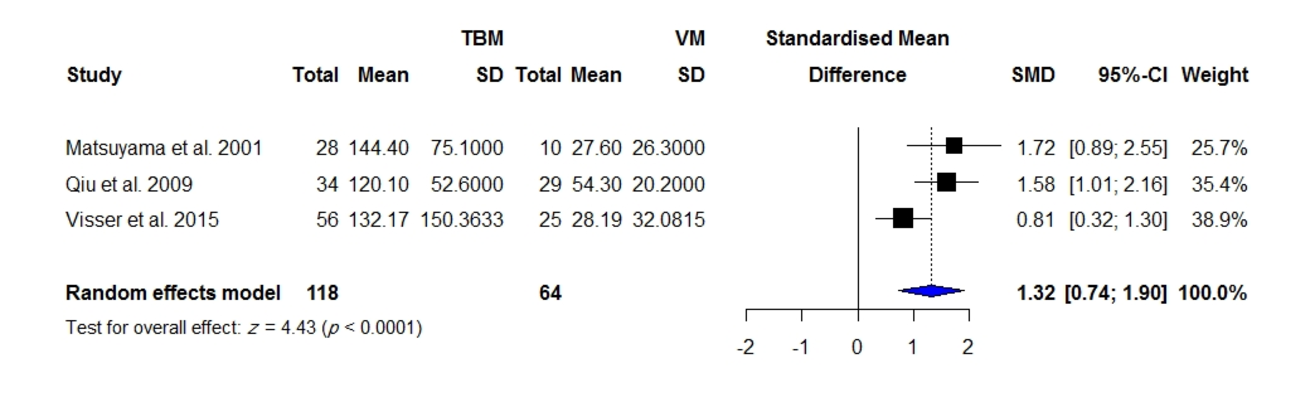

Supplement: S2 Fig — (TIF) [file pone.0268543.s003.tif]

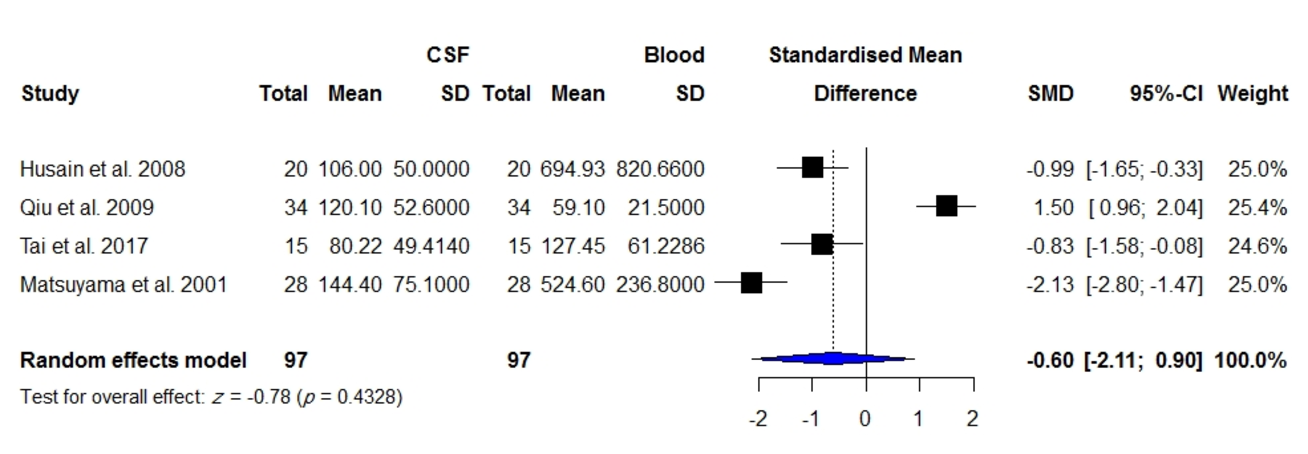

Supplement: S3 Fig — (TIF) [file pone.0268543.s004.tif]

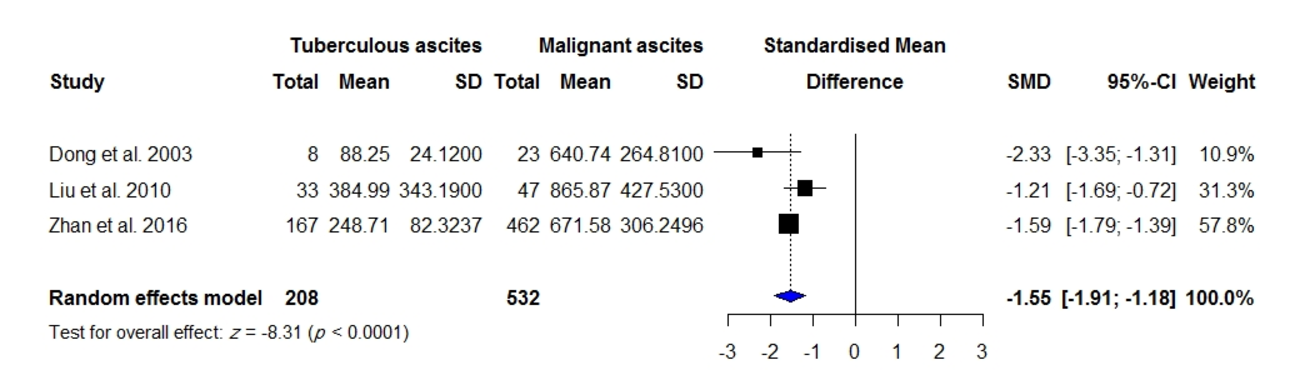

Supplement: S4 Fig — (TIF) [file pone.0268543.s005.tif]

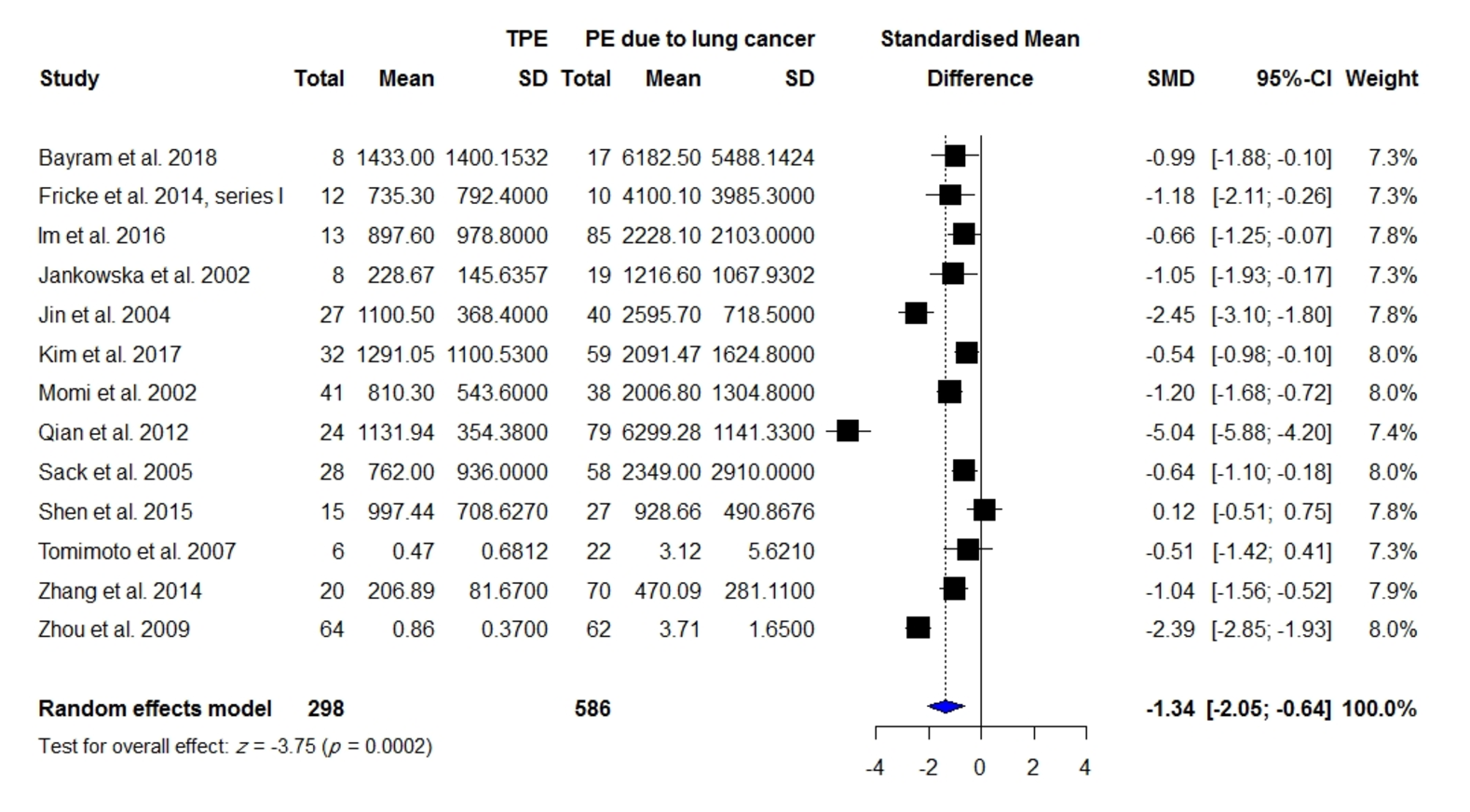

Supplement: S5 Fig — (TIF) [file pone.0268543.s006.tif]

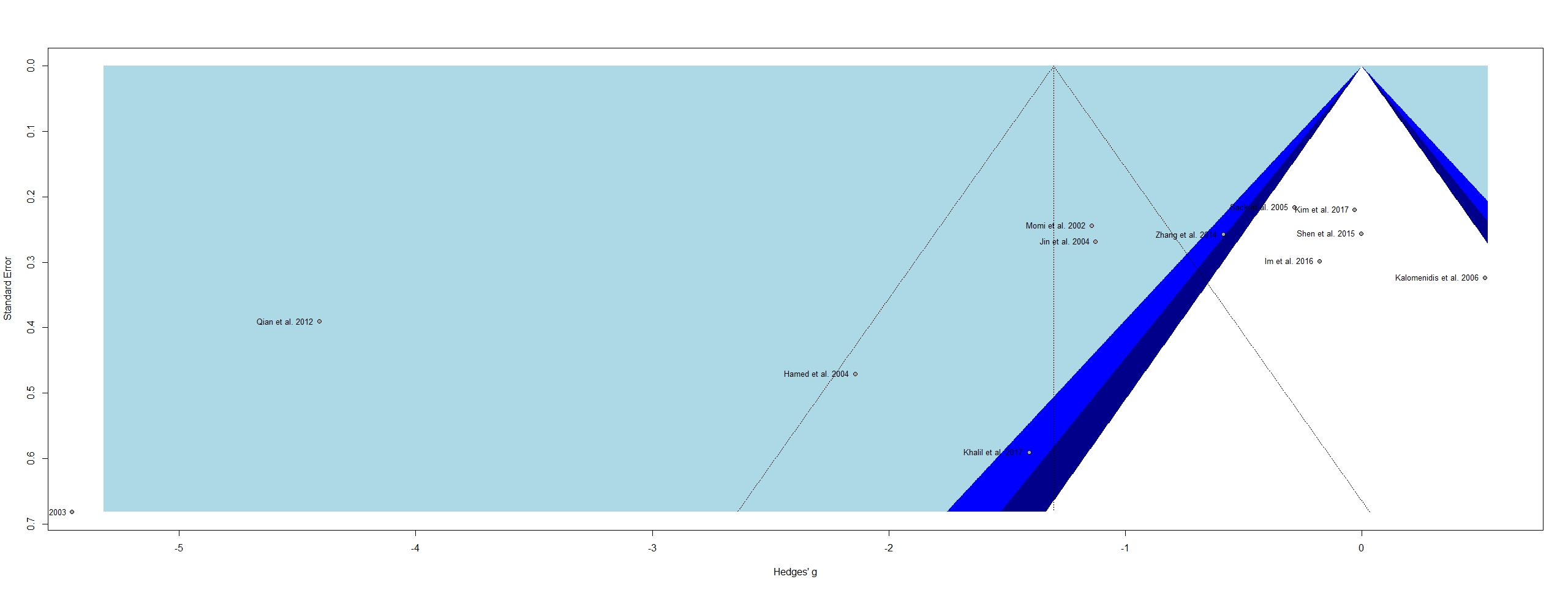

Supplement: S6 Fig — (TIF) [file pone.0268543.s007.tif]

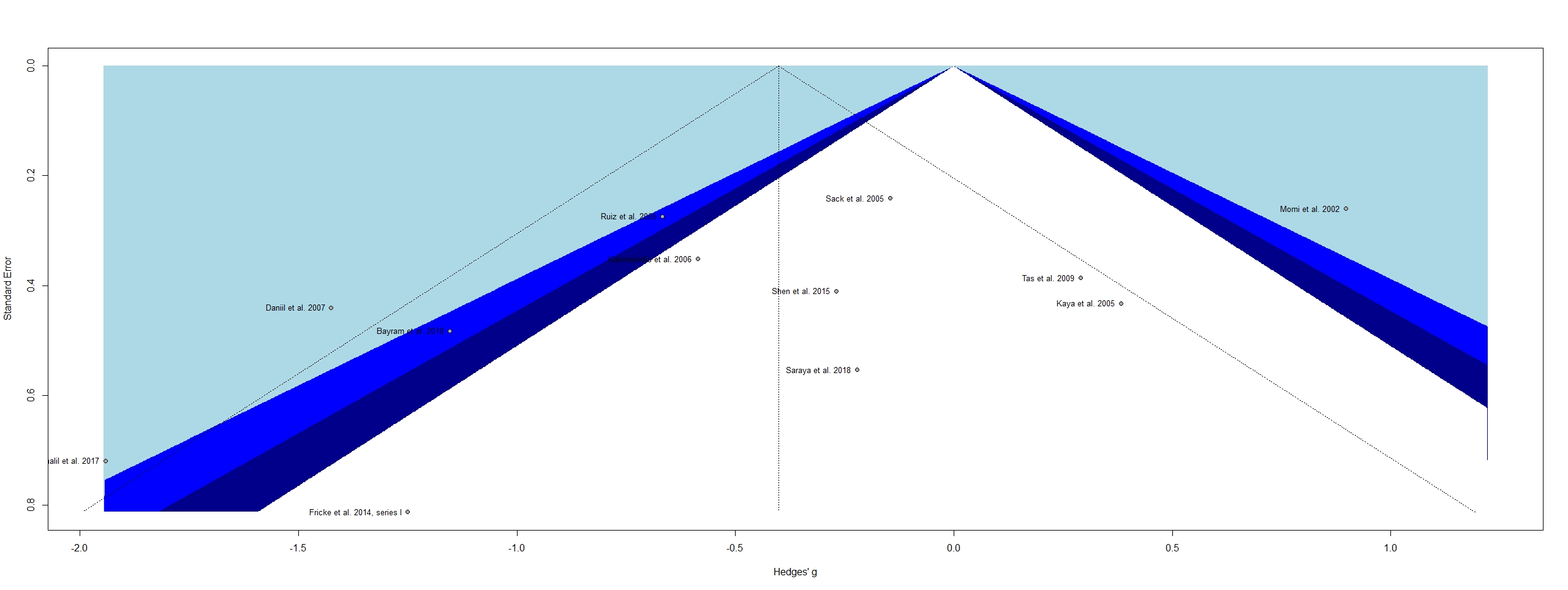

Supplement: S7 Fig — (TIF) [file pone.0268543.s008.tif]

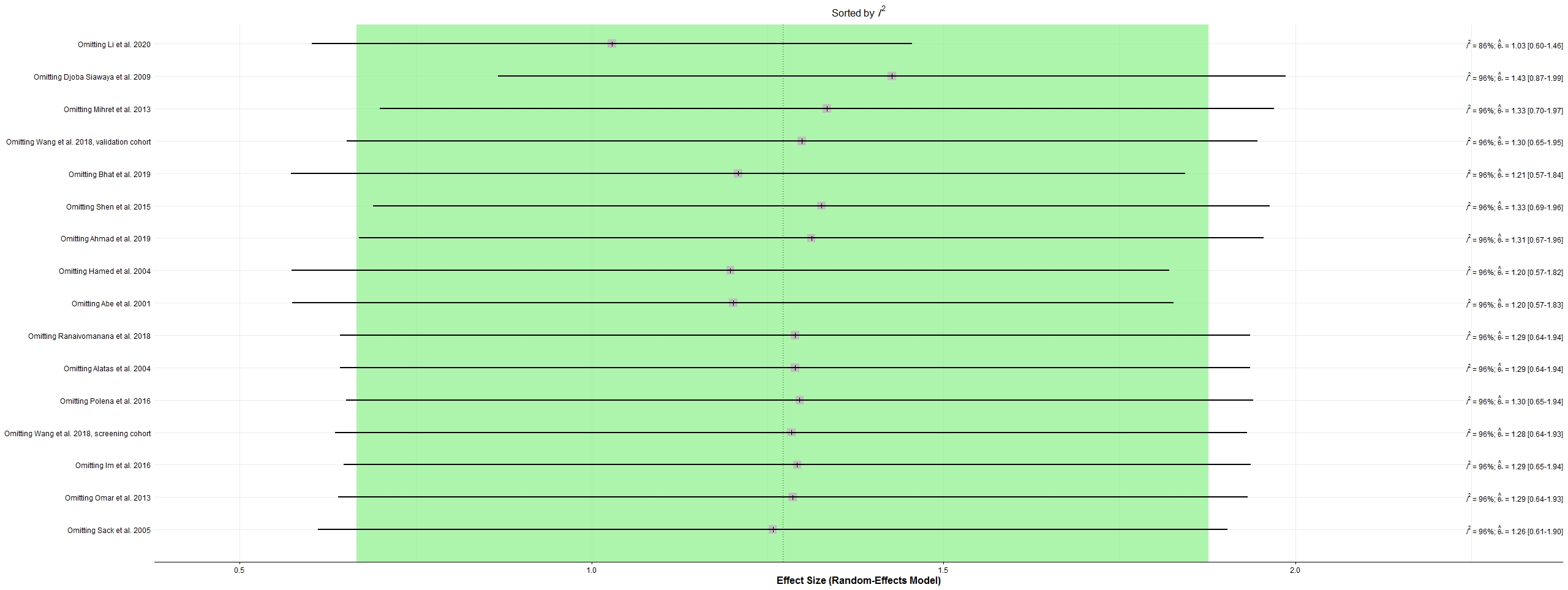

Supplement: S8 Fig — (TIF) [file pone.0268543.s009.tif]

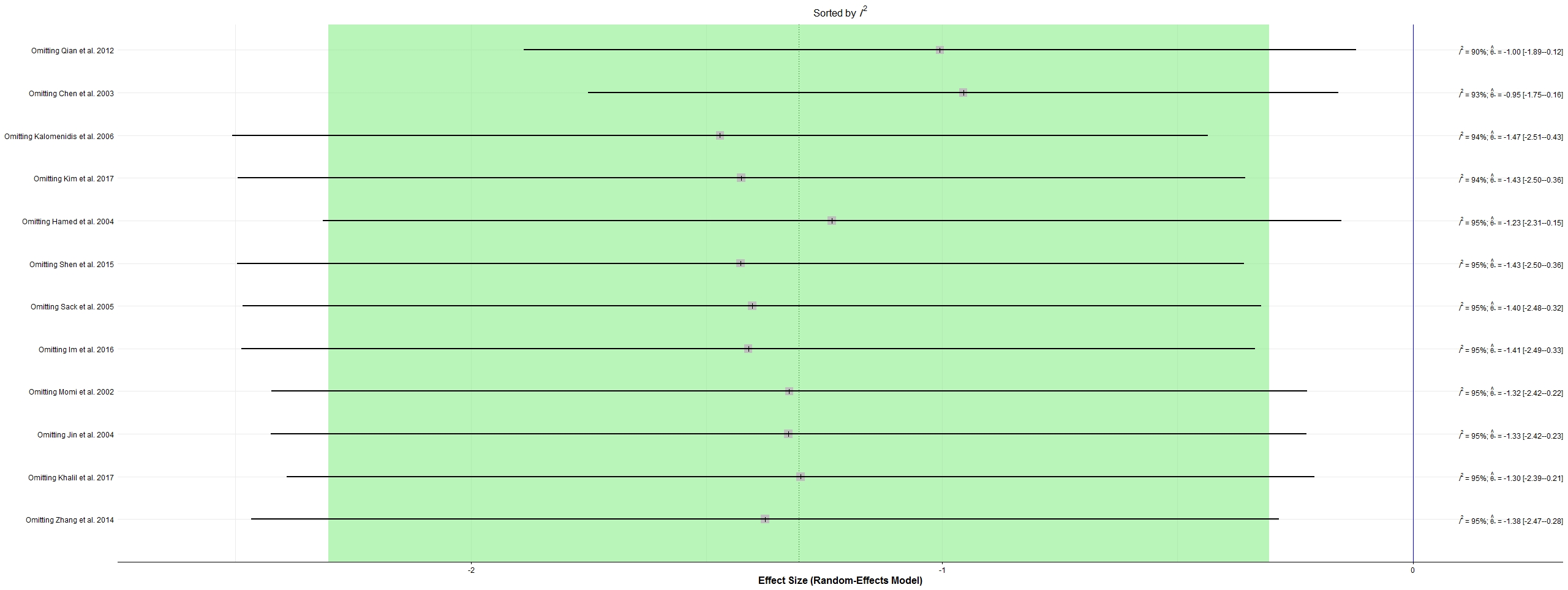

Supplement: S9 Fig — (TIF) [file pone.0268543.s010.tif]

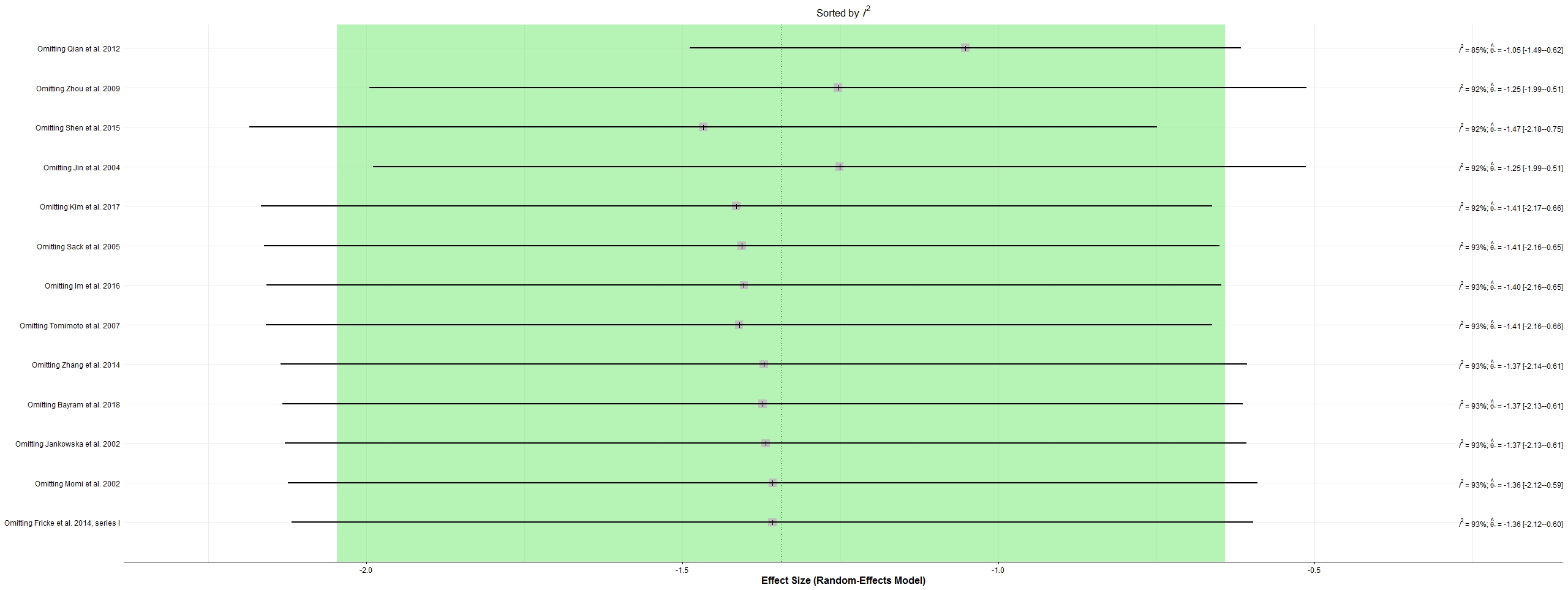

Supplement: S10 Fig — (TIF) [file pone.0268543.s011.tif]

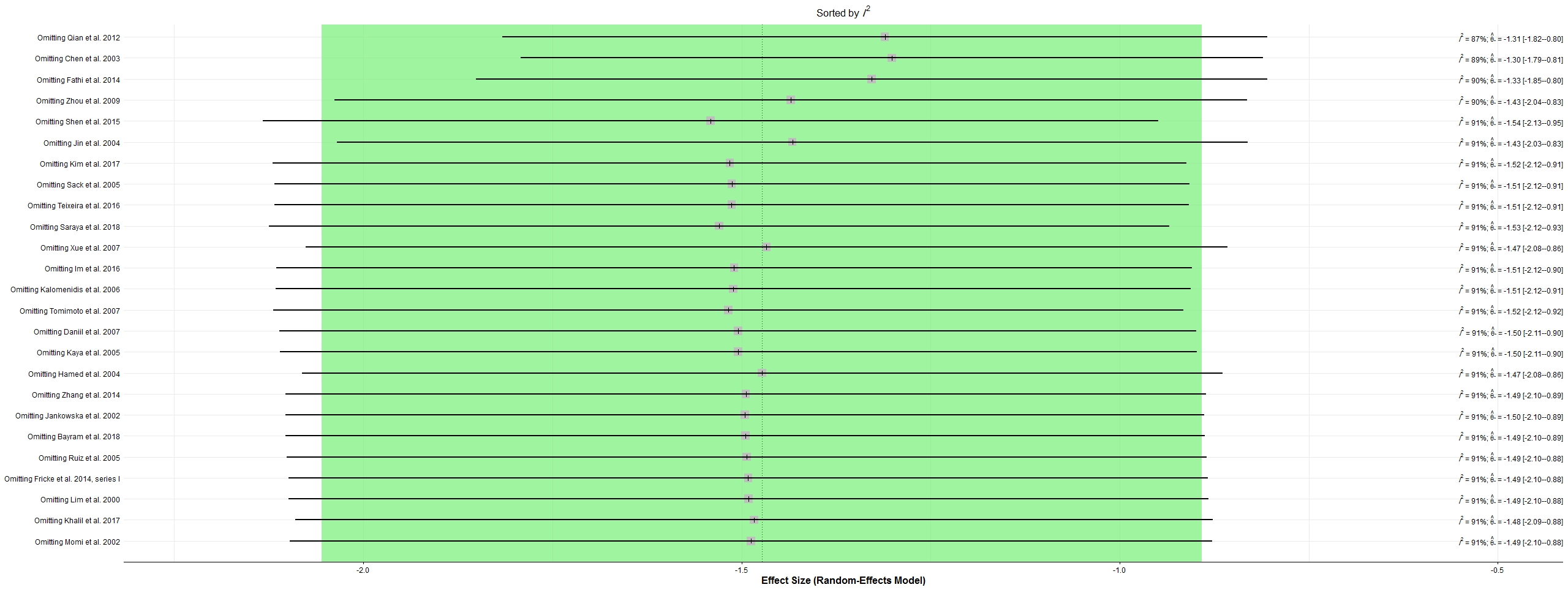

Supplement: S11 Fig — (TIF) [file pone.0268543.s012.tif]

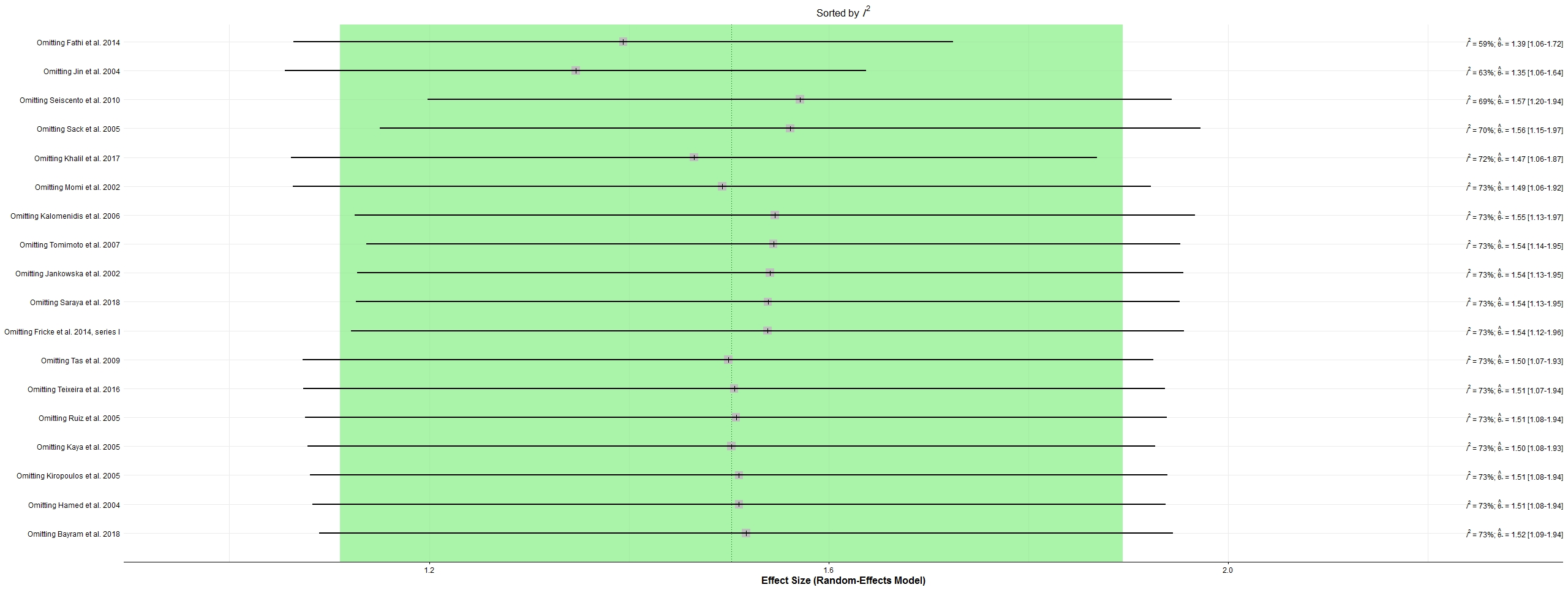

Supplement: S12 Fig — (TIF) [file pone.0268543.s013.tif]

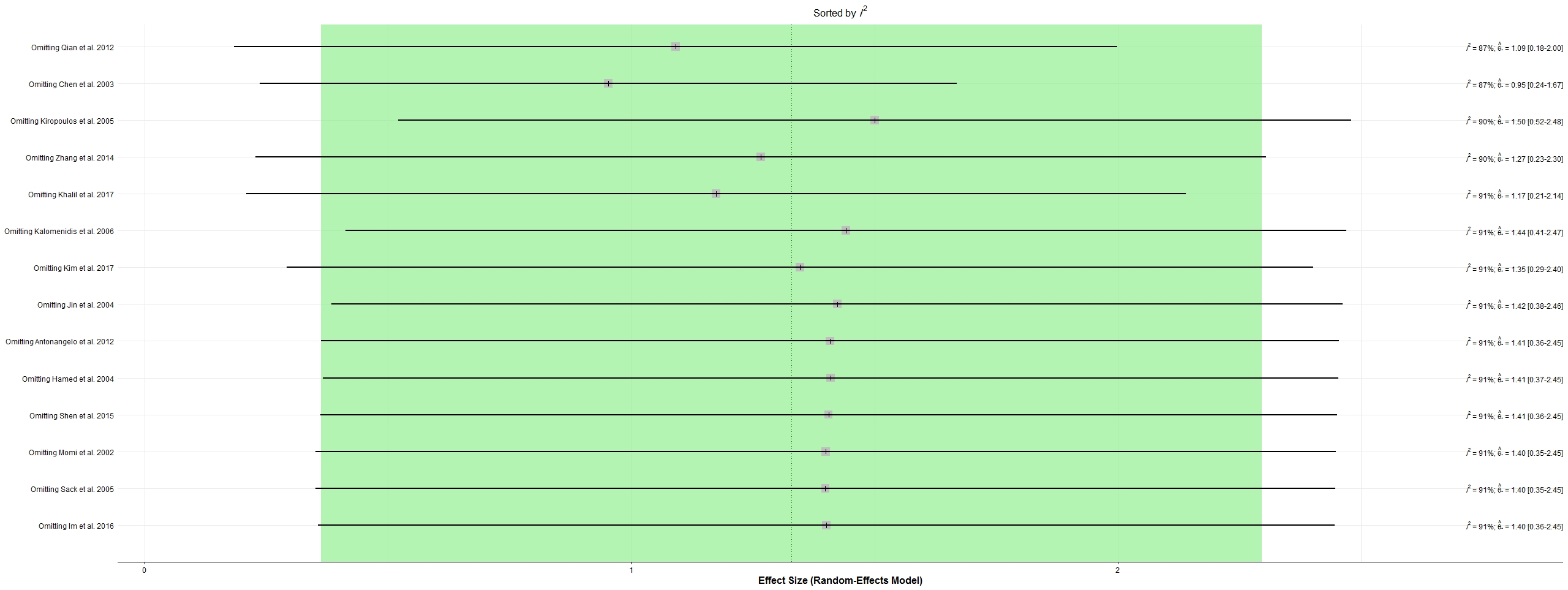

Supplement: S13 Fig — (TIF) [file pone.0268543.s014.tif]

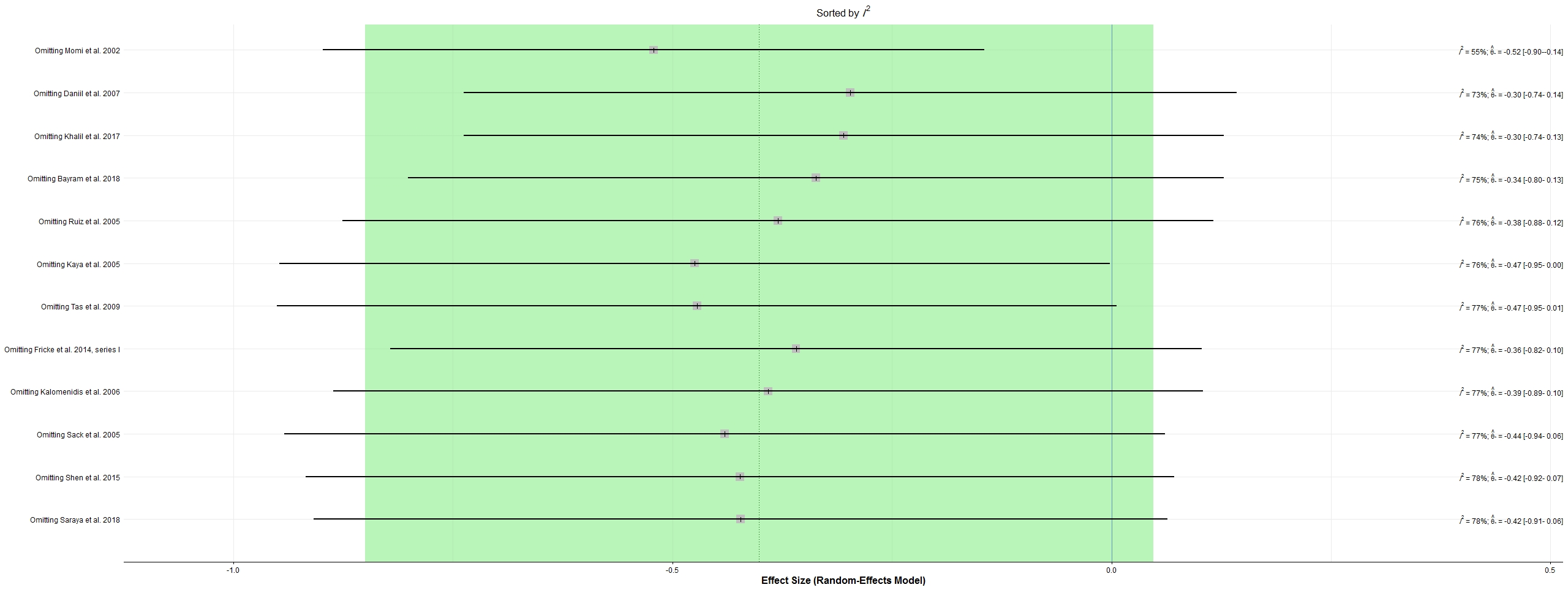

Supplement: S14 Fig — (TIF) [file pone.0268543.s015.tif]

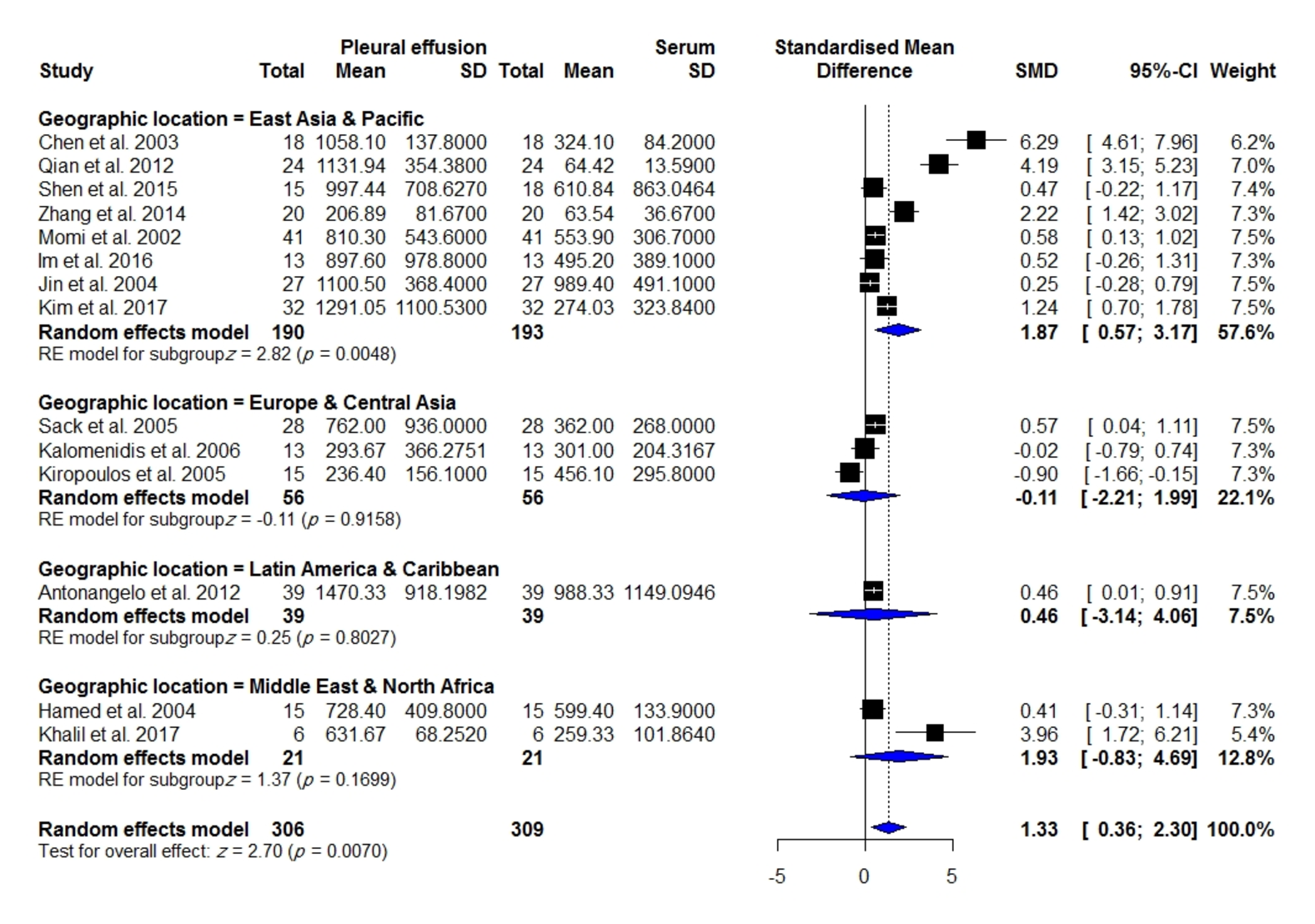

Supplement: S15 Fig — (TIF) [file pone.0268543.s016.tif]

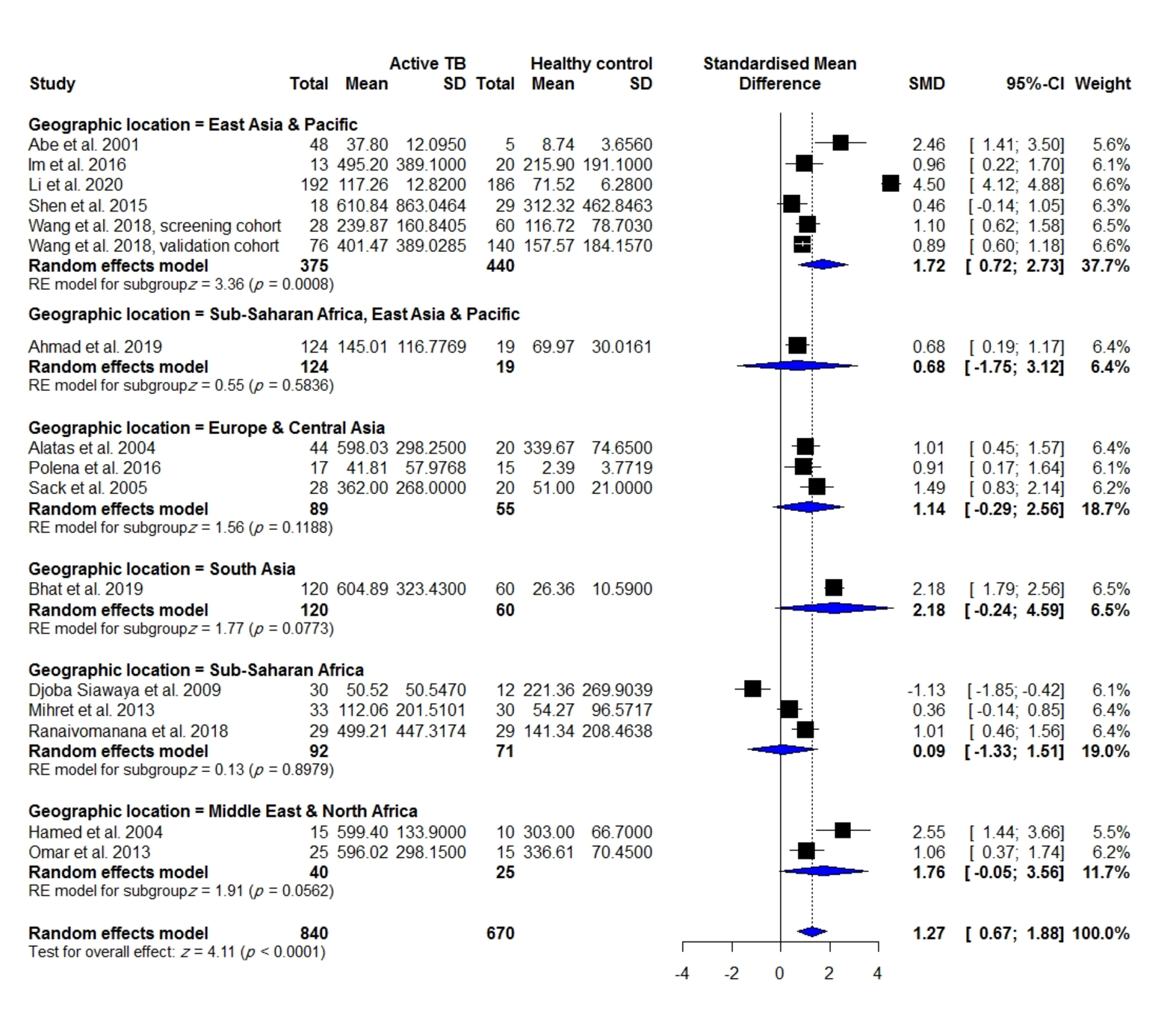

Supplement: S16 Fig — (TIF) [file pone.0268543.s017.tif]

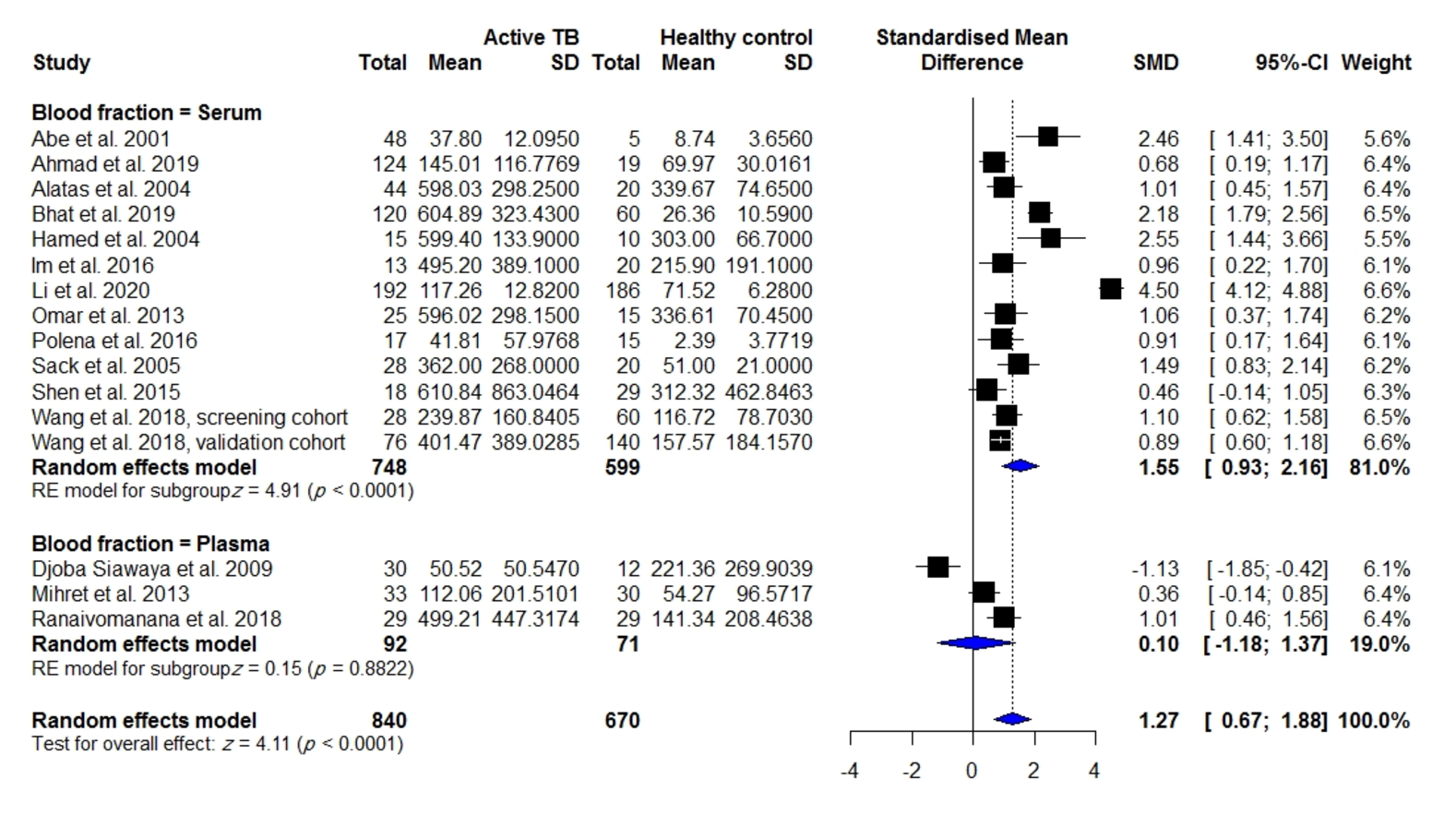

Supplement: S17 Fig — (TIF) [file pone.0268543.s018.tif]

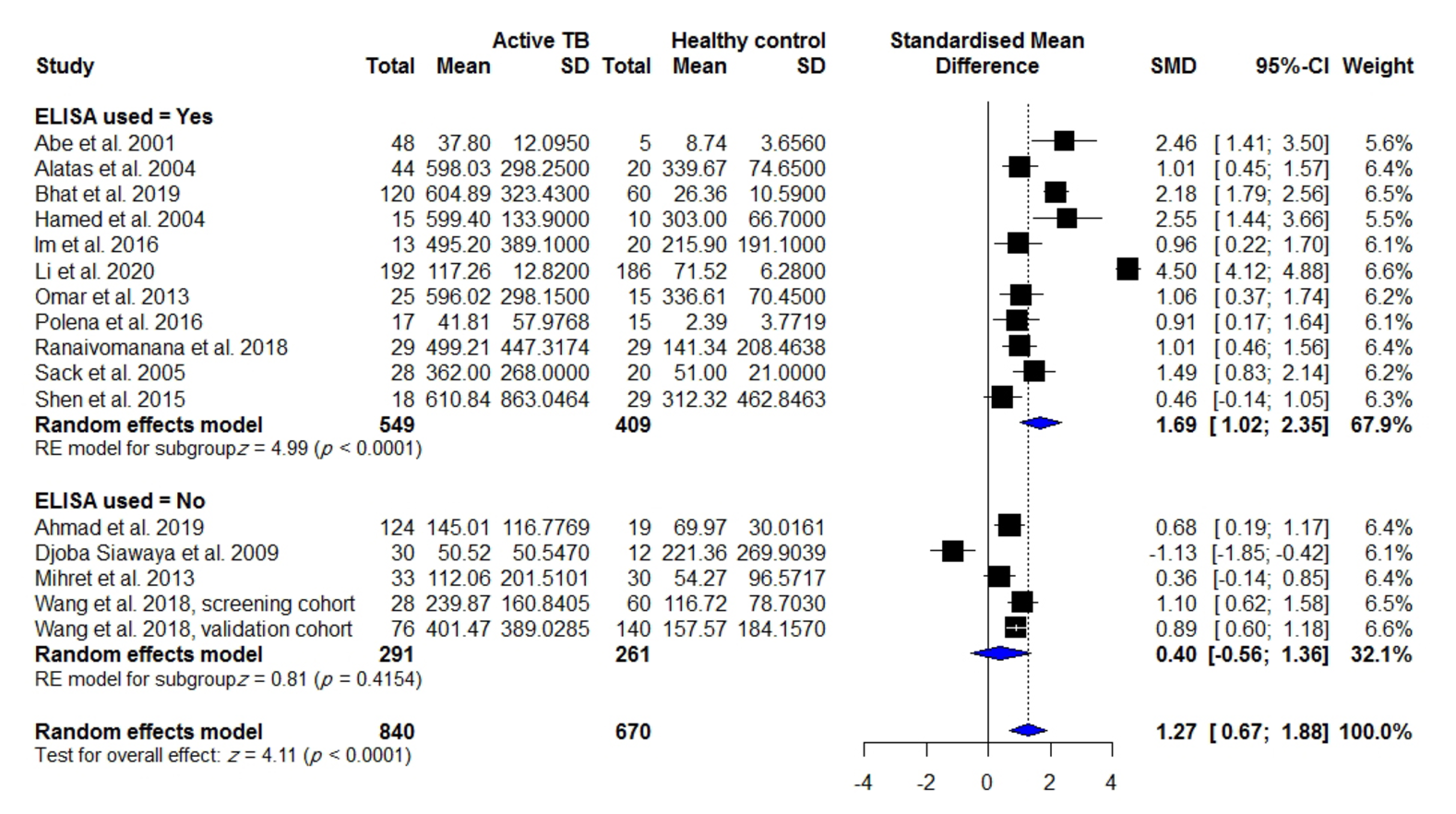

Supplement: S18 Fig — (TIF) [file pone.0268543.s019.tif]

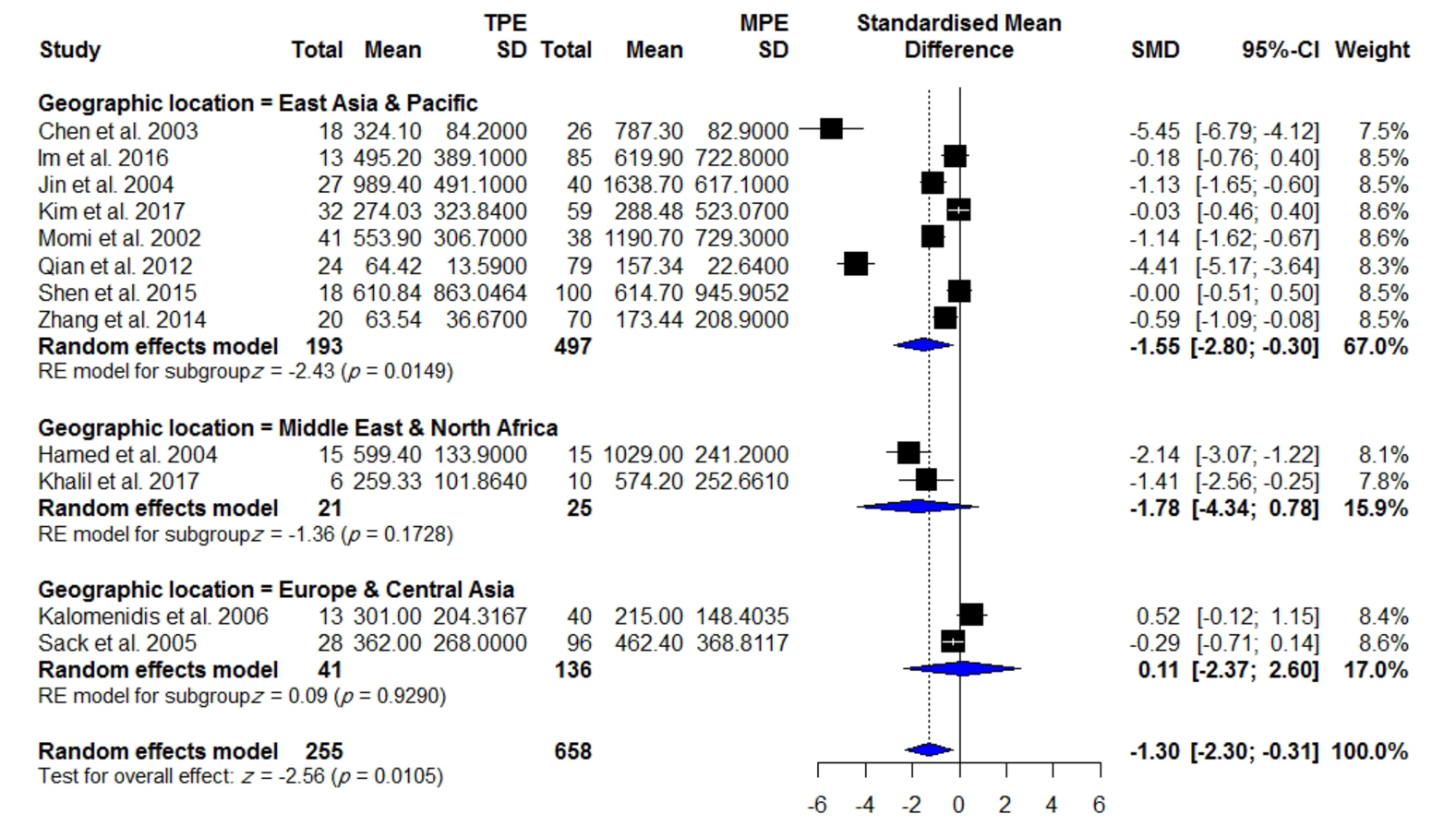

Supplement: S19 Fig — (TIF) [file pone.0268543.s020.tif]

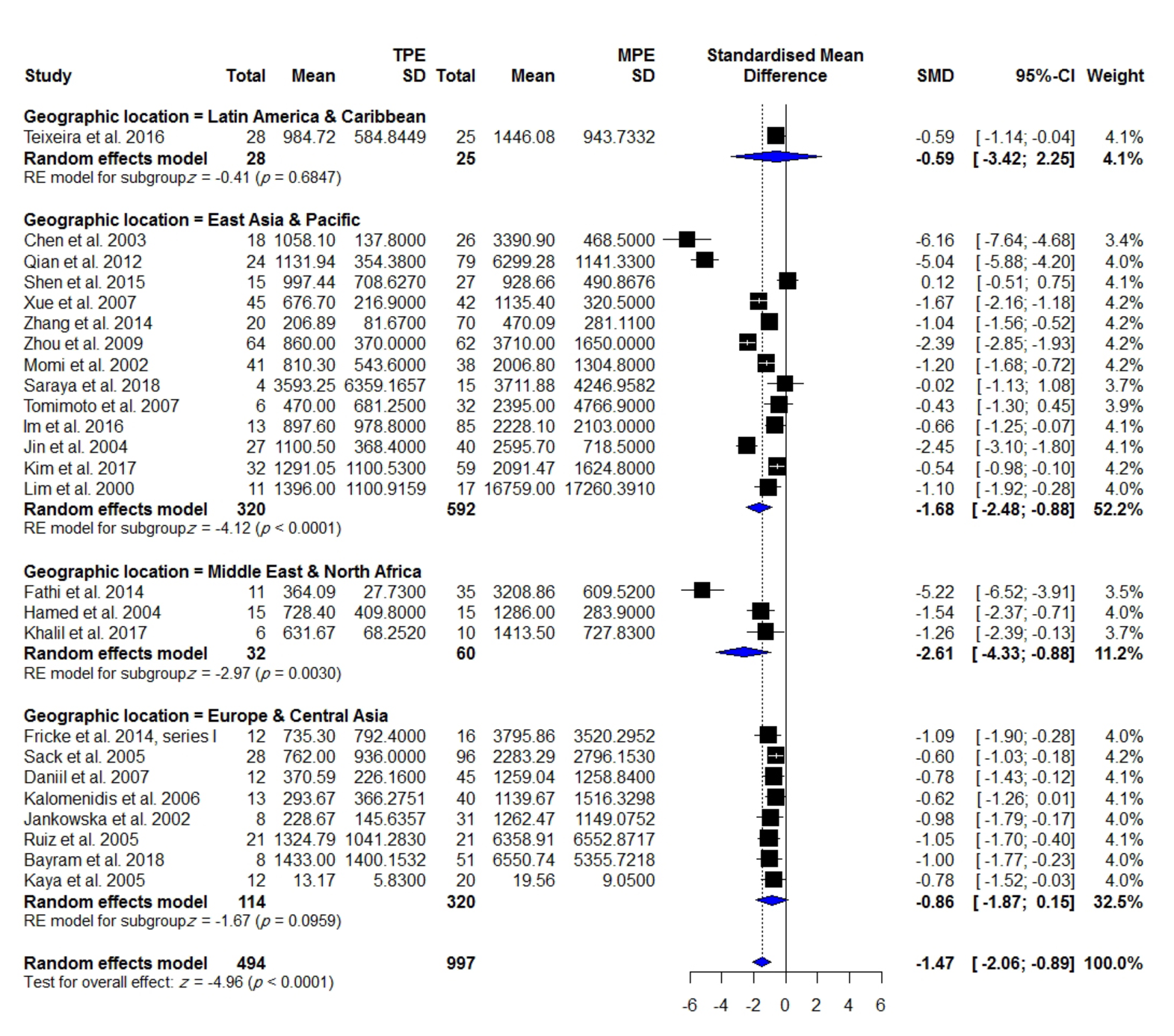

Supplement: S20 Fig — (TIF) [file pone.0268543.s021.tif]

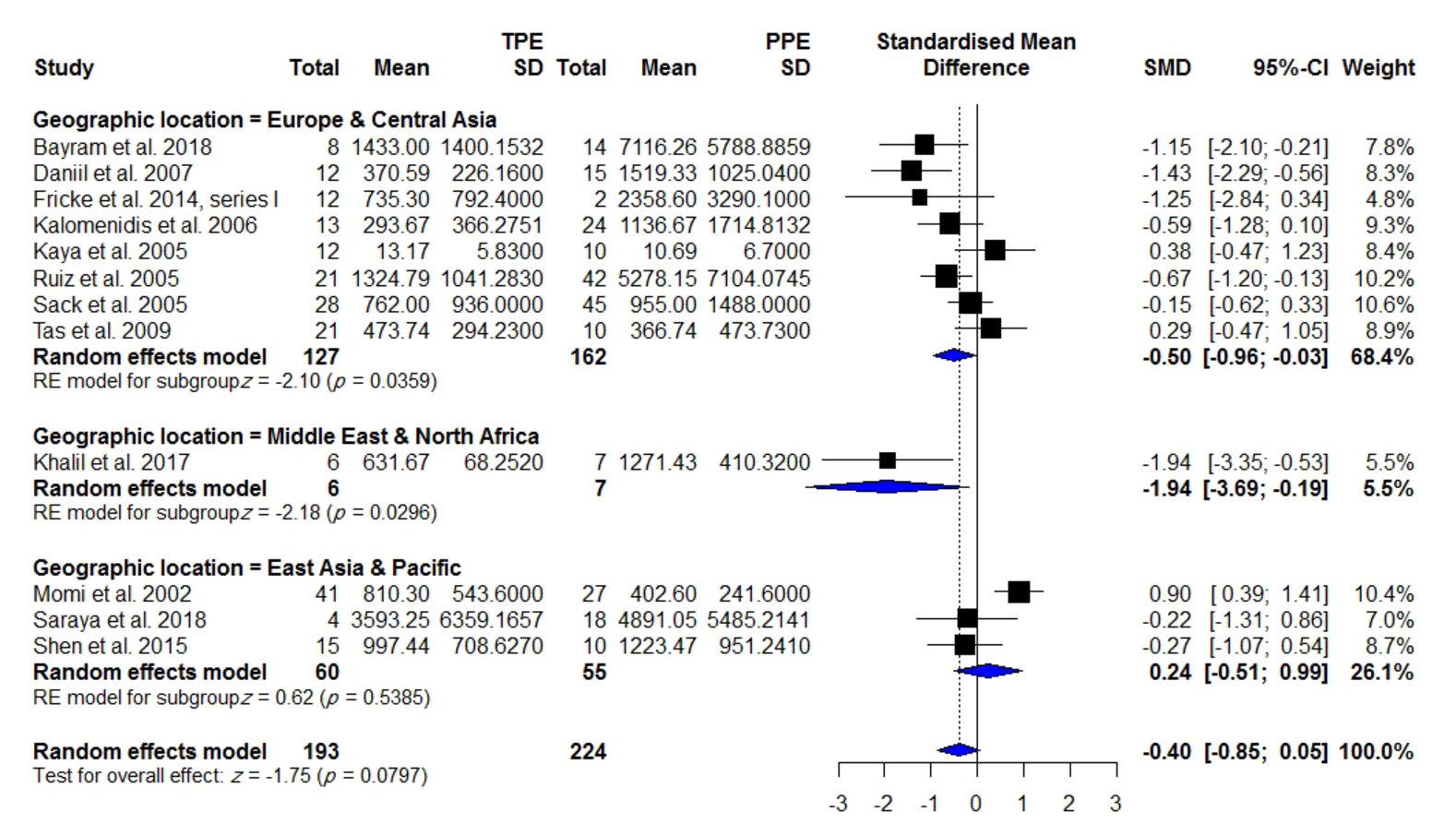

Supplement: S21 Fig — (TIF) [file pone.0268543.s022.tif]

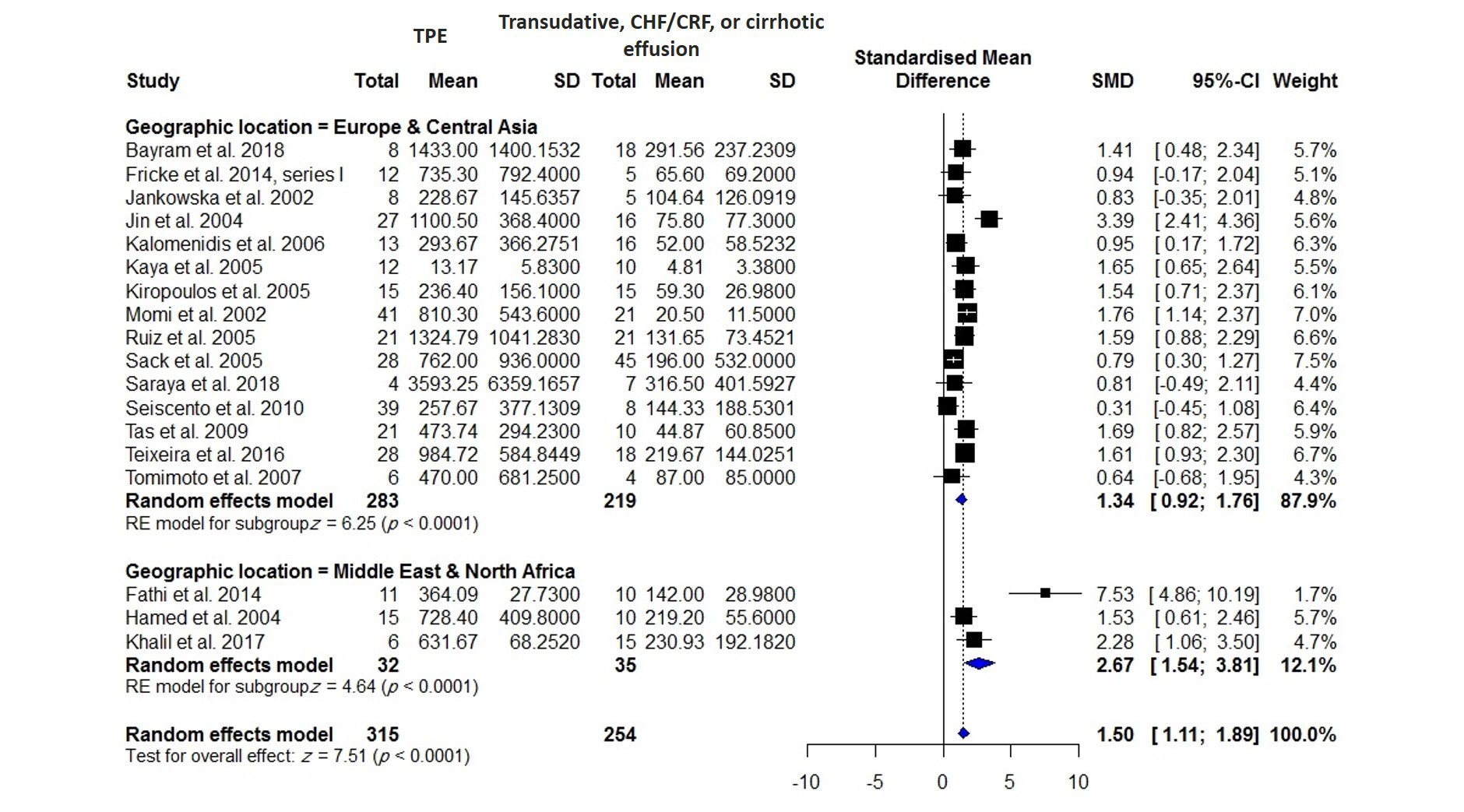

Supplement: S22 Fig — (TIF) [file pone.0268543.s023.tif]
