# Supplementary material for: Vascular endothelial growth factor levels in tuberculosis: A systematic review and meta-analysis
Source: PLoS One. 2022 May 25;17(5):e0268543. doi: 10.1371/journal.pone.0268543 (PMC9132289; doi:10.1371/journal.pone.0268543)
Supplement: S1 Table — (DOCX) [file pone.0268543.s024.docx]

| **Study** | **Location(s)** | **Case** | | | | **Control** | | | | **Sample** | **Assay** |
| --- | --- | --- | --- | --- | --- | --- | --- | --- | --- | --- | --- |
|  |  | **Condition** | **N** | **Mean age** | **Male%** | **Condition** | **N** | **Mean age** | **Male%** |  |  |
| Abe et al. 2001 [1] | Japan | Active TB | 48 | 57.7 | 72.9 | Healthy control | 5 |  |  | Serum | ELISA |
| Ahmad et al. 2019 [2] | SA and Vietnam | Active TB | 124 |  | 71 | Healthy control with latent TB | 19 |  | 52.6 | Serum | Simoa |
| Alatas et al. 2004 [3] | Turkey | Active TB | 44 | 43.2 | 70.5 | Healthy control | 20 | 36.6 |  | Serum | ELISA |
| Antonangelo et al. 2012 [4] | Brazil | TPE | 39 | 35 |  |  |  |  |  | PF, serum | ELISA |
| Bayram et al. 2018 [5] | Turkey | TPE | 8 | 30.37 |  | Primary lung cancer | 17 |  |  | PF | ELISA |
|  |  |  |  |  |  | Other malignancy | 25 |  |  |  |  |
|  |  |  |  |  |  | PPE | 14 |  |  |  |  |
|  |  |  |  |  |  | Transudative effusion | 18 |  |  |  |  |
| Bhat et al. 2019 [6] | India | Active PTB | 120 | 42.5 | 62.5 | Healthy control | 60 | 44.8 | 53.3 | Serum | ELISA |
| Chen et al. 2003 [7] | China | TPE | 18 |  |  | MPE | 26 |  |  | PF, serum | ELISA |
| Daniil et al. 2007 [8] | Greece | TPE | 12 |  |  | MPE | 45 |  |  | PF | ELISA |
|  |  |  |  |  |  | PPE | 15 |  |  | PF | ELISA |
| Djoba Siawaya et al. 2009 [9] | South Africa | Active PTB | 19 | 32 |  | Healthy control with latent TB | 12 | 35 |  | Plasma | Bio-Plex platform |
|  |  | TPE | 11 |  |  |  |  |  |  |  |  |
| Dong et al. 2003 [10] | China | TBA | 8 | 28 | 50 | Cirrhotic ascites | 36 | 48 | 72.22 | AF | ELISA |
|  |  |  |  |  |  | Malignant ascites | 23 | 66 | 47.83 |  |  |
| Fathi et al. 2014 [11] | Egypt | TPE | 11 |  |  | MPE | 35 |  |  | PF | ELISA |
|  |  |  |  |  |  | Transudative effusion | 10 | 60 | 60 |  |  |
| Fricke et al. 2014 [12] | Germany | TPE | 12 | 52.1 | 75 | Lung cancer | 10 | 78.1 | 50 | PF | ELISA |
|  |  |  |  |  |  | CHF | 5 | 78.6 | 40 |  |  |
|  |  |  |  |  |  | PPE | 2 | 80.5 | 100 |  |  |
|  |  |  |  |  |  | Other malignancy | 6 | 66 | 16.7 |  |  |
| Hamed et al. 2004 [13] | Egypt | TPE | 15 | 29.5 |  | Healthy control | 10 | 30 |  | PF, serum | ELISA |
|  |  |  |  |  |  | Transudative effusion | 10 | 33.4 |  |  |  |
|  |  |  |  |  |  | MPE | 15 | 34.9 |  |  |  |
| Husain et al. 2008 [14] | India | Active TBM | 20 | 20.7 | 75 | Inactive TBM | 20 | 22.7 | 70 | CSF, serum | ELISA |
| Im et al. 2016 [15] | Korea | TPE | 13 | 44.6 | 69.2 | Lung cancer | 85 | 64.5 | 75.3 | PF, serum | ELISA |
|  |  |  |  |  |  | Healthy control | 20 | 54.6 | 55 |  |  |
| Jankowska et al. 2002 [16] | Poland | TPE | 8 |  |  | MPE | 31 |  |  | PF | ELISA |
|  |  |  |  |  |  | Transudative effusion | 5 |  |  |  |  |
| Jin et al. 2004 [17] | Korea | TPE | 27 | 49.1 | 51.85 | Lung cancer | 40 | 58.3 | 60 | PF, serum | ELISA |
|  |  |  |  |  |  | Cirrhosis | 16 | 54.8 | 62.5 |  |  |
| Kalomenidis et al. 2006 [18] | Greece | TPE | 13 |  |  | MPE | 40 |  |  | PF, serum | ELISA |
|  |  |  |  |  |  | PPE | 24 |  |  |  |  |
|  |  |  |  |  |  | HF | 16 |  |  |  |  |
| Kaya et al. 2005 [19] | Turkey | TPE | 12 | 41.2 |  | MPE | 20 | 63.86 |  | PF | ELISA |
|  |  |  |  |  |  | PPE | 10 | 59.1 |  |  |  |
|  |  |  |  |  |  | Transudative effusion | 10 | 67.54 |  |  |  |
| Khalil et al. 2017 [20] | Egypt | TPE | 6 |  |  | Transudative effusion | 15 | 59 | 33.33 | PF, serum |  |
|  |  |  |  |  |  | MPE | 10 |  |  |  |  |
|  |  |  |  |  |  | PPE | 7 |  |  |  |  |
| Kim et al. 2017 [21] | Korea | TPE | 32 | 55.06 | 68.75 | MPE | 59 | 68.73 | 66.1 | PF, serum | ELISA |
| Kiropoulos et al. 2005 [22] | Greece | TPE | 15 | 61.26 | 60 | CHF | 15 | 69.23 | 80 | PF, serum | ELISA |
| Li et al. 2020 [23] | China | Active TB | 192 | 47.24 | 61.98 | Healthy control | 186 | 45.63 | 54.3 | Serum | ELISA |
| Lim et al. 2000 [24] | Korea | TPE | 11 | 52.2 | 81.82 | MPE | 17 | 59.5 | 47.06 | PF | ELISA |
| Liu et al. 2010 [25] | Germany | TB pericardial effusion | 33 | 38.75 | 54.55 | Malignant pericardial effusion | 47 | 47.85 | 53.19 | Pericardial fluid | ELISA |
| Matsuyama et al. 2001 [26] | Japan | TBM | 28 | 50.7 | 64.29 | BM | 13 | 52.1 | 61.54 | CSF, serum | ELISA |
|  |  |  |  |  |  | FM | 8 | 53.9 | 75 |  |  |
|  |  |  |  |  |  | VM | 10 | 48.9 | 80 |  |  |
|  |  |  |  |  |  | Non-infectious disease | 8 | 53.3 |  |  |  |
| Mihret et al. 2013 [27] | Ethiopia | Active TB | 33 | 32.4 | 51.5 | Household contacts | 30 | 29.7 | 52.6 | Plasma | Luminex platform |
| Misra et al. 2013 [28] | India | TBM | 40 | 32.75 | 55 | Healthy control | 14 | Rang: 23-31 ys | 71.43 | Serum | ELISA |
| Momi et al. 2002 [29] | Japan | TPE | 41 |  |  | CHF | 21 |  |  | PF, serum | ELISA |
|  |  |  |  |  |  | MPE | 38 |  |  |  |  |
|  |  |  |  |  |  | PPE | 27 |  |  |  |  |
| Omar et al. 2013 [30] | Egypt | Active PTB | 25 | 36.75 | 80 | Healthy control | 15 | 26.2 | 66.67 | Serum | ELISA |
| Polena et al. 2016 [31] | France | Active PTB | 17 |  |  | Contacts | 7 |  |  | Serum | ELISA |
|  |  |  |  |  |  | Healthy control | 8 |  |  |  |  |
| Qama et al. 2012 [32] | Korea | TPE | 10 | 48 | 40 | Healthy control | 10 | 45 | 90 | BALF | Bio-Plex platform |
| Qian et al. 2012 [33] | China | TPE | 24 |  | 79 | Lung cancer | 79 |  | 61 | PF, serum | ELISA |
| Qiu et al. 2009 [34] | China | TBM | 34 |  |  | VM | 29 |  |  | CSF, serum | ELISA |
| Ranaivomanana et al. 2018 [35] | Madagascar | EPTB | 16 | 32 | 64.3 | Healthy control | 29 | 32 | 48.3 | Plasma | ELISA |
|  |  | PTB | 13 | 30 | 69.2 |  |  |  |  |  |  |
| Ruiz et al. 2005 [36] | Spain | TPE | 21 |  |  | Empyema and  PPE | 42 |  |  | PF | ELISA |
|  |  |  |  |  |  | MPE | 21 |  |  |  |  |
|  |  |  |  |  |  | Transudative effusion | 21 |  |  |  |  |
| Sack et al. 2005 [37] | Germany | TPE | 28 | 50.9 | 82.14286 | Lung cancer | 58 | 68.9 | 65.51724 | PF, serum | ELISA |
|  |  |  |  |  |  | Other malignancy | 38 | 67.6 | 40.74074 |  |  |
|  |  |  |  |  |  | PPE | 45 | 57.7 | 82.22222 |  |  |
|  |  |  |  |  |  | CHF | 45 | 70.7 | 73.07692 |  |  |
|  |  |  |  |  |  | Healthy control | 20 | 35.1 | 60 |  |  |
| Safe et al. 2021 [38] | Brazil | TB | 21 |  |  |  |  |  |  | Plasma | Luminex platform |
| Saraya et al. 2018 [39] | Japan | TPE | 4 | 62.5 | 75 | CHF/CRF | 7 | 85 | 57.1 | PF |  |
|  |  |  |  |  |  | MPE | 15 | 72 | 46.7 |  |  |
|  |  |  |  |  |  | Empyema and PPE | 18 | 70.5 | 72.2 |  |  |
| Seiscento et al. 2010 [40] | Brazil | TPE | 39 | 35.8 | 79.49 | CHF | 8 | 64.5 | 100 | PF | ELISA |
| Shen et al. 2015 [41] | China | TPE | 18 | 51.33 | 83.33 | PPE | 47 | 59.3 | 55.32 | PF, serum | ELISA |
|  |  |  |  |  |  | Lung cancer | 100 | 69.79 | 69 |  |  |
|  |  |  |  |  |  | Healthy control | 29 | 36.62 | 62.07 |  |  |
| Tai et al. 2017 [42] | Malaysia | TBM | 15 | 36 | 40 |  |  |  |  | CSF, serum | ELISA |
| Tas et al. 2009 [43] | Turkey | TPE | 21 | 25.42 | 95.24 | PPE | 10 | 32.5 | 80 | PF | ELISA |
|  |  |  |  |  |  | CHF | 10 | 67.9 | 90 |  |  |
| Teixeira et al. 2016 [44] | Brazil | TPE | 41 | 41.8 | 68.3 | MPE | 39 | 57 | 56.4 | PF | ELISA |
|  |  |  |  |  |  | Transudative effusion | 34 | 62.5 | 64.7 |  |  |
| Tomimoto et al. 2007 [45] | Japan | TPE | 6 |  |  | Lung cancer | 22 |  |  | PF | ELISA |
|  |  |  |  |  |  | Other malignancy | 10 |  |  |  |  |
|  |  |  |  |  |  | CHF | 4 |  |  |  |  |
| van der Flier et al. 2004 [46] | Netherlands | TBM | 26 | 3.1 | 46.2 | Fever without meningitis | 20 | 5.2 | 80 | Plasma | ELISA |
| Visser et al. 2015 [47] | South Africa | TBM | 56 | 4.09 | 53.6 | VM | 25 |  |  | CSF | Luminex platform |
|  |  |  |  |  |  | BM | 10 |  |  |  |  |
| Wang et al. 2018, validation cohort [48] | China | Active TB | 76 | 45 | 55.3 | Healthy control with latent TB | 69 | 42.33333 | 46.4 | Serum | Bio-Plex platform |
|  |  |  |  |  |  | Healthy uninfected control | 71 | 42.66667 | 49.3 |  |  |
| Wang et al. 2018, screening cohort [48] | China | Active TB | 28 | 42.33333 | 57.1 | Healthy control with latent TB | 34 | 40 | 44.1 |  |  |
|  |  |  |  |  |  | Healthy uninfected control | 26 | 39.33333 | 46.2 |  |  |
| Xue et al. 2007 [49] | China | TPE | 45 | 48.5 | 55.56 | MPE | 42 | 51.4 | 52.38 | PF | ELISA |
| Zhan et al. 2016 [50] | China | TBA | 167 | 40.89 | 68.26 | Malignant ascites | 462 | 52.17 | 45.24 | AF | ELISA |
|  |  |  |  |  |  | Cirrhotic ascites | 189 | 48.38 | 73.02 |  |  |
|  |  |  |  |  |  | CHF | 97 | 61.9 | 72.17 |  |  |
| Zhang et al. 2014 [51] | China | TPE | 20 |  | 75 | Lung cancer | 70 |  | 65.7 | PF, serum | ELISA |
| Zhou et al. 2009 [52] | China | TPE | 64 | 42.36 | 57.81 | Lung cancer | 62 | 51.27 | 62.9 | PF | ELISA |

1. Abe Y, Nakamura M, Oshika Y, Hatanaka H, Tokunaga T, Ohkubo Y, Hashizume T, Suzuki K, Fujino T (2001) Serum levels of vascular endothelial growth factor and cavity formation in active pulmonary tuberculosis. Respiration; international review of thoracic diseases 68 (5):496-500. doi:10.1159/000050557

2. Ahmad R, Xie L, Pyle M, Suarez MF, Broger T, Steinberg D, Ame SM, Lucero MG, Szucs MJ, MacMullan M, Berven FS, Dutta A, Sanvictores DM, Tallo VL, Bencher R, Eisinger DP, Dhingra U, Deb S, Ali SM, Mehta S, Fawzi WW, Riley ID, Sazawal S, Premji Z, Black R, Murray CJL, Rodriguez B, Carr SA, Walt DR, Gillette MA (2019) A rapid triage test for active pulmonary tuberculosis in adult patients with persistent cough. Science translational medicine 11 (515). doi:10.1126/scitranslmed.aaw8287

3. Alatas F, Alatas O, Metintas M, Ozarslan A, Erginel S, Yildirim H (2004) Vascular endothelial growth factor levels in active pulmonary tuberculosis. Chest 125 (6):2156-2159. doi:10.1378/chest.125.6.2156

4. Antonangelo L, Vargas FS, Puka J, Seiscento M, Acencio MM, Teixeira LR, Terra RM, Sales RK (2012) Pleural tuberculosis: is radiological evidence of pulmonary-associated disease related to the exacerbation of the inflammatory response? Clinics (Sao Paulo, Brazil) 67 (11):1259-1263. doi:10.6061/clinics/2012(11)06

5. Bayram N, Karakan Y, Uyar M, Ozyurt B, Filiz A (2018) Vascular endothelial growth factor in pleural effusions and correlation with radiologic and biochemical parameters. Nigerian journal of clinical practice 21 (1):59-62. doi:10.4103/njcp.njcp_370_16

6. Bhat H, Ambekar JG, Harwalkar AK, Dongre N, Das KK (2019) Serum VEGF and TNF-α correlate bacterial burden in pulmonary tuberculosis. Indian Journal of Public Health Research and Development 10 (1):189-194. doi:10.5958/0976-5506.2019.00039.1

7. Chen Y-f, Tang Y-x, Jiang S-f (2003) The Significance of Detecting Vascular Endothelial Growth Factor in Differentiating Tuberculosis Pleural Effusion from Malignant Pleural Effusions. The Practical Journal of Cancer:03

8. Daniil ZD, Zintzaras E, Kiropoulos T, Papaioannou AI, Koutsokera A, Kastanis A, Gourgoulianis KI (2007) Discrimination of exudative pleural effusions based on multiple biological parameters. The European respiratory journal 30 (5):957-964. doi:10.1183/09031936.00126306

9. Djoba Siawaya JF, Chegou NN, van den Heuvel MM, Diacon AH, Beyers N, van Helden P, Walzl G (2009) Differential cytokine/chemokines and KL-6 profiles in patients with different forms of tuberculosis. Cytokine 47 (2):132-136. doi:10.1016/j.cyto.2009.05.016

10. Dong WG, Sun XM, Yu BP, Luo HS, Yu JP (2003) Role of VEGF and CD44v6 in differentiating benign from malignant ascites. World journal of gastroenterology 9 (11):2596-2600. doi:10.3748/wjg.v9.i11.2596

11. Fathy M, Al Ansary M, Zakaria M, Abdel-Hafiz H, Said M (2014) Role of vascular endothelial growth factor (VEGF) in diagnosis of pleural effusion of different origins. Egyptian Journal of Chest Diseases and Tuberculosis 63 (3):611-615

12. Fricke S, Hoheisel G, Gessner C, Bauer K, Seyfarth HJ, Hammerschmidt S, Kahlenberg F, Chan K, Boldt A, Wirtz H, Keller T, Sack U (2014) Mediators in pleural effusions of different origin: a two-step diagnostic study. Laboratoriumsmedizin-Journal of Laboratory Medicine 38 (3):121-127. doi:10.1515/labmed-2014-0006

13. Hamed EA, El-Noweihi AM, Mohamed AZ, Mahmoud A (2004) Vasoactive mediators (VEGF and TNF-alpha) in patients with malignant and tuberculous pleural effusions. Respirology (Carlton, Vic) 9 (1):81-86. doi:10.1111/j.1440-1843.2003.00529.x

14. Husain N, Awasthi S, Haris M, Gupta RK, Husain M (2008) Vascular endothelial growth factor as a marker of disease activity in neurotuberculosis. The Journal of infection 56 (2):114-119. doi:10.1016/j.jinf.2007.11.004

15. Im BK, Oh YJ, Sheen SS, Lee KS, Park KJ, Hwang SC, Lee YH, Choi JH, Lim HY (2016) Clinical Significance of Vascular Endothelial Growth Factor in Patients with Lung Cancer and Tuberculous Pleurisy. Tuberculosis and Respiratory Diseases 50 (2):171-181

16. Jankowska R, Porebska I, Dyła T (2002) [Evaluation of vascular endothelial growth factor (VEGF) in neoplastic and tuberculosis effusions--preliminary results]. Pneumonologia i alergologia polska 70 (5-6):258-264

17. Jin HY, Lee KS, Jin SM, Lee YC (2004) Vascular endothelial growth factor correlates with matrix metalloproteinase-9 in the pleural effusion. Respiratory medicine 98 (2):115-122. doi:10.1016/j.rmed.2003.09.002

18. Kalomenidis I, Kollintza A, Sigala I, Papapetropoulos A, Papiris S, Light RW, Roussos C (2006) Angiopoietin-2 levels are elevated in exudatative pleural effusions. Chest 129 (5):1259-1266. doi:10.1378/chest.129.5.1259

19. Kaya A, Poyraz B, Celik G, Ciledag A, Gulbay BE, Savas H, Savas I (2005) [Vascular endothelial growth factor in benign and malignant pleural effusions]. Archivos de bronconeumologia 41 (7):376-379. doi:10.1016/s1579-2129(06)60244-9

20. Khalil NH, Abdelaal DE (2017) Vascular endothelial growth factor in diagnosis of pleural effusion. Egyptian Journal of Chest Diseases and Tuberculosis 66 (1):115-119. doi:10.1016/j.ejcdt.2016.12.006

21. Kim HR, Kim BR, Park RK, Yoon KH, Jeong ET, Hwang KE (2017) Diagnostic Significance of Measuring Vascular Endothelial Growth Factor for the Differentiation between Malignant and Tuberculous Pleural Effusion. The Tohoku journal of experimental medicine 242 (2):137-142. doi:10.1620/tjem.242.137

22. Kiropoulos TS, Kostikas K, Gourgoulianis KI, Alatas F, Alatas O, Metintas M, Ozarslan A, Erginel S, Yildirim H (2005) Vascular endothelial growth factor levels in pleural fluid and serum of patients with tuberculous pleural effusions [6] (multiple letters). Chest 128 (1):468-469. doi:10.1378/chest.128.1.468

23. Li ZY, Li CL, Bao RR, Liu ZD (2020) Expressions of miR-29a, TNF-A and Vascular Endothelial Growth Factor in Peripheral Blood of Pulmonary Tuberculosis Patients and Their Clinical Significance. Iranian journal of public health 49 (9):1683-1691

24. Lim SC, Jung SI, Kim YC, Park KO (2000) Vascular endothelial growth factor in malignant and tuberculous pleural effusions. Journal of Korean medical science 15 (3):279-283. doi:10.3346/jkms.2000.15.3.279

25. Liu J, Zeng Y, Ma W, Chen S, Zheng Y, Ye S, Lan L, Weig HJ, Liu Q (2010) Preliminary investigation of the clinical value of vascular endothelial growth factor and hypoxia-inducible factor-1alpha in pericardial fluid in diagnosing malignant and tuberculous pericardial effusion. Cardiology 116 (1):37-41. doi:10.1159/000313465

26. Matsuyama W, Hashiguchi T, Umehara F, Matsuura E, Kawabata M, Arimura K, Maruyama I, Osame M (2001) Expression of vascular endothelial growth factor in tuberculous meningitis. Journal of the neurological sciences 186 (1-2):75-79. doi:10.1016/s0022-510x(01)00515-9

27. Mihret A, Bekele Y, Bobosha K, Kidd M, Aseffa A, Howe R, Walzl G (2013) Plasma cytokines and chemokines differentiate between active disease and non-active tuberculosis infection. The Journal of infection 66 (4):357-365. doi:10.1016/j.jinf.2012.11.005

28. Misra UK, Kalita J, Singh AP, Prasad S (2013) Vascular endothelial growth factor in tuberculous meningitis. The International journal of neuroscience 123 (2):128-132. doi:10.3109/00207454.2012.743127

29. Momi H, Matsuyama W, Inoue K, Kawabata M, Arimura K, Fukunaga H, Osame M (2002) Vascular endothelial growth factor and proinflammatory cytokines in pleural effusions. Respiratory medicine 96 (10):817-822. doi:10.1053/rmed.2002.1364

30. Omar M, ElAdl T, Abdullah S, Hamza H, ElAdl T, Neamatallah M (2013) Clinical Implications for Vascular Endothelial Growth Factor Levels among Egyptians with Pulmonary Tuberculosis. Life Science Journal-Acta Zhengzhou University Overseas Edition 10 (1):2978-2983

31. Polena H, Boudou F, Tilleul S, Dubois-Colas N, Lecointe C, Rakotosamimanana N, Pelizzola M, Andriamandimby SF, Raharimanga V, Charles P, Herrmann JL, Ricciardi-Castagnoli P, Rasolofo V, Gicquel B, Tailleux L (2016) Mycobacterium tuberculosis exploits the formation of new blood vessels for its dissemination. Scientific reports 6:33162. doi:10.1038/srep33162

32. Qama D, Choi WI, Kwon KY (2012) Immune responses in the lungs of patients with tuberculous pleural effusion without pulmonary tuberculosis. BMC immunology 13:45. doi:10.1186/1471-2172-13-45

33. Qian Q, Zhan P, Sun WK, Zhang Y, Song Y, Yu LK (2012) Vascular endothelial growth factor and soluble intercellular adhesion molecule-1 in lung adenocarcinoma with malignant pleural effusion: correlations with patient survival and pleural effusion control. Neoplasma 59 (4):433-439. doi:10.4149/neo_2012_056

34. Qiu B, Li G, Fan X, Xing X (2009) Diagnostic value of vascular endothelial growth factor and adenosine deaminase detection in differentiating tuberculous meningitis from viral meningitis. Medical Journal of Wuhan University 30 (6):806-809

35. Ranaivomanana P, Raberahona M, Rabarioelina S, Borella Y, Machado A, Randria MJD, Rakotoarivelo RA, Rasolofo V, Rakotosamimanana N (2018) Cytokine Biomarkers Associated with Human Extra-Pulmonary Tuberculosis Clinical Strains and Symptoms. Frontiers in microbiology 9:275. doi:10.3389/fmicb.2018.00275

36. Ruiz E, Alemán C, Alegre J, Monasterio J, Segura RM, Armadans L, Vázquez A, Soriano T, Fernández de Sevilla T (2005) Angiogenic factors and angiogenesis inhibitors in exudative pleural effusions. Lung 183 (3):185-195. doi:10.1007/s00408-004-2533-0

37. Sack U, Hoffmann M, Zhao XJ, Chan KS, Hui DS, Gosse H, Engelmann L, Schauer J, Emmrich F, Hoheisel G (2005) Vascular endothelial growth factor in pleural effusions of different origin. The European respiratory journal 25 (4):600-604. doi:10.1183/09031936.05.00037004

38. Safe IP, Amaral EP, Araújo-Pereira M, Lacerda MVG, Printes VS, Souza AB, Beraldi-Magalhães F, Monteiro WM, Sampaio VS, Barreto-Duarte B, Andrade AMS, Spener-Gomes R, Costa AG, Cordeiro-Santos M, Andrade BB (2021) Adjunct N-Acetylcysteine Treatment in Hospitalized Patients With HIV-Associated Tuberculosis Dampens the Oxidative Stress in Peripheral Blood: Results From the RIPENACTB Study Trial. Frontiers in immunology 11. doi:10.3389/fimmu.2020.602589

39. Saraya T, Ohkuma K, Watanabe T, Mikura S, Kobayashi F, Aso J, Nunokawa H, Honda K, Ogawa Y, Tamura M, Sada M, Oda M, Inoue M, Yokoyama T, Kurai D, Ishii H, Kimura H, Takizawa H (2018) Diagnostic Value of Vascular Endothelial Growth Factor, Transforming Growth Factor-beta, Interleukin-8, and the Ratio of Lactate Dehydrogenase to Adenosine Deaminase in Pleural Effusion. Lung 196 (2):249-254. doi:10.1007/s00408-018-0090-1

40. Seiscento M, Vargas FS, Acencio MM, Teixeira LR, Capelozzi VL, Sales RK, Antonangelo L (2010) Pleural fluid cytokines correlate with tissue inflammatory expression in tuberculosis. The international journal of tuberculosis and lung disease : the official journal of the International Union against Tuberculosis and Lung Disease 14 (9):1153-1158

41. Shen H, Feng GZ, Cui J, Du Q, Qin Y, Cai JK, Shen L, Zhu YN (2015) Clinical implications of serum hypoxia inducible factor-1 alpha and vascular endothelial growth factor in lung cancer. Tumori 101 (4):404-411. doi:10.5301/tj.5000320

42. Tai M, Tan HY, Yong YK, Shankar EM, Viswanathan S, Nor HM, Rahmat K, Yap JF, Ng BS, Tan CT (2017) Role of cytokines in the assessment of clinical outcome and neuroimaging findings in patients with tuberculous meningitis. Neurology Asia 22 (3):209-220

43. Tas D, Okutan O, Caliskan T, Ipcioglu OM, Ciftci F, Kartaloglu Z (2009) VASCULAR ENDOTHELIAL GROWTH FACTOR FOR DIFFERENTIAL DIAGONSIS OF NONMALIGNANT PLEURAL EFFUSIONS. Nobel Medicus 5 (3):40-44

44. Teixeira LR, Dias MB, Sales RK, Antonangelo L, Alvarenga VA, Puka J, Marchi E, Acencio MM (2016) Profile of Metalloproteinases and Their Association with Inflammatory Markers in Pleural Effusions. Lung 194 (6):1021-1027. doi:10.1007/s00408-016-9945-5

45. Tomimoto H, Yano S, Muguruma H, Kakiuchi S, Sone S (2007) Levels of soluble vascular endothelial growth factor receptor 1 are elevated in the exudative pleural effusions. The journal of medical investigation : JMI 54 (1-2):146-153. doi:10.2152/jmi.54.146

46. van der Flier M, Hoppenreijs S, van Rensburg AJ, Ruyken M, Kolk AH, Springer P, Hoepelman AI, Geelen SP, Kimpen JL, Schoeman JF (2004) Vascular endothelial growth factor and blood-brain barrier disruption in tuberculous meningitis. The Pediatric infectious disease journal 23 (7):608-613. doi:10.1097/01.inf.0000131634.57368.45

47. Visser DH, Solomons RS, Ronacher K, van Well GT, Heymans MW, Walzl G, Chegou NN, Schoeman JF, van Furth AM (2015) Host immune response to tuberculous meningitis. Clinical infectious diseases : an official publication of the Infectious Diseases Society of America 60 (2):177-187. doi:10.1093/cid/ciu781

48. Wang S, Li Y, Shen Y, Wu J, Gao Y, Zhang S, Shao L, Jin J, Zhang Y, Zhang W (2018) Screening and identification of a six-cytokine biosignature for detecting TB infection and discriminating active from latent TB. Journal of translational medicine 16 (1):206. doi:10.1186/s12967-018-1572-x

49. Xue K, Xiong S, Xiong W (2007) Clinical value of vascular endothelial growth factor combined with interferon-gamma in diagnosing malignant pleural effusion and tuberculous pleural effusion. Journal of Huazhong University of Science and Technology Medical sciences = Hua zhong ke ji da xue xue bao Yi xue Ying De wen ban = Huazhong keji daxue xuebao Yixue Yingdewen ban 27 (5):495-497. doi:10.1007/s11596-007-0504-4

50. Zhan N, Dong W-G, Wang J (2016) The clinical significance of vascular endothelial growth factor in malignant ascites. Tumor Biology 37 (3):3719-3725

51. Zhang Y, Yu LK, Lu GJ, Xia N, Xie HY, Hu W, Hao KK, Xu CH, Qian Q (2014) Prognostic values of VEGF and endostatin with malignant pleural effusions in patients with lung cancer. Asian Pacific journal of cancer prevention : APJCP 15 (19):8435-8440. doi:10.7314/apjcp.2014.15.19.8435

52. Zhou WB, Bai M, Jin Y (2009) Diagnostic value of vascular endothelial growth factor and endostatin in malignant pleural effusions. The international journal of tuberculosis and lung disease : the official journal of the International Union against Tuberculosis and Lung Disease 13 (3):381-386
