# Supplementary material for: Vascular endothelial growth factor levels in tuberculosis: A systematic review and meta-analysis
Source: PLoS One. 2022 May 25;17(5):e0268543. doi: 10.1371/journal.pone.0268543 (PMC9132289; doi:10.1371/journal.pone.0268543)
Supplement: S2 Table — (DOCX) [file pone.0268543.s025.docx]

| Study | Selection | | | | Comparability | | Outcome | | Total |
| --- | --- | --- | --- | --- | --- | --- | --- | --- | --- |
|  | Case definition | Representativeness | Selection of Control | Definition of Control | Age | Sex | Assessment of outcome | Statistical test |  |
| Abe et al. 2001 [1] | * | * | -- | -- | -- | -- | ** | * | 5 |
| Ahmad et al. 2019 [2] | * | * | * | * | -- | * | ** | * | 8 |
| Alatas et al. 2004 [3] | * | -- | -- | -- | * | -- | ** | * | 5 |
| Antonangelo et al. 2012 [4] | * | -- | NA (*) | NA (*) | NA (*) | NA (*) | ** | * | 8 |
| Bayram et al. 2018 [5] | * | * | * | * | * | -- | ** | * | 8 |
| Bhat et al. 2019 [6] | * | * | -- | -- | * | * | ** | * | 7 |
| Chen et al. 2003 [7] | -- | -- | -- | -- | -- | -- | ** | * | 3 |
| Daniil et al. 2007 [8] | * | -- | * | * | -- | -- | ** | * | 6 |
| Djoba Siawaya et al. 2009 [9] | * | -- | * | * | * | -- | ** | * | 7 |
| Dong et al. 2003 [10] | * | -- | * | * | -- | * | ** | * | 7 |
| Fathi et al. 2014 [11] | * | -- | * | * | -- | -- | ** | * | 6 |
| Fricke et al. 2014 [12] | * | * | * | * | * | * | ** | * | 9 |
| Hamed et al. 2004 [13] | * | -- | * | * | * | -- | ** | * | 7 |
| Husain et al. 2008 [14] | * | -- | * | * | * | * | ** | * | 8 |
| Im et al. 2016 [15] | * | -- | * | * | * | * | ** | * | 8 |
| Jankowska et al. 2002 [16] | * | -- | * | * | -- | -- | ** | * | 6 |
| Jin et al. 2004 [17] | * | -- | * | * | * | * | ** | * | 8 |
| Kalomenidis et al. 2006 [18] | * | -- | * | * | -- | -- | ** | * | 6 |
| Kaya et al. 2005 [19] | * | -- | * | * | * | -- | ** | * | 7 |
| Khalil et al. 2017 [20] | * | -- | * | * | -- | -- | * | * | 6 |
| Kim et al. 2017 [21] | * | -- | * | * | * | * | ** | * | 8 |
| Kiropoulos et al. 2005 [22] | * | -- | * | * | * | * | ** | * | 8 |
| Li et al. 2020 [23] | * | * | -- | -- | * | * | ** | * | 7 |
| Lim et al. 2000 [24] | * | -- | * | * | * | * | ** | * | 8 |
| Liu et al. 2010 [25] | * | * | * | * | * | * | ** | * | 9 |
| Matsuyama et al. 2001 [26] | * | -- | * | * | * | * | ** | * | 8 |
| Mihret et al. 2013 [27] | * | -- | * | * | * | * | ** | * | 8 |
| Misra et al. 2013 [28] | * | * | -- | -- | * | * | ** | * | 7 |
| Momi et al. 2002 [29] | * | * | * | * | -- | -- | ** | * | 7 |
| Omar et al. 2013 [30] | * | * | -- | -- | * | * | ** | * | 7 |
| Polena et al. 2016 [31] | * | -- | -- | -- | -- | -- | ** | * | 4 |
| Qama et al. 2012 [32] | * | -- | -- | * | * | * | ** | * | 7 |
| Qian et al. 2012 [33] | * | -- | * | * | -- | * | ** | * | 7 |
| Qiu et al. 2009 [34] | -- | -- | -- | -- | -- | -- | ** | * | 3 |
| Ranaivomanana et al. 2018 [35] | * | -- | -- | * | * | * | ** | * | 7 |
| Ruiz et al. 2005 [36] | * | * | * | * | -- | -- | ** | * | 7 |
| Sack et al. 2005 [37] | * | * | * | * | * | * | ** | * | 9 |
| Safe et al. 2021 [38] | * | * | * | * | -- | -- | ** | * | 7 |
| Saraya et al. 2018 [39] | * | -- | * | * | * | * | -- | * | 6 |
| Seiscento et al. 2010 [40] | * | -- | * | * | * | * | ** | * | 8 |
| Shen et al. 2015 [41] | * | -- | * | * | * | * | ** | * | 8 |
| Tai et al. 2017 [42] | * | -- | NA (*) | NA (*) | NA (*) | NA (*) | ** | * | 8 |
| Tas et al. 2009 [43] | * | -- | * | * | * | * | ** | * | 8 |
| Teixeira et al. 2016 [44] | * | -- | * | * | * | * | ** | * | 8 |
| Tomimoto et al. 2007 [45] | * | -- | * | * | -- | -- | ** | * | 6 |
| van der Flier et al. 2004 [46] | * | -- | * | * | * | * | ** | * | 8 |
| Visser et al. 2015 [47] | * | * | * | * | * | * | ** | * | 9 |
| Wang et al. 2018 [48] | * | * | * | * | * | * | ** | * | 9 |
| Xue et al. 2007 [49] | * | -- | * | * | * | * | ** | * | 8 |
| Zhan et al. 2016 [50] | * | * | * | * | * | * | ** | * | 9 |
| Zhang et al. 2014 [51] | * | -- | * | * | -- | * | ** | * | 7 |
| Zhou et al. 2009 [52] | * | -- | * | * | * | * | ** | * | 8 |
