# Supplementary material for: Vascular endothelial growth factor levels in tuberculosis: A systematic review and meta-analysis
Source: PLoS One. 2022 May 25;17(5):e0268543. doi: 10.1371/journal.pone.0268543 (PMC9132289; doi:10.1371/journal.pone.0268543)
Supplement: S3 Table — (DOCX) [file pone.0268543.s026.docx]

| **Comparison** | **Predictor** | **Subgroup** | **Cases, n** | **Controls, n** | **SMD [95% CI]** | **Heterogeneity for subgroups** | | |
| --- | --- | --- | --- | --- | --- | --- | --- | --- |
|  |  |  |  |  |  | Q statistic | τ² | I² |
| Pleural vs. serum VEGF levels in TB patients | Location | East Asia & Pacific | 190 | 193 | 1.8680 [ 0.5687; 3.1673] | 98.47 | 3.32 | 92.9% |
|  |  | Europe & Central Asia | 56 | 56 | -0.1133 [-2.2134; 1.9867] | 9.81 | 3.32 | 79.6% |
|  |  | Latin America & Caribbean | 39 | 39 | 0.4588 [-3.1399; 4.0576] | 0.00 | -- | -- |
|  |  | Middle East & North Africa | 21 | 21 | 1.9304 [-0.8262; 4.6870] | 8.73 | 3.32 | 88.6 |
| Blood VEGF levels in TB vs. healthy control | Location | East Asia & Pacific | 375 | 440 | 1.7245 [0.7178; 2.7311] | 265.58 | 1.4811 | 98.1% |
|  |  | Sub-Saharan Africa, East Asia & Pacific | 124 | 19 | 0.6810 [-1.7539; 3.1160] | 0.00 | -- | -- |
|  |  | Europe & Central Asia | 89 | 55 | 1.1360 [-0.2916; 2.5635] | 1.67 | 1.4811 | 0.0% |
|  |  | South Asia | 120 | 60 | 2.1779 [-0.2382; 4.5939] | 0.00 | -- | -- |
|  |  | Sub-Saharan Africa | 92 | 71 | 0.0929 [-1.3262; 1.5120] | 21.87 | 1.4811 | 90.9% |
|  |  | Middle East & North Africa | 40 | 25 | 1.7585 [-0.0468; 3.5637] | 5.03 | 1.4811 | 80.1% |
|  | Sample | Serum | 748 | 599 | 1.5457 [ 0.9287; 2.1627] | 305.10 | 1.1774 | 96.1% |
|  |  | Plasma | 92 | 71 | 0.0964 [-1.1783; 1.3710] | 21.87 | 1.1774 | 90.9% |
|  | Assay | ELISA | 549 | 409 | 1.6861 [ 1.0236; 2.3485] | 244.33 | 1.1306 | 95.9% |
|  |  | Not ELISA | 291 | 261 | 0.3987 [-0.5607; 1.3581] | 31.05 | 1.1306 | 87.1% |
| Blood VEGF levels in TPE vs. MPE | Location | East Asia & Pacific | 193 | 497 | -1.5529 [-2.8032; -0.3025] | 162.04 | 3.1336 | 95.7% |
|  |  | Middle East & North Africa | 21 | 25 | -1.7819 [-4.3440; 0.7802] | 0.95 | 3.1336 | 0.0% |
|  |  | Europe & Central Asia | 41 | 136 | 0.1129 [-2.3698; 2.5955] | 4.29 | 3.1336 | 76.7% |
| Pleural VEGF levels in TPE vs. transudate, CHF/CRF, or cirrhotic effusion | Location | Europe & Central Asia | 283 | 219 | 1.3414 [0.9210; 1.7618] | 36.78 | 0.4810 | 61.9% |
|  |  | Middle East & North Africa | 32 | 35 | 2.6748 [1.5440; 3.8056] | 17.35 | 0.4810 | 88.5% |
| Pleural VEGF levels in TPE vs. MPE | Location | Latin America & Caribbean | 28 | 25 | -0.5867 [-3.4189; 2.2455] | 0.00 | -- | -- |
|  |  | East Asia & Pacific | 320 | 592 | -1.6801 [-2.4786; -0.8815] | 198.09 | 2.0089 | 93.9% |
|  |  | Middle East & North Africa | 32 | 60 | -2.6090 [-4.3333; -0.8846] | 25.61 | 2.0089 | 92.2% |
|  |  | Europe & Central Asia | 114 | 320 | -0.8602 [-1.8727; 0.1523] | 2.64 | 2.0089 | 0.0% |
| Pleural VEGF levels in TPE vs. PPE | Location | Europe & Central Asia | 127 | 162 | -0.4960 [-0.9593; -0.0327] | 17.69 | 0.2786 | 60.4% |
|  |  | Middle East & North Africa | 6 | 7 | -1.9416 [-3.6905; -0.1927] | 0.00 | -- | -- |
|  |  | East Asia & Pacific | 60 | 55 | 0.2353 [-0.5145; 0.9852] | 7.49 | 0.2786 | 73.3% |
