# Supplementary material for: Vascular endothelial growth factor levels in tuberculosis: A systematic review and meta-analysis
Source: PLoS One. 2022 May 25;17(5):e0268543. doi: 10.1371/journal.pone.0268543 (PMC9132289; doi:10.1371/journal.pone.0268543)
Supplement: S4 Table — (DOCX) [file pone.0268543.s027.docx]

| **Comparison** | **Moderator** | **Estimated index** | **SE** | **95% CI** | **p-value** |
| --- | --- | --- | --- | --- | --- |
| Pleural vs. serum VEGF levels in TB | The male parentage of patients | 0.062 | 0.048 | -0.033 to 0.157 | 0.2018 |
|  | The mean age of patients | -0.013 | 0.022 | -0.056 to 0.030 | 0.5519 |
| Blood VEGF levels in TB vs. healthy control | The male parentage of patients | -0.0099 | 0.0348 | -0.0781 to 0.0583 | 0.7758 |
|  | The mean age of patients | 0.0540 | 0.0421 | -0.0285 to 0.1366 | 0.1994 |
| Blood VEGF levels in TPE vs. MPE | The male parentage of patients | -0.0093 | 0.0633 | -0.1335 to 0.1149 | 0.8833 |
|  | The mean age of patients | 0.0770 | 0.0272 | 0.0237 to 0.1304 | 0.0047 |
| Pleural VEGF levels in TPE vs. transudate, CHF/CRF, or cirrhotic effusion | The male parentage of patients | -0.0367 | 0.0246 | -0.0849 to 0.0114 | 0.1349 |
|  | The mean age of patients | -0.0078 | 0.0199 | -0.0468 to 0.0311 | 0.6941 |
| Pleural VEGF levels in TPE vs. MPE | The male parentage of patients | 0.0315 | 0.0373 | -0.0416 to 0.1046 | 0.3987 |
|  | The mean age of patients | 0.0334 | 0.0218 | -0.0094 to 0.0761 | 0.1263 |
| Pleural VEGF levels in TPE vs. PPE | The male parentage of patients | 0.0391 | 0.0278 | -0.0154 to 0.0935 | 0.1596 |
|  | The mean age of patients | -0.0043 | 0.0182 | -0.0400 to 0.0313 | 0.8117 |
