# Supplementary material for: Vascular endothelial growth factor levels in tuberculosis: A systematic review and meta-analysis
Source: PLoS One. 2022 May 25;17(5):e0268543. doi: 10.1371/journal.pone.0268543 (PMC9132289; doi:10.1371/journal.pone.0268543)
Supplement: S1 Text — (DOCX) [file pone.0268543.s028.docx]

**Supplementary results**

With the detailed review, 74 articles were excluded due to the following reasons; no sufficient data provided for meta-analysis [1-15], not about VEGF [16-39], reported MFI [40] or absorbance [41], not concentrations, measured VGEF mRNA levels, not protein levels [42,43], monitored VEGF levels during anti-retroviral therapy, not anti-tuberculosis therapy [44], inappropriate patient group (included patients with drug-resistant TB [45], TB co-existed with other infections [46], post-TB COPD [47], or without TB [48,49]), measured VEGF in lung tissue [50], tuberculoma [51,52], tear [53], saliva [54-56], wound drainage fluid [57], PBMC [58], QFT supernatants [59-62], or whole blood cultured in TB-IGRA [63], reported VEGF levels in pleural/peritoneal fluid as one mixed group, not separately for each pleural or peritoneal fluid [64], no within or between group comparison of interest (measured VEGF in patients with TB of different lineages [65], patiens with and without residual pleural thickening [66], patients with loculated and non-loculated pleural effusion [67], HIV-infected patients vs. HIV-uninfected patients [68], or patients with active TB vs. old TB vs. drug-resistant TB vs. acute bronchitis [69]), duplicate data [70,71], wrong design (mother-infant study [72] or *in vitro* study [73]), wrong type of study (book chapter [74]).

1. Albuquerque VVS, Kumar NP, Fukutani KF, Vasconcelos B, Arriaga MB, Silveira-Mattos PS, Babu S, Andrade BB (2019) Plasma levels of C-reactive protein, matrix metalloproteinase-7 and lipopolysaccharide-binding protein distinguish active pulmonary or extrapulmonary tuberculosis from uninfected controls in children. Cytokine 123. doi:10.1016/j.cyto.2019.154773

2. Clark DV, Banura P, Bandeen-Roche K, Liles WC, Kain KC, Scheld WM, Moss WJ, Jacob ST (2019) Biomarkers of endothelia activation/dysfunction distinguish subgroups of Ugandan patients with sepsis and differing mortality risks. Jci Insight 4 (10). doi:10.1172/jci.insight.127623

3. Djoba Siawaya JF, Beyers N, van Helden P, Walzl G (2009) Differential cytokine secretion and early treatment response in patients with pulmonary tuberculosis. Clinical and experimental immunology 156 (1):69-77. doi:10.1111/j.1365-2249.2009.03875.x

4. Heslop R, Bojang AL, Jarju S, Mendy J, Mulwa S, Secka O, Mendy FS, Owolabi O, Kampmann B, Sutherland JS (2016) Changes in Host Cytokine Patterns of TB Patients with Different Bacterial Loads Detected Using 16S rRNA Analysis. PloS one 11 (12):e0168272. doi:10.1371/journal.pone.0168272

5. Hur YG, Kang YA, Jang SH, Hong JY, Kim A, Lee SA, Kim Y, Cho SN (2015) Adjunctive biomarkers for improving diagnosis of tuberculosis and monitoring therapeutic effects. The Journal of infection 70 (4):346-355. doi:10.1016/j.jinf.2014.10.019

6. Kathamuthu GR, Moideen K, Baskaran D, Sekar G, Rathinam S, Bharathi VJ, Ganeshan GR, Babu S (2018) Tuberculous lymphadenitis is associated with altered levels o circulating angiogenic factors. International Journal of Tuberculosis and Lung Disease 22 (5):557-+. doi:10.5588/ijtld.17.0609

7. Keynan Y, Rueda ZV, Aguilar Y, Trajtman A, Vélez LA (2015) Unique cytokine and chemokine patterns in bronchoalveolar lavage are associated with specific causative pathogen among HIV infected patients with pneumonia, in Medellin, Colombia. Cytokine 73 (2):295-301. doi:10.1016/j.cyto.2015.03.003

8. Kim SH, Lee WY, Park JY, Park HS, Han HK, Ju HS, Hong TW, Lee NW, Shin KC, Yong SJ (2003) Diagnostic Value of C-Veactive Protein and Vascular Endothelial Growth Factor in Differentiation of Pleural Effusions. Tuberculosis and Respiratory Diseases 55 (5):467-477. doi:10.4046/trd.2003.55.5.467

9. Kumar NP, Anuradha R, Andrade BB, Suresh N, Ganesh R, Shankar J, Kumaraswami V, Nutman TB, Babu S (2013) Circulating biomarkers of pulmonary and extrapulmonary tuberculosis in children. Clinical and Vaccine Immunology 20 (5):704-711. doi:10.1128/CVI.00038-13

10. Kumar NP, Banurekha VV, Nair D, Babu S (2016) Circulating Angiogenic Factors as Biomarkers of Disease Severity and Bacterial Burden in Pulmonary Tuberculosis. PloS one 11 (1):e0146318. doi:10.1371/journal.pone.0146318

11. Kumar NP, Moideen K, Sivakumar S, Menon PA, Viswanathan V, Kornfeld H, Babu S (2017) Tuberculosis-diabetes co-morbidity is characterized by heightened systemic levels of circulating angiogenic factors. The Journal of infection 74 (1):10-21. doi:10.1016/j.jinf.2016.08.021

12. Loxton NW, Rohlwink UK, Tshavhungwe M, Dlamini L, Shey M, Enslin N, Figaji A (2021) A pilot study of inflammatory mediators in brain extracellular fluid in paediatric TBM. PloS one 16 (3 March). doi:10.1371/journal.pone.0246997

13. Nikolayevskyy V, Balabanova Y, Kontsevaya I, Ignatyeva O, Skenders G, Vasiliauskiene E, Bockel DV, Drobniewski F (2020) Biomarkers of treatment success in fully sensitive pulmonary tuberculosis patients: A multicenter longitudinal study. Biomarkers in Medicine 14 (15):1439-1452. doi:10.2217/bmm-2020-0246

14. Ota MO, Mendy JF, Donkor S, Togun T, Daramy M, Gomez MP, Chegou NN, Sillah AK, Owolabi O, Kampmann B, Walzl G, Sutherland JS (2014) Rapid diagnosis of tuberculosis using ex vivo host biomarkers in sputum. The European respiratory journal 44 (1):254-257. doi:10.1183/09031936.00209913

15. Park HY, Hahm CR, Jeon K, Koh WJ, Suh GY, Chung MP, Kim H, Kwon OJ, Um SW (2012) Serum Vascular Endothelial Growth Factor and Angiopoietin-2 Are Associated with the Severity of Systemic Inflammation Rather than the Presence of Hemoptysis in Patients with Inflammatory Lung Disease. Yonsei Medical Journal 53 (2):369-376. doi:10.3349/ymj.2012.53.2.369

16. Ceyhan BB, Demiralp E, Karakurt ZL, Karakurt S, Sungur M (2003) Transforming growth factor beta-1 level in pleural effusion. Respirology (Carlton, Vic) 8 (3):321-325. doi:10.1046/j.1440-1843.2003.00474.x

17. Chen KY, Feng PH, Chang CC, Chen TT, Chuang HC, Lee CN, Su CL, Lin LY, Lee KY (2016) Novel biomarker analysis of pleural effusion enhances differentiation of tuberculous from malignant pleural effusion. International Journal of General Medicine 9:183-189. doi:10.2147/IJGM.S100237

18. Chomej P, Bauer K, Bitterlich N, Hui DSC, Chan KS, Gosse H, Schauer J, Hoheisel G, Sack U (2004) Differential diagnosis of pleural effusions by fuzzy-logic-based analysis of cytokines. Respiratory medicine 98 (4):308-317. doi:10.1016/j.rmed.2003.10.011

19. Chung CL, Chen CH, Sheu JR, Chen YC, Chang SC (2005) Proinflammatory cytokines, transforming growth factor-β1, and fibrinolytic enzymes in loculated and free-flowing pleural exudates. Chest 128 (2):690-697. doi:10.1378/chest.128.2.690

20. Elgün S, Kaçmaz B, Durak I (2005) A potential role for nitric oxide pathway in tuberculous pleural effusion. International Journal of Tuberculosis and Lung Disease 9 (3):339-343

21. Gönlügür T, Gönlügür U (2008) The comparison of the biochemical analyses of pleural fluid in tuberculosis and parapneumonic effusions. Goztepe Tip Dergisi 23 (3):81-84

22. Gu Y, Zhai K, Shi HZ (2016) Clinical value of tumor markers for determining cause of pleural effusion. Chinese Medical Journal 129 (3):253-258. doi:10.4103/0366-6999.174501

23. Kalita J, Misra UK, Bhoi SK, Chauhan PS, Sagar B (2017) Possible role of transforming growth factor β in tuberculous meningitis. Cytokine 90:124-129. doi:10.1016/j.cyto.2016.11.004

24. Kumar NP, Velayutham B, Nair D, Babu S (2017) Angiopoietins as biomarkers of disease severity and bacterial burden in pulmonary tuberculosis. The international journal of tuberculosis and lung disease : the official journal of the International Union against Tuberculosis and Lung Disease 21 (1):93-99. doi:10.5588/ijtld.16.0565

25. Li J, Sun L, Xu F, Xiao J, Jiao W, Qi H, Shen C, Shen A (2017) Characterization of plasma proteins in children of different Mycobacterium tuberculosis infection status using label-free quantitative proteomics. Oncotarget 8 (61):103290-103301. doi:10.18632/oncotarget.21179

26. Mizuno K, Matsuyama W, Mitsuyama H, Watanabe M, Higashimoto I, Osame M, Arimura K (2005) Clinical investigation: Increased serum stromal derived factor 1 alpha levels in pulmonary tuberculosis. Clinical and experimental immunology 139 (3):490-497. doi:10.1111/j.1365-2249.2005.02721.x

27. Qian Q, Sun WK, Zhan P, Zhang Y, Song Y, Yu LK (2012) Role of monocyte chemoattractant protein-1, tumor necrosis factor-α and interleukin-6 in the control of malignant pleural effusion and survival in patients with primary lung adenocarcinoma. International Journal of Biological Markers 27 (2):118-124. doi:10.5301/JBM.2012.9197

28. San José ME, Valdes L, Gonzalez-Barcala FJ, Vizcaino L, Garrido M, Sanmartin A, Mougan S, Pose A, Segade A (2010) Diagnostic value of proinflammatory interleukins in parapneumonic effusions. American Journal of Clinical Pathology 133 (6):884-891. doi:10.1309/AJCPB67PYKVRVPPR

29. Shivakoti R, Dalli J, Kadam D, Gaikwad S, Barthwal M, Colas RA, Mazzacuva F, Lokhande R, Dharmshale S, Bharadwaj R, Kagal A, Pradhan N, Deshmukh S, Atre S, Sahasrabudhe T, Kakrani A, Kulkarni V, Raskar S, Suryavanshi N, Chon S, Gupte A, Gupta A, Gupte N, Arriaga MB, Fukutani KF, Andrade BB, Golub JE, Mave V (2020) Lipid mediators of inflammation and Resolution in individuals with tuberculosis and tuberculosis-Diabetes. Prostaglandins and Other Lipid Mediators 147. doi:10.1016/j.prostaglandins.2019.106398

30. Shu CC, Wang JY, Wu MF, Lai HC, Chiang BL, Yu CJ (2018) Interleukin 23/interleukin 17 axis activated by Mycobacterium avium complex (MAC) is attenuated in patients with MAC-lung disease. Tuberculosis 110:7-14. doi:10.1016/j.tube.2018.03.001

31. Tang Y, Zhang J, Huang H, He X, Zhang J, Ou M, Li G, Zeng C, Ye T, Ren L, Liu Y, Zhang G (2019) Pleural IFN-γ release assay combined with biomarkers distinguished effectively tuberculosis from malignant pleural effusion. BMC infectious diseases 19 (1). doi:10.1186/s12879-018-3654-z

32. Vatansever S, Gelisgen R, Uzun H, Yurt S, Kosar F (2009) Potential role of matrix metalloproteinase-2,-9 and tissue inhibitors of metalloproteinase-1,-2 in exudative pleural effusions. Clinical and Investigative Medicine 32 (4):E293-E300

33. Wang C, Wei LL, Shi LY, Pan ZF, Yu XM, Li TY, Liu CM, Ping ZP, Jiang TT, Chen ZL, Mao LG, Li ZJ, Li JC (2015) Screening and identification of five serum proteins as novel potential biomarkers for cured pulmonary tuberculosis. Scientific reports 5. doi:10.1038/srep15615

34. Yetkin O, Tek I, Yetkin F, Numanoglu N (2007) Role of pleural viscosity in the differential diagnosis of exudative pleural effusion. Respirology (Carlton, Vic) 12 (2):267-271. doi:10.1111/j.1440-1843.2006.01041.x

35. Yu CJ, Wang CL, Wang CI, Chen CD, Dan YM, Wu CC, Wu YC, Lee IN, Tsai YH, Chang YS, Yu JS (2011) Comprehensive proteome analysis of malignant pleural effusion for lung cancer biomarker discovery by using multidimensional protein identification technology. Journal of Proteome Research 10 (10):4671-4682. doi:10.1021/pr2004743

36. Zarogiannis SG, Tsilioni I, Hatzoglou C, Molyvdas PA, Gourgoulianis KI (2013) Pleural fluid protein is inversely correlated with age in uncomplicated parapneumonic pleural effusions. Clinical Biochemistry 46 (4-5):378-380. doi:10.1016/j.clinbiochem.2012.11.024

37. Zhang J, Chen Y, He G, Jiang X, Chen P, Ouyang J (2020) Differential diagnosis of tuberculous and malignant pleural effusions: comparison of the Th1/Th2 cytokine panel, tumor marker panel and chemistry panel. Scandinavian Journal of Clinical and Laboratory Investigation 80 (4):265-270. doi:10.1080/00365513.2020.1728784

38. Zielonka TM, Demkow U, Michalowska-Mitczuk D, Filewska M, Bialas B, Zycinska K, Obrowski MH, Kus J, Skopinska-Rozewska E (2011) Angiogenic activity of sera from pulmonary tuberculosis patients in relation to IL-12p40 and TNFα serum levels. Lung 189 (4):351-357. doi:10.1007/s00408-011-9291-6

39. Eum SY, Jeon BY, Min JH, Kim SC, Cho S, Park SK, Cho SN (2008) Tumor necrosis factor-alpha and interleukin-10 in whole blood is associated with disease progression in pulmonary mulitdrug-resistant tuberculosis patients. Respiration; international review of thoracic diseases 76 (3):331-337. doi:10.1159/000113932

40. de Melo MGM, Mesquita EDD, Oliveira MM, da Silva-Monteiro C, Silveira AKA, Malaquias TS, Dutra TCP, Galliez RM, Kritski AL, Silva EC (2018) Imbalance of NET and Alpha-1-Antitrypsin in Tuberculosis Patients Is Related With Hyper Inflammation and Severe Lung Tissue Damage. Frontiers in immunology 9:3147. doi:10.3389/fimmu.2018.03147

41. Manyelo CM, Solomons RS, Snyders CI, Manngo PM, Mutavhatsindi H, Kriel B, Stanley K, Walzl G, Chegou NN (2019) Application of Cerebrospinal Fluid Host Protein Biosignatures in the Diagnosis of Tuberculous Meningitis in Children from a High Burden Setting. Mediators of inflammation 2019:7582948. doi:10.1155/2019/7582948

42. Cha N, Lv M, Zhao YJ, Yang D, Wang EH, Wu GP (2014) Diagnostic utility of VEGF mRNA and SP1 mRNA expression in bronchial cells of patients with lung cancer. Respirology (Carlton, Vic) 19 (4):544-548. doi:10.1111/resp.12272

43. Raju B, Hoshino Y, Belitskaya-Lévy I, Dawson R, Ress S, Gold JA, Condos R, Pine R, Brown S, Nolan A, Rom WN, Weiden MD (2008) Gene expression profiles of bronchoalveolar cells in pulmonary TB. Tuberculosis 88 (1):39-51. doi:10.1016/j.tube.2007.07.003

44. Wilkinson KA, Schneider-Luftman D, Lai R, Barrington C, Jhilmeet N, Lowe DM, Kelly G, Wilkinson RJ (2021) Antiretroviral Treatment-Induced Decrease in Immune Activation Contributes to Reduced Susceptibility to Tuberculosis in HIV-1/Mtb Co-infected Persons. Frontiers in immunology 12. doi:10.3389/fimmu.2021.645446

45. Ferrian S, Manca C, Lubbe S, Conradie F, Ismail N, Kaplan G, Gray CM, Fallows D (2017) A combination of baseline plasma immune markers can predict therapeutic response in multidrug resistant tuberculosis. PloS one 12 (5):e0176660. doi:10.1371/journal.pone.0176660

46. Venkataraman A, Kumar NP, Hanna LE, Putlibai S, Karthick M, Rajamanikam A, Sadasivam K, Sundaram B, Babu S (2021) Plasma biomarker profiling of PIMS-TS, COVID-19 and SARS-CoV2 seropositive children - a cross-sectional observational study from southern India. EBioMedicine 66:103317. doi:10.1016/j.ebiom.2021.103317

47. Guiedem E, Pefura-Yone EW, Ikomey GM, Nkenfou CN, Mesembe M, Yivala MM, Chendi BH, Jacobs GB, Chegou NN, Okomo MCA (2020) Cytokine profile in the sputum of subjects with post-tuberculosis airflow obstruction and in those with tobacco related chronic obstructive pulmonary disease. BMC immunology 21 (1):52. doi:10.1186/s12865-020-00381-w

48. Lin CH, Shu CC, Hsu CL, Cheng SL, Wang JY, Yu CJ, Lee LN (2016) The trend and the disease prediction of vascular endothelial growth factor and placenta growth factor in nontuberculous mycobacterial lung disease. Scientific reports 6. doi:10.1038/srep37266

49. Ziora D, Jastrzębski D, Adamek M, Czuba Z, Kozielski JJ, Grzanka A, Kasperska-Zajac A (2015) Circulating concentration of markers of angiogenic activity in patients with sarcoidosis and idiopathic pulmonary fibrosis. BMC pulmonary medicine 15:113. doi:10.1186/s12890-015-0110-3

50. Golubinskaya EP, Filonenko TG, Kramar TV, Yermola YA, Kubyshkin AV, Gerashenko AV, Kalfa MA, Shramko, II (2019) Dysregulation of VEGF-dependent angiogenesis in cavernous lung tuberculosis. Pathophysiology : the official journal of the International Society for Pathophysiology 26 (3-4):381-387. doi:10.1016/j.pathophys.2019.11.004

51. Gupta RK, Haris M, Husain N, Husain M, Prasad KN, Pauliah M, Srivastava C, Tripathi M, Rastogi M, Behari S, Singh A, Rathore D, Rathore RK (2007) Relative cerebral blood volume is a measure of angiogenesis in brain tuberculoma. Journal of computer assisted tomography 31 (3):335-341. doi:10.1097/01.rct.0000243443.10739.16

52. Haris M, Husain N, Singh A, Awasthi R, Singh Rathore RK, Husain M, Gupta RK (2008) Dynamic contrast-enhanced (DCE) derived transfer coefficient (ktrans) is a surrogate marker of matrix metalloproteinase 9 (MMP-9) expression in brain tuberculomas. Journal of magnetic resonance imaging : JMRI 28 (3):588-597. doi:10.1002/jmri.21491

53. Singh N, Singh R, Sharma RK, Kumar A, Sharma SP, Agarwal A, Gupta V, Singh R, Katoch D (2020) Mycobacterium Tuberculosis Modulates Fibroblast Growth Factor and Vascular Endothelial Growth Factor in Ocular Tuberculosis. Ocular immunology and inflammation:1-7. doi:10.1080/09273948.2020.1734212

54. Namuganga AR, Chegou NN, Mubiri P, Walzl G, Mayanja-Kizza H (2017) Suitability of saliva for Tuberculosis diagnosis: comparing with serum. BMC infectious diseases 17 (1):600. doi:10.1186/s12879-017-2687-z

55. Phalane KG, Kriel M, Loxton AG, Menezes A, Stanley K, van der Spuy GD, Walzl G, Chegou NN (2013) Differential expression of host biomarkers in saliva and serum samples from individuals with suspected pulmonary tuberculosis. Mediators of inflammation 2013:981984. doi:10.1155/2013/981984

56. Estévez O, Anibarro L, Garet E, Pallares Á, Pena A, Villaverde C, Del Campo V, González-Fernández Á (2020) Identification of candidate host serum and saliva biomarkers for a better diagnosis of active and latent tuberculosis infection. PloS one 15 (7):e0235859. doi:10.1371/journal.pone.0235859

57. Wang Y, Wang L, Wen Z, Wang J, Zhu Y, Chen H, Li H, Han J, Xu J, Zhang S, Hu Z, Song Y (2018) High IL-6 and VEGF-A levels correlate with delayed wound healing in cervical lymph node tuberculosis patients. The international journal of tuberculosis and lung disease : the official journal of the International Union against Tuberculosis and Lung Disease 22 (10):1227-1232. doi:10.5588/ijtld.18.0027

58. Sariko M, Maro A, Gratz J, Houpt E, Kisonga R, Mpagama S, Heysell S, Mmbaga BT, Thomas TA (2019) Evaluation of cytokines in peripheral blood mononuclear cell supernatants for the diagnosis of tuberculosis. Journal of inflammation research 12:15-22. doi:10.2147/jir.s183821

59. Chegou NN, Black GF, Kidd M, van Helden PD, Walzl G (2009) Host markers in QuantiFERON supernatants differentiate active TB from latent TB infection: preliminary report. BMC pulmonary medicine 9:21. doi:10.1186/1471-2466-9-21

60. Chegou NN, Detjen AK, Thiart L, Walters E, Mandalakas AM, Hesseling AC, Walzl G (2013) Utility of host markers detected in Quantiferon supernatants for the diagnosis of tuberculosis in children in a high-burden setting. PloS one 8 (5):e64226. doi:10.1371/journal.pone.0064226

61. Chegou NN, Sutherland JS, Namuganga AR, Corstjens PL, Geluk A, Gebremichael G, Mendy J, Malherbe S, Stanley K, van der Spuy GD, Kriel M, Loxton AG, Kriel B, Simukonda F, Bekele Y, Sheehama JA, Nelongo J, van der Vyver M, Gebrexabher A, Hailu H, Esterhuyse MM, Rosenkrands I, Aagard C, Kidd M, Kassa D, Mihret A, Howe R, Cliff JM, Crampin AC, Mayanja-Kizza H, Kaufmann SHE, Dockrell HM, Ottenhoff THM, Walzl G (2018) Africa-wide evaluation of host biomarkers in QuantiFERON supernatants for the diagnosis of pulmonary tuberculosis. Scientific reports 8 (1):2675. doi:10.1038/s41598-018-20855-7

62. Won EJ, Choi JH, Cho YN, Jin HM, Kee HJ, Park YW, Kwon YS, Kee SJ (2017) Biomarkers for discrimination between latent tuberculosis infection and active tuberculosis disease. The Journal of infection 74 (3):281-293. doi:10.1016/j.jinf.2016.11.010

63. Yao X, Liu Y, Liu Y, Liu W, Ye Z, Zheng C, Ge S (2017) Multiplex analysis of plasma cytokines/chemokines showing different immune responses in active TB patients, latent TB infection and healthy participants. Tuberculosis (Edinburgh, Scotland) 107:88-94. doi:10.1016/j.tube.2017.07.013

64. Faria DK, Faria CS, Doi D, Acencio MMP, Antonangelo L (2019) Hybrid panel of biomarkers can be useful in the diagnosis of pleural and peritoneal effusions. Clinica chimica acta; international journal of clinical chemistry 497:48-53. doi:10.1016/j.cca.2019.07.015

65. Mihret A, Bekele Y, Loxton AG, Aseffa A, Howe R, Walzl G (2012) Plasma Level of IL-4 Differs in Patients Infected with Different Modern Lineages of M. tuberculosis. Journal of tropical medicine 2012:518564. doi:10.1155/2012/518564

66. Gerogianni I, Papala M, Tsopa P, Zigoulis P, Dimoulis A, Kostikas K, Kiropoulos T, Gourgoulianis KI (2008) Could IFN-gamma predict the development of residual pleural thickening in tuberculous pleurisy? Monaldi archives for chest disease = Archivio Monaldi per le malattie del torace 69 (1):18-23. doi:10.4081/monaldi.2008.407

67. Bien MY, Wu MP, Chen WL, Chung CL (2015) VEGF correlates with inflammation and fibrosis in tuberculous pleural effusion. TheScientificWorldJournal 2015:417124. doi:10.1155/2015/417124

68. Riou C, Perez Peixoto B, Roberts L, Ronacher K, Walzl G, Manca C, Rustomjee R, Mthiyane T, Fallows D, Gray CM, Kaplan G (2012) Effect of standard tuberculosis treatment on plasma cytokine levels in patients with active pulmonary tuberculosis. PloS one 7 (5):e36886. doi:10.1371/journal.pone.0036886

69. Matsuyama W, Hashiguchi T, Matsumuro K, Iwami F, Hirotsu Y, Kawabata M, Arimura K, Osame M (2000) Increased serum level of vascular endothelial growth factor in pulmonary tuberculosis. American journal of respiratory and critical care medicine 162 (3 Pt 1):1120-1122. doi:10.1164/ajrccm.162.3.9911010

70. Sun XM, Dong WG, Gao LC (2003) Detection of VEGF levels in ascites and peritoneal fluid. Chinese Journal of Cancer Research 15 (4):310-314. doi:10.1007/BF02974898

71. Sun XM, Dong WG, Yu BP, Luo HS, Yu JP (2004) [Clinical significance of detecting VEGF, CD44v6, MMP-2, and MMP-9 in malignant ascites]. Ai zheng = Aizheng = Chinese journal of cancer 23 (1):85-89

72. Mawa PA, Webb EL, Filali-Mouhim A, Nkurunungi G, Sekaly RP, Lule SA, Prentice S, Nash S, Dockrell HM, Elliott AM, Cose S (2017) Maternal BCG scar is associated with increased infant proinflammatory immune responses. Vaccine 35 (2):273-282. doi:10.1016/j.vaccine.2016.11.079

73. Yorsangsukkamol J, Chaiprasert A, Palaga T, Prammananan T, Faksri K, Palittapongarnpim P, Prayoonwiwat N (2011) Apoptosis, production of MMP9, VEGF, TNF-alpha and intracellular growth of M. tuberculosis for different genotypes and different pks5/1 genes. Asian Pacific journal of allergy and immunology 29 (3):240-251

74. Malhotra HS, Garg RK (2017) Vascular complications of tuberculous meningitis. In: Tuberculosis of the Central Nervous System: Pathogenesis, Imaging, and Management. pp 139-155. doi:10.1007/978-3-319-50712-5_12
